# Diastereocontrol in Radical Addition to β-Benzyloxy Hydrazones: Revised Approach to Tubuvaline and Synthesis of *O*-Benzyltubulysin V Benzyl Ester

**DOI:** 10.1021/acs.joc.1c01798

**Published:** 2021-10-12

**Authors:** Manshu Li, Koushik Banerjee, Gregory K. Friestad

**Affiliations:** Department of Chemistry, University of Iowa, Iowa City, Iowa 52242, United States

## Abstract

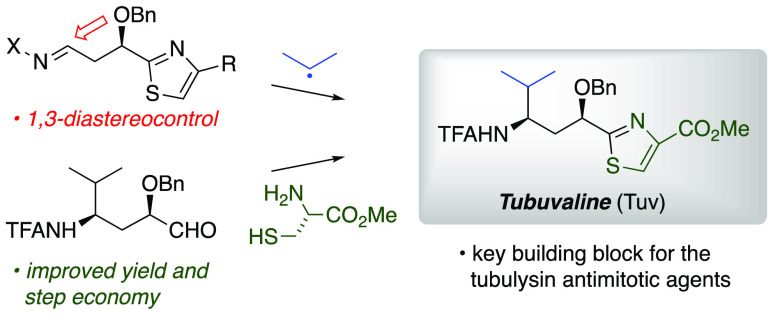

Radical addition to chiral *N*-acylhydrazones has generated unusual amino acids tubuphenylalanine (Tup) and tubuvaline (Tuv) that are structural components of the tubulysin family of picomolar antimitotic agents and previously led to a tubulysin tetrapeptide analog with a C-terminal alcohol. To improve efficiency in this synthetic route to tubulysins, and to address difficulties in oxidation of the C-terminal alcohol, here we present two alternative routes to Tuv that (a) improve step economy, (b) provide modified conditions for Mn-mediated radical addition in the presence of aromatic heterocycles, and (c) expose an example of double diastereocontrol in radical addition to a β-benzyloxyhydrazone with broader implications for asymmetric amine synthesis via radical addition. An efficient coupling sequence affords 11-*O*-benzyltubulysin V benzyl ester.

## 1 Introduction

### Stereocontrolled Mn-Mediated Radical Additions to Chiral Hydrazones

**Figure 1:**
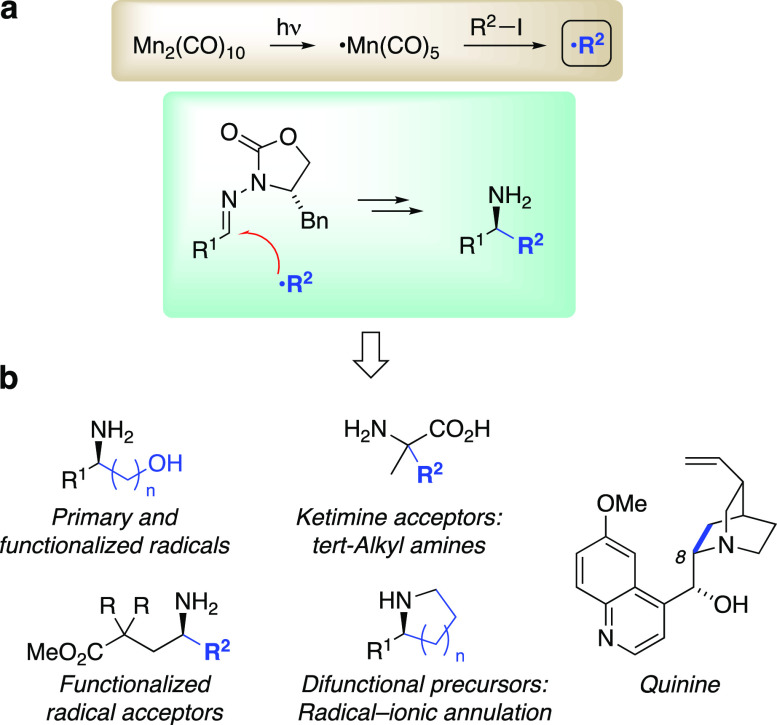
(a) Methodology for Mn-mediated radical generation and stereocontrolled addition to C=N bonds using chiral *N*-acylhydrazones. (b) Key bond constructions (blue highlights) in synthetic applications to various chiral amines.

Radical additions to imino compounds offer a useful carbon–carbon bond construction approach to chiral amine synthesis.^1^ Seminal efforts to generalize this reaction for intermolecular coupling led to stereocontrolled alkylborane-, zinc-, and tin-mediated additions by Naito,^2^ Bertrand,^3^ and our group.^4^ A significant early limitation on this chemistry restricted the scope of radicals to simple 2° and 3° alkyls, usually from reagents in large excess, due to unfavorable halogen atom transfer or competitive reduction of the radicals. Such concerns make it difficult to apply this chemistry to more complex synthetic targets, prompting our search for alternative methods to generate the radicals for this purpose. With this in mind, we initiated a long-standing effort to develop the scope of a photochemical method to initiate radical addition to imino compounds via halogen atom abstraction by ^•^Mn(CO)_5_ (Figure 1a).^5^ Meanwhile, the portfolio of radical generation conditions continues to widen^6^ and now includes a variety of photoredox catalysis methods^7^ as well as very recent approaches to Mn-mediated radical chemistry that render the processes catalytic in Mn.^8^

Our focus has been on establishing reliable and versatile modes of stereocontrol for radical addition to imino compounds, so that this type of transformation can be applied for synthesis design even when both the radical and acceptor bear structural complexity.^9,10^ Chiral *N*-acylhydrazones^11^ (Figure 1a) emerged as excellent radical acceptors that afford >95:5 diastereomer ratios for a host of intermolecular radical additions to the C=N bond. Together with the Mn-mediated conditions, this approach has been successfully applied to functionalized chiral amines (Figure 1b) such as γ-amino acids^12^ and α,α-disubstituted α-amino acids,^13^ to radical–polar crossover annulations,^14^ and to a formal synthesis of quinine.^15^ Broad vetting showed compatibility with multifunctional radical acceptors as well as multifunctional radicals, where large excesses of either component would normally be prohibitive. This feature makes Mn-mediated radical addition to chiral *N*-acylhydrazones well-suited for target-oriented synthesis and for preparing unusual amino acids and other chiral amine building blocks in the context of medicinal chemistry. These considerations drew our attention to application toward synthesis of tubulysins and analogues.

### Tubulysins

**Figure 2:**
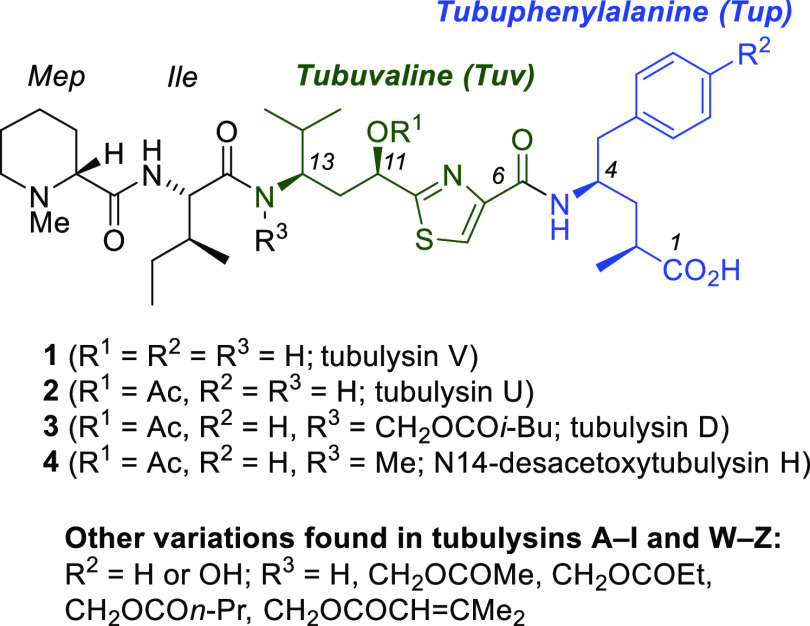
Structures of selected tubulysins, highlighting the unusual amino acids tubuvaline (green) and tubuphenylalanine (blue).

Naturally occurring peptides containing unusual amino acids offer bioactivities of potential utility in drug discovery as exemplified by dolastatin 10, plitidepsin, and didemnin B, all of which have reached Phase 2 clinical trials as cancer chemotherapeutic candidates.^16^ Similarly, the tubulysin family of antimitotic agents (Figure 2),^17^ the first examples of which were reported by Höfle et al. in 2000,^18^ are tetrapeptides of myxobacterial origin and are composed of d-*N*-methylpipecolic acid (Mep), l-isoleucine (Ile), tubuvaline (Tuv), and a C-terminal γ-amino acid, either tubuphenylalanine (Tup) or tubutyrosine (Tut).^19^ These extraordinarily active antimitotic agents rival dolastatin 10 and epothilone, with some members of the family reaching picomolar potency through a mechanism involving noncompetitive binding at the vinca domain of β-tubulin.^20^ Early evaluation of tubulysin A indicated selective cytotoxicity and potential antiangiogenic activity,^21^ although more *in vivo* studies indicated a limited therapeutic window.^22^ More recently, targeting strategies involving folate–tubulysin and antibody–tubulysin conjugates have attracted attention to address the toxicity problem.^23^

As a consequence of their potent antimitotic effects, much effort has been devoted to chemical synthesis of tubulysins,^24^ especially **1**–**4** (Figure 2), leading to several approaches to the natural products as well as numerous analogues.^25^ Creative strategies for stereocontrol have appeared in preparations of Tuv and Tup,^26^ and the *N*,*O*-acetal functionality in certain tubulysins (e.g., **3**) has also inspired new methodology.^27^ In the diverse slate of creative strategies to prepare Tuv, the main innovations tend to focus on stereocontrol issues (known to impact activity^34^) at the hydroxyl- or acetoxy-bearing center at C11, stereocontrol at the C13 chiral amine, and introduction of the thiazole. The C11 configuration has been addressed by stereocontrolled enolate oxidation,^28,29^ metalloenamine aldol addition,^30^ thiazolyl anion addition,^31^ hydride reduction,^32−35^ hetero-Diels–Alder reactions,^36^ and proline-catalyzed aldol reactions.^37^ An interesting multicomponent coupling reaction (MCR) rapidly built both C11 and the thiazole, albeit with modest stereocontrol^38,39^ that was later improved via a catalytic asymmetric Passerini reaction.^40^ Most Tuv syntheses begin with precursors having the C13 chiral amine already established from various valine derivatives and their homologues. Asymmetric induction at C13 has been accomplished by kinetic resolution of racemic aza-Michael or Mannich adducts,^33,41^ hydride reduction,^30^ and additions of various *C*-nucleophiles (enolate,^42^ organomagnesium,^43^ and allylindium reagents^44^) to imines. A nitrone cycloaddition approach established both C11 and C13 stereogenic centers.^45^ The breadth of the innovative synthetic route designs directed toward Tuv over many years attest to the challenge of this unusual multifunctional amino acid as well as the continuing interest in the tubulysins as potential cancer chemotherapeutics.^46^

**Scheme 1:**
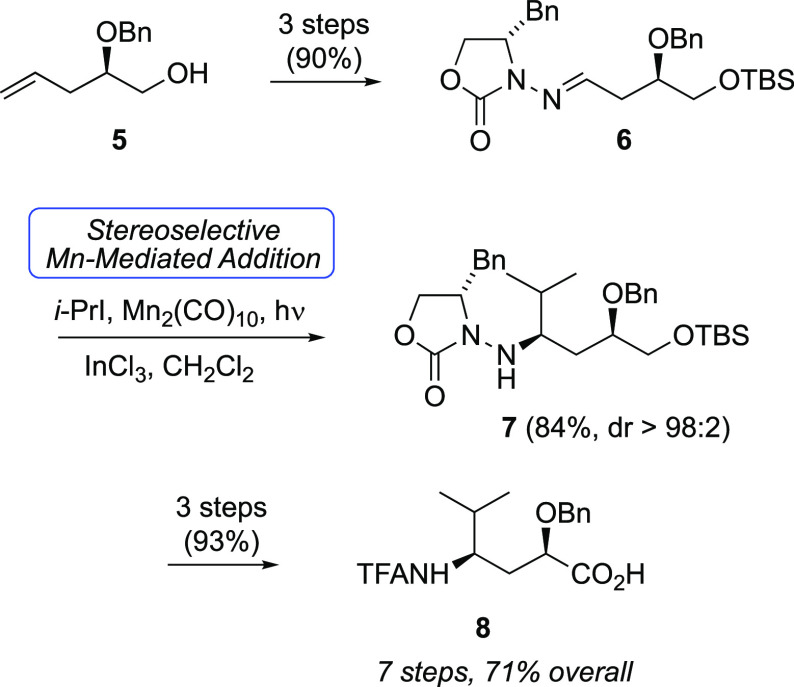


Our own efforts toward Tup and Tuv were initially published in 2004,^47^ highlighting the utility of radical addition to chiral *N*-acylhydrazones in control of the chiral amine configurations of both Tup and Tuv. Subsequent optimizations led to an efficient seven-step route to Tuv (Scheme 1).^48^ Beginning with known alcohol **5**,^49^ a three-step sequence to *N*-acylhydrazone **6** was followed by photolysis with isopropyl iodide in the presence of InCl_3_ and Mn_2_(CO)_10_. This key step efficiently furnished **7** with complete stereocontrol at the C13 chiral amine (dr >98:2, minor isomer not detected). Three functional group transformations led to γ-amino acid **8**.

**Scheme 2:**
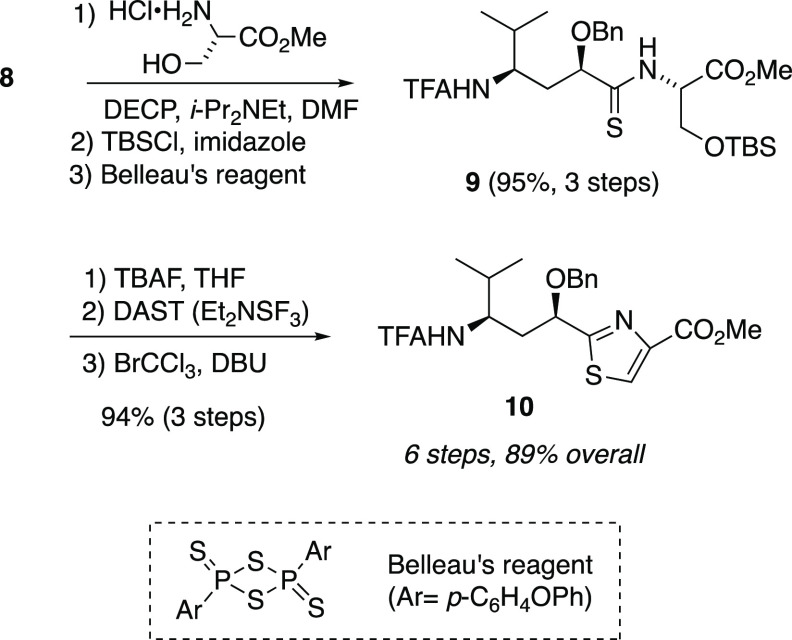


**Figure 3:**
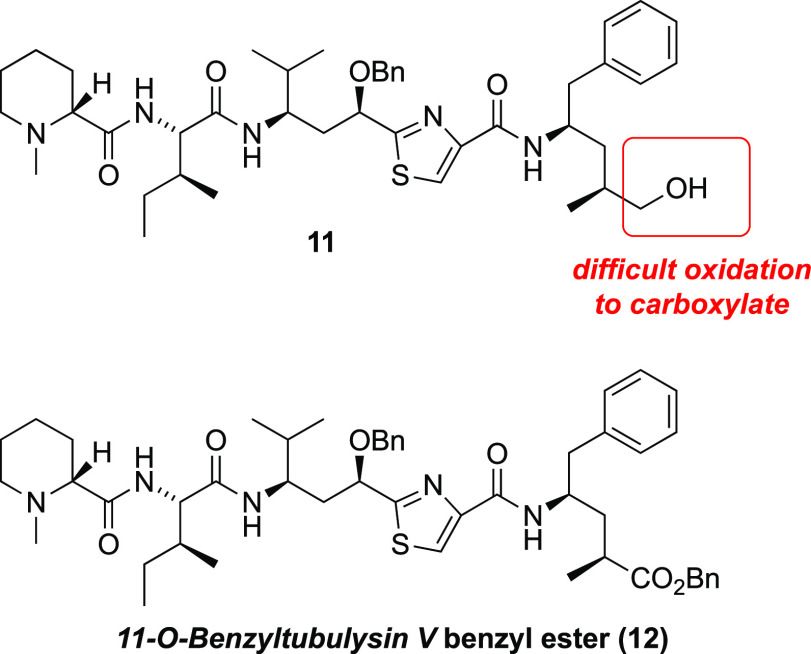
Structures of selected tubulysin analogues.

We later disclosed a high-yielding but step-intensive route to convert **8** to *N*-TFA tubuvaline methyl ester (**10**, Scheme 2) in six steps and its application in a synthesis of a tubulysin analogue with a C-terminal alcohol (**11**, Figure 3).^48^ Attempts at oxidation of the C-terminus of **11** led to complicated mixtures; a putative C-terminal aldehyde intermediate appeared to have engaged in a cyclization with the γ-amido group as a nucleophile. Thus, we considered more conventional strategies that introduced the C-terminus at the carboxylate oxidation state, along with improvements to synthesis of Tuv derivative **10**.

**Scheme 3:**
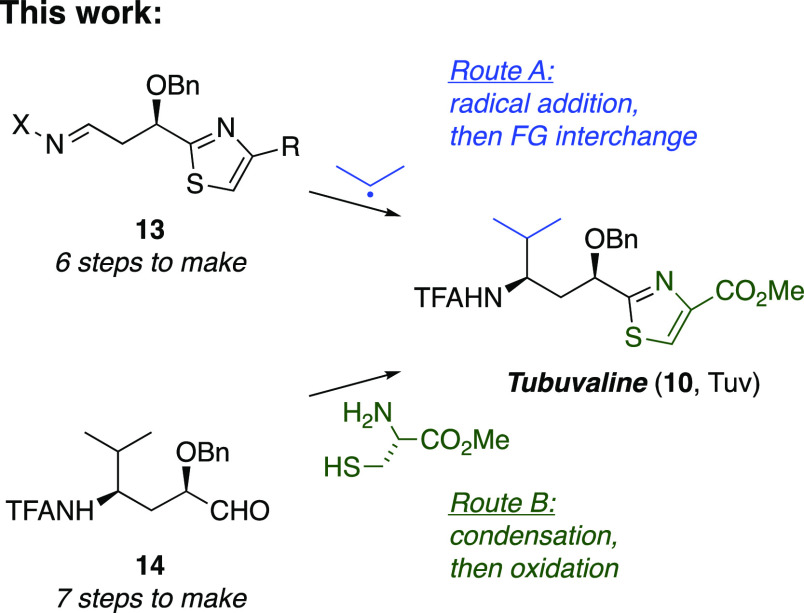


Despite the excellent overall yield of the prior route to **10**, undesirable step economics prompted development of two alternative plans to access **10** (Scheme 3). First, because constructing the tubuvaline thiazole added six steps after introduction of the chiral amine, we envisioned an improved step economy if the thiazole could be tolerated in the radical acceptor such as **13** (route A). Second, we also envisioned merging our previous Mn-mediated radical addition with a less step-intensive thiazole construction that introduced sulfur via condensation of cysteine with aldehyde **14** (route B).^50^ Here, we present the results of investigating these two routes, the former of which led to greater versatility of Mn-mediated radical addition^51^ and uncovered new insights concerning 1,3-diastereocontrol in a C=N radical acceptor and the latter of which culminated the synthesis of a dibenzyl analogue of tubulysin V (**12**, Figure 3).

## 2 Results and Discussion

### Mn-Mediated Radical Addition Compatibility with Aromatic Heterocycles

An improved step-economic synthesis of **10** according to route A (Scheme 3) required solving a problem that had emerged during prior synthetic studies on quinine.^52^ Preliminary studies in that venture revealed an incompatibility of the Mn-mediated radical addition with the basic nitrogen of a quinoline aromatic *N*-heterocycle present in the *N*-acylhydrazone radical acceptor. With this in mind, we anticipated that further modification of the Mn-mediated radical addition conditions would be needed in order to accommodate other *N*-heteroaromatics such as a thiazole.

We began this study with a screen of the effects of various aromatic heterocyclic additives on the Mn-mediated isopropyl addition to hydrazone **15** (Table 1). According to our established method, the reaction conditions include a Lewis acid (InCl_3_) to activate the acceptor toward nucleophilic radical addition.^5^ The Lewis acid coordinates in bidentate fashion to *N*-acylhydrazones, as evidenced by spectroscopic data.^12^ Various *N*-heterocycles were placed into the control reaction as additives to assess their effects on conversion and yield of isopropyl adduct **16**. The control reaction under the usual conditions (conditions A) without any additive gave **16** with a normal yield of 86% (Table 1, entry 1). In separate runs, the presence of four different aromatic *N*-heterocycles (pyridine, imidazole, benzothiazole, and thiazole **17**) in equimolar quantity each diminished the yields of the control reaction to an average of 40% (entries 2–5).

**Table 1:**
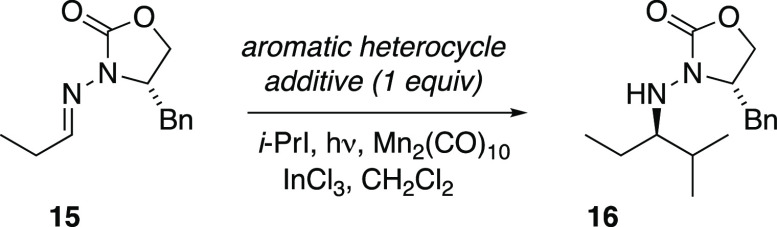


		yield (%)	
entry	additive	A^a^	B^b^
1	none	86	72
2	pyridine	44	54
3	imidazole	29	61
4	benzothiazole	24	32
5	**17**	64	70

a Conditions A: 0.11 M (hydrazone), 10 equiv of *i*-PrI, 2.2 equiv of InCl_3_.

b Conditions B: 0.017 M (hydrazone), 3 equiv of *i-*PrI, 3.5 equiv of InCl_3_.

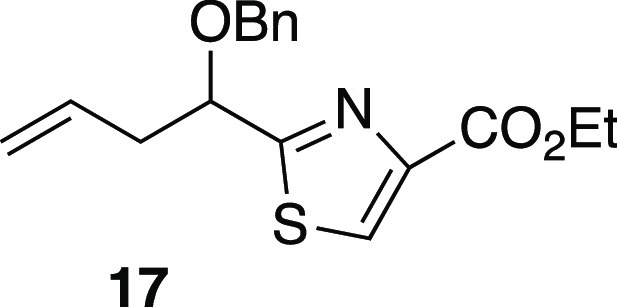

We hypothesized that diminished yields in the presence of aromatic heterocycles was attributable to basic sites of aromatic heterocycles interfering with the desired bidentate binding of InCl_3_. Modifying the conditions to obviate this problem, we found that increasing the Lewis acid loading to 3.5 equiv and diluting the reaction mixture to one-sixth of the usual concentration generally improved the results. The isolated yields of **16** in the presence of the *N*-heterocycles using these modifications (conditions B) increased to an average of 54% (Table 1). The hypothesis suggests that more basic N-heterocycles should interfere to a greater degree; indeed, pyridine and imidazole (entries 2 and 3) exhibited more substantial improvement with the modified conditions. Reactions were also cleaner under the modified conditions, with recovery of unreacted **15** accounting for much of the mass balance.

**Scheme 4:**
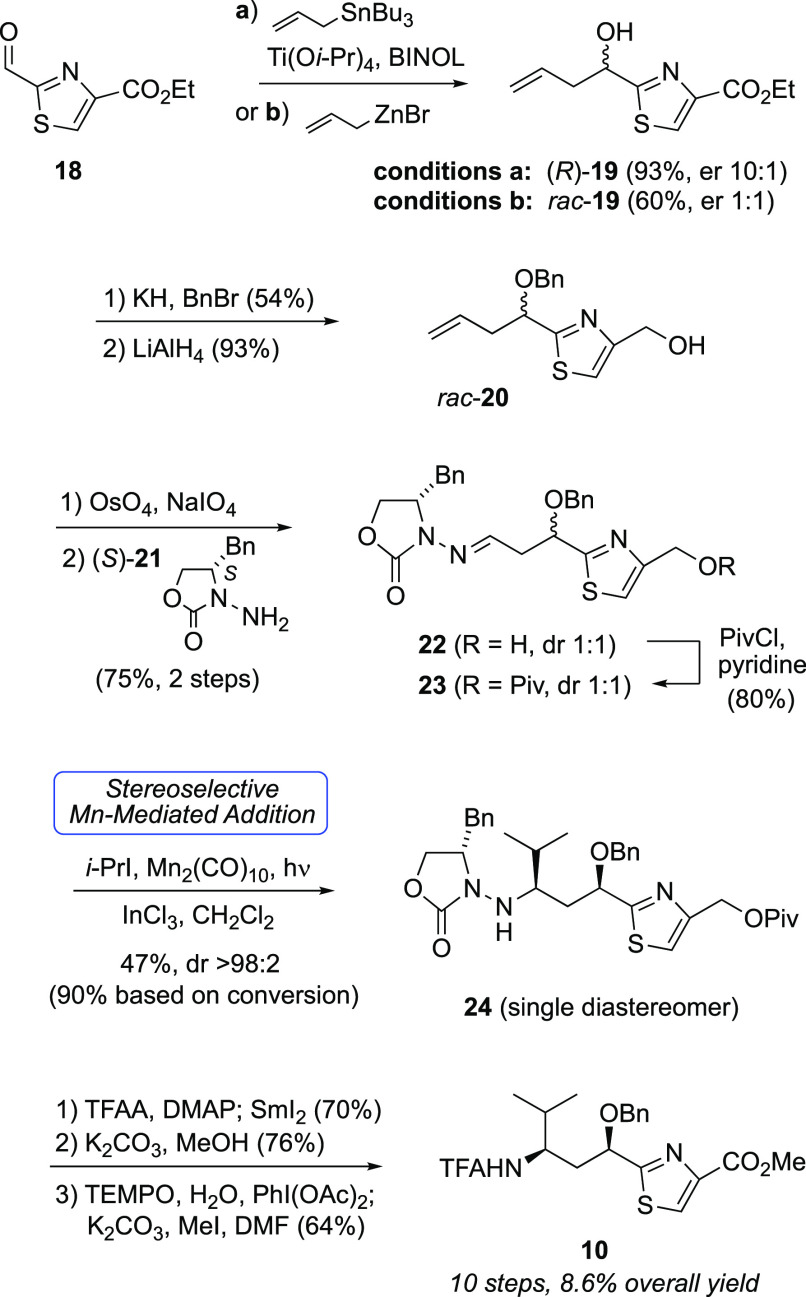


To apply these improved conditions to a more step-economical second-generation approach to tubuvaline, a series of *N*-acylhydrazone radical acceptors were required. From formyl thiazole **18**(53) (Scheme 4), treatment with allylzinc bromide afforded homoallylic alcohol **19** (racemic). Keck allylation^54^ was identified as a suitable enantioselective counterpart affording **19** with er 10:1, but most of the subsequent route was initially developed using racemic **19**. After O-benzylation to afford **17**, reduction of the ester gave primary alcohol **20**. Oxidative cleavage^55^ and subsequent condensation of the racemic aldehydes with enantiopure *N*-aminooxazolidinone **21** then furnished *N*-acylhydrazone **22** as a diastereomeric mixture. The primary alcohol was acylated to furnish the pivaloate derivative **23**. A variety of related hydrazone acceptors with various replacements of the Piv ester with other groups, such as silyl or benzyl ethers, were prepared in a similar fashion (see the Supporting Information). However, these were not as effective in subsequent radical additions.

When **23** (dr 1:1, Scheme 4) was subjected to Mn-mediated addition of isopropyl radical, there was exceptionally clean reactivity under the modified conditions (conditions B of Table 1). Although the reaction was incomplete, with 48% recovery of unreacted hydrazone (**23′**), it furnished 47% yield of isopropyl adduct **24** (90% yield based on conversion). Surprisingly, radical adduct **24** produced in this reaction appeared to be a single diastereomer; it gave only one set of signals in the ^13^C NMR spectrum, in contrast to the starting 1:1 11*R*/11*S* mixture **23** which had shown two sets of signals as expected.^56^ In attempting to secure complete conversion of *N*-acylhydrazone **23**, an unexpected phenomenon was observed; resubjection of recovered **23′** to the Mn-mediated radical addition did not provide any of the desired adduct **24**, and with longer reaction times, gradual decomposition was instead observed. Closer comparison of analytical data for original reactant **23** and recovered reactant **23′** showed that recovered **23′** had only one set of ^13^C NMR resonances. This contrasted with the two sets of ^13^C NMR peaks observed for reactant **23**, as expected for the C11 epimer mixture. Thus, both **24** and recovered **23′** were single diastereomers; one diastereomer of **23** had produced **24** via radical addition, while the other afforded no radical adduct.

### Elaboration to Tubuvaline

Conversion of radical adduct **24** to a protected form of tubuvaline (Scheme 4) entailed TFA installation and reductive N–N bond cleavage (SmI_2_), removal of the Piv ester (K_2_CO_3_, MeOH), and oxidation to the C-terminal carboxylate. The latter conversion involved telescoped oxidation and esterification, as the intermediate carboxylic acid suffered significant material loss upon attempted isolation. Thus, following oxidation of the primary alcohol by catalytic TEMPO in the presence of water, direct esterification by MeI gave the desired methyl ester. This sequence smoothly proceeded to afford *N*-TFA-*O*-benzyltubuvaline methyl ester (**10**) with spectroscopic properties that matched those of **10** from our previously published route.^48^ This confirmed the configurational assignment of (11*R*,13*R*)-**24**. Overall, the yield of **10** was 8.6% over 10 steps via route A (Scheme 3), a three-step improvement in step economy versus our prior approach.

### An Unexpected Kinetic Resolution Reveals 1,3-Diastereocontrol

The isolation of **23′** and **24** as single diastereomers indicates a kinetic resolution process via double diastereoselection; the 1:1 diastereomeric mixture of **23** would present matched and mismatched stereocontrol involving the two stereocontrol elements. The lower relative rate of the mismatched case could permit isolation of one diastereomeric product **24** and recovery of unreacted **23′**, also with diastereomeric enrichment. This kinetic resolution via radical addition is unprecedented, prompting further analysis.

**Figure 4:**
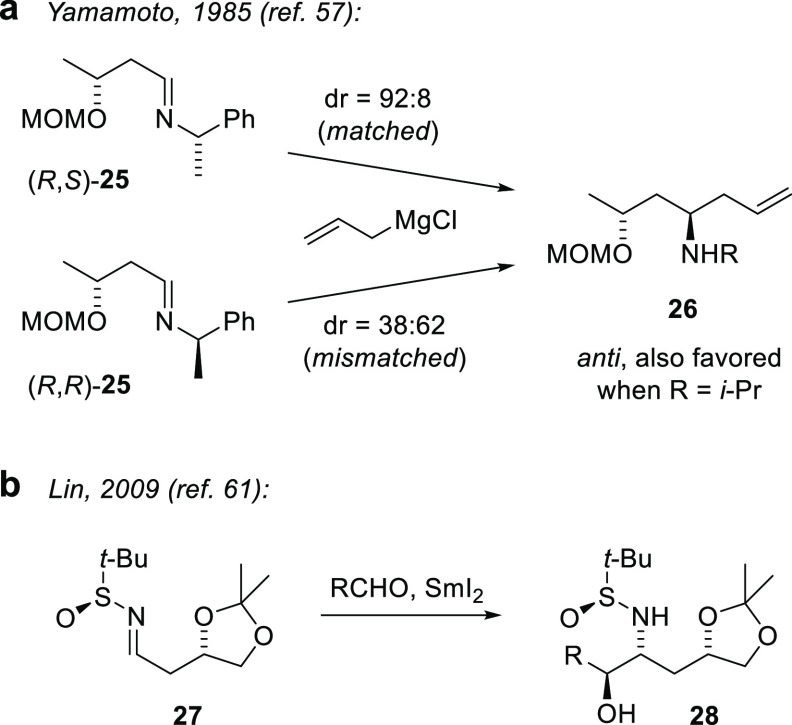
Examples of 1,3-diastereocontrol in additions to β-alkoxyimines. (a) Chelation-controlled antiselective Grignard addition with matched and mismatched α-phenethyl imines. (b) Pinacol-type radical addition to a β-alkoxysulfinimine.

For allylmetal addition to chiral β-alkoxyimines, the seminal studies of Yamamoto addressed double diastereoselection involving the β stereogenic center of **25** with alternate configurations of a chiral auxiliary at the imine nitrogen (Figure 4a).^57^ The chelated (allylMgCl) and nonchelated (allylborane) stereocontrol models discussed by Yamamoto have relevance to various other nonradical additions to β-substituted imino compounds.^58,59^ However, despite considerable literature searches, we have identified *no previously documented cases of double diastereoselection in intermolecular radical additions to β-alkoxyimino compounds*.^60^ In the closest precedent, Lin et al. reported a SmI_2_-mediated aza-pinacol coupling with β-alkoxysulfinimine **27** (Figure 4b) that incorporates two stereocontrol elements, but stereocontrol was attributed to the *N*-sulfinyl group and no comparison was made of matched and mismatched control.^61^ Similarly, we had observed complete stereocontrol in our Mn-mediated radical addition to β-alkoxyhydrazone **6**(47) (Scheme 1) and related compounds,^12^ but without examining matched/mismatched cases, we lacked a basis to expect strong contributions from 1,3-diastereocontrol.

Because these closest precedents were of questionable relevance to our radical addition, we explored our 1,3-diastereocontrol further. First, to determine the C11 configuration of **24**, *N*-acylhydrazone **23** was prepared with diastereomeric enrichment from a precursor of known configuration at C11. Asymmetric Keck allylation^54^ of formyl thiazole **18** in the presence of (*R*)-BINOL afforded (*R*)-**19** (er 10:1, Scheme 4) as judged by Mosher ester analysis, with a configuration consistent with the Keck precedent. Benzylation, carboxylate reduction, oxidative cleavage, and condensation with *N*-aminooxazolidinone afforded (11*R*)-**23** by the same sequence described in Scheme 4. The ^13^C NMR spectrum of this compound did not match that of **23′** recovered from incomplete reactions; instead, it matched the spectrum of the **23** diastereomer that was consumed during the radical addition. Thus, the recovered **23′** was assigned the 11*S* configuration, and the product **24** was assigned the 11*R* configuration.

**Scheme 5:**
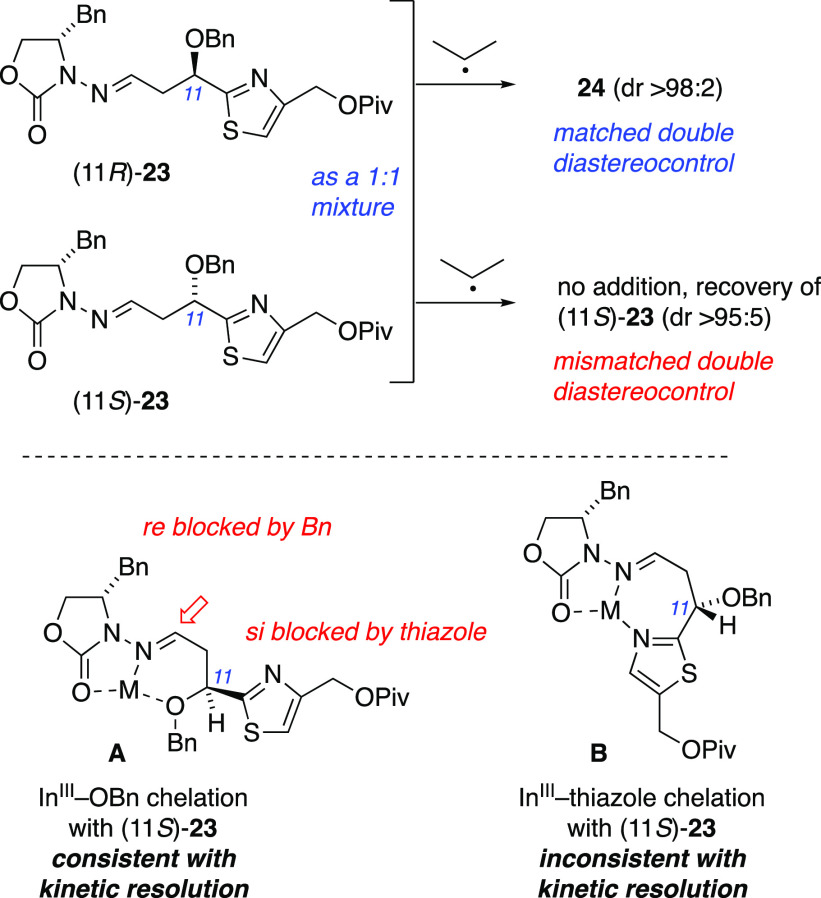


The configurational assignments of **23′** and **24** allow for structural hypotheses regarding modes of stereocontrol via chelated structures **A** and **B** (Scheme 5). In structure **A** that can reasonably be presumed to form upon mixing (11*S*)-**23** with InCl_3_,^62^ both *re* and *si* faces of the C=N acceptor carbon are blocked by the chiral *N*-acylhydrazone and the thiazole, respectively. This is consistent with poor reactivity due to mismatched double diastereocontrol. In alternative structure **B** with the thiazole coordinating to InCl_3_, the *si* face of the C=N in **B** appears to be unhindered; thus, we favor structure **A** to explain the mismatched double diastereocontrol with (11*S*)-**23**.

**Scheme 6:**
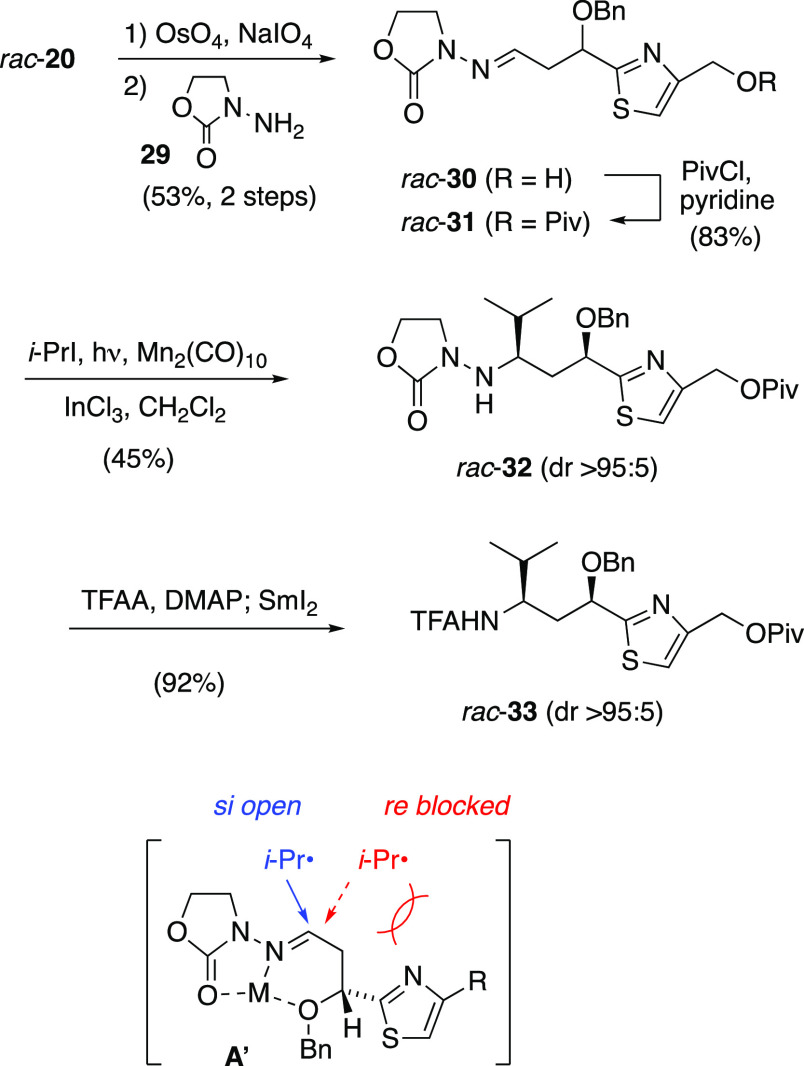


If the hypothetical structure **A** effectively blocks both faces as proposed, an analogous structure bearing the C11 stereogenic center as the only sterecontrol element would also be predicted to provide control of the relative configuration in the radical addition. A simple deletion of the *N*-acylhydrazone stereocontrol element tested this prediction. Racemic *N*-acylhydrazone **31** (Scheme 6) was prepared from alkene *rac*-**20** and achiral *N*-aminooxazolidinone **29** through oxidative cleavage, condensation, and esterification (i.e., the same sequence used to convert **20** to **23** in Scheme 4). Isopropyl radical addition, under the same Mn-mediated conditions of Scheme 4, gave *rac*-**32** with excellent diastereoselectivity, with dr >95:5 as judged by ^1^H NMR and ^13^C NMR spectra. Trifluoroacetylation and reductive cleavage of the N–N bond gave *rac*-**33 (**dr >95:5), with spectroscopic data matching the previous nonracemic sample obtained during conversion of **24** to **10** (Scheme 4). All these data confirm that auxiliary and substrate stereocontrol were mutually reinforcing in *N*-acylhydrazone (11*R*)-**23**, both favoring *si* face radical addition to the imino carbon, and that the β-alkoxy substituent is indeed a viable stereocontrol element for such radical additions. Thus, this route to Tuv yielded important new insights into 1,3-diastereocontrol and double differentiation in the context of the radical addition approach to asymmetric amine synthesis.

### Alternative Elaboration of the Tubuvaline Thiazole

**Scheme 7:**
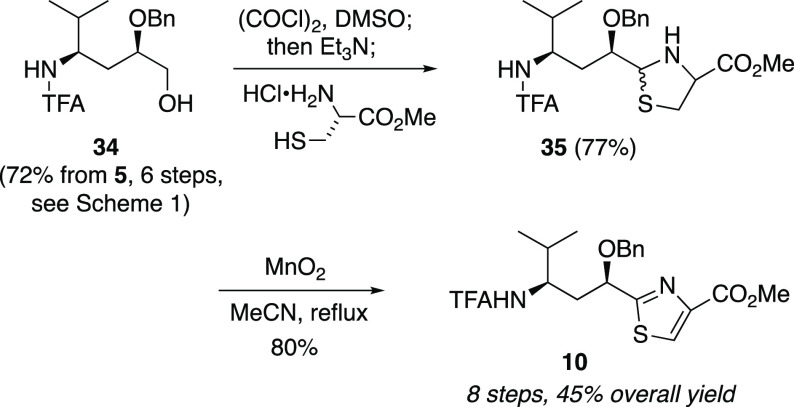


Although we had some success in our plan to improve the step efficiency by conducting radical addition in the presence of the thiazole (route A, Scheme 3), the overall chemical yield of 8.6% was less than desired. Our attention had also been drawn to a tactic from the tubulysin synthetic studies of Zanda^33^ and Chandrasekhar,^29^ in which tubuvaline assembly included cyclocondensation of an aldehyde with cysteine methyl ester followed by oxidation to forge the thiazole ring. We hypothesized that this thiazole construction could be applied as an alternative way to streamline our original preparation of the tubuvaline fragment. Thus, from alcohol **34**(48) (Scheme 7), Swern oxidation gave the corresponding aldehyde; cysteine methyl ester hydrochloride was directly added to the Swern oxidation mixture to furnish thiazoline **35** in a one-pot operation. Oxidation with manganese dioxide then completed route B to *N*-TFA-*O*-benzyltubuvaline methyl ester (**10**). This material gave spectral data matching that produced from route A as well as our earlier published route.^48^ Importantly, the efficiency as judged by both step count and overall yield were excellent, with 45% yield over eight steps from alcohol **5**.

### Modified Route to Tubulysins

**Scheme 8:**
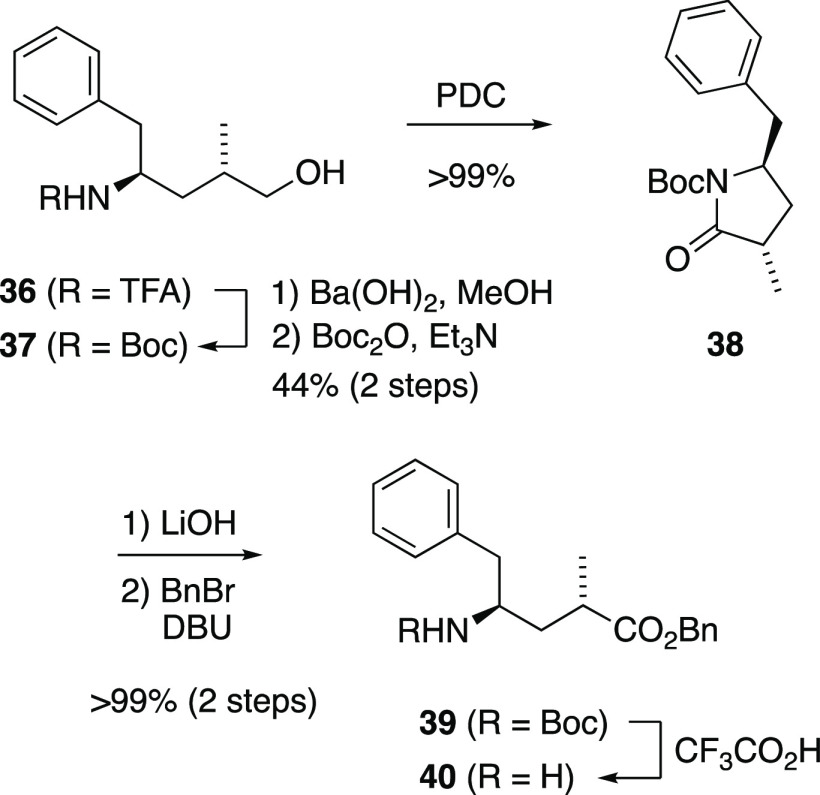


Our prior studies of tubulysin tetrapeptide assembly yielded a tetrapeptide C-terminal alcohol analog **11** (Figure 3),^48^ but oxidation to the corresponding carboxylic acid proved difficult. To avoid the late-stage oxidation, the tetrapeptide was assembled with the C-terminal amino acid Tup already in the carboxylate oxidation state. In hopes that an endgame debenzylation would deprotect both C11–OH and the C-terminal carboxylate concurrently, the Tup unit was introduced as a benzyl ester. This material was obtained from primary alcohol **36** (Scheme 8), available in stereochemically pure form via our previously published Mn-mediated radical addition route to Tup.^48^ Exchange of the *N*-TFA for *N*-Boc and oxidation with PDC proceeded via known compounds **37**(28) and **38**,^34^ and the known sequence of basic hydrolysis and benzyl esterification gave *N*-Boc-γ-amino ester **39**.^34^ Removal of the Boc group with trifluoroacetic acid furnished Tup ester **40** in the free amine form, suitable for peptide assembly.

**Scheme 9:**
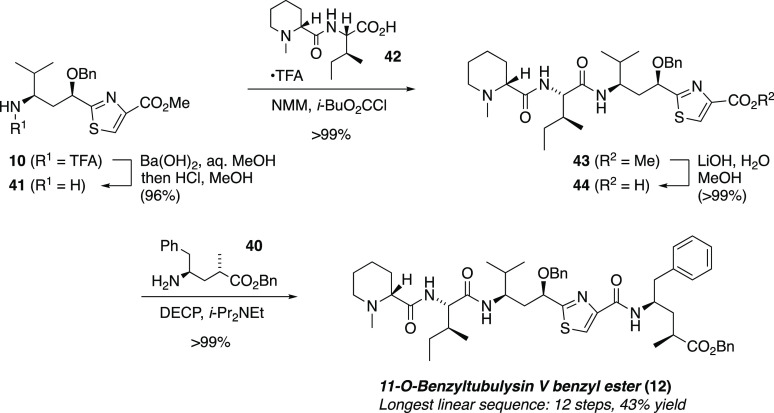


Our previously reported tetrapeptide assembly^48^ required modification of the sequence of couplings. Basic conditions hydrolyzed both *N*-TFA and methyl ester functions of Tuv derivative **10** (Scheme 9); the crude material was taken up in methanolic HCl to reinstall the methyl ester, providing amino ester **41**. Next, attachment of the Mep-Ile dipeptide **42** via its mixed anhydride gave tripeptide **43**. Ester saponification at the C-terminus and DECP-mediated coupling with Tup benzyl ester **40** then completed the tetrapeptide assembly to furnish **12** in excellent yield.

The final step en route to tubulysin V was envisioned to be a convenient hydrogenolysis of both benzyl groups. To our dismay, all efforts to hydrogenate the di-*O*-benzyl tetrapeptide **12** led to destruction of this material or no reaction at all.^63^ After considerable experimentation, a surprisingly effective alternative was finally identified: In a model study, a mixture of **10** and stannic chloride in boiling dichloromethane cleanly removed the benzyl ether to furnish **45** (eq 1).
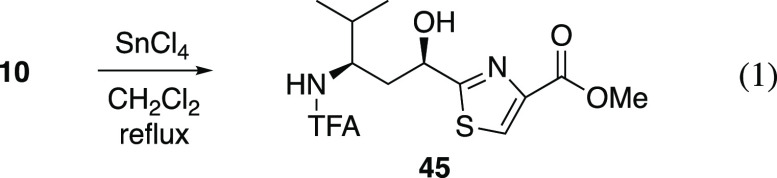
1

Exploratory experiments on debenzylation of the intact tetrapeptide **12** offer some evidence of its feasibility. Exposure of **12** to SnCl_4_ in refluxing CH_2_Cl_2_ indeed furnished a compound that had been twice debenzylated, as judged by high-resolution mass spectrometry (HRMS). The chemical behavior of this compound was consistent with the known reactivity of tubulysin V; it reacted with Ac_2_O and pyridine to give an *O*-acetyl derivative (tubulysin U) as judged by mass spectrometry. These results provide supporting evidence for the structure of 11-*O*-benzyltubulysin V benzyl ester (**12**), prepared in 43% overall yield via a 12-step longest linear sequence.

## 3 Conclusion

Building on prior data establishing Mn-mediated radical addition as an excellent means of stereocontrol for tubuphenylalanine (Tup) and tubuvaline (Tuv) synthesis, two alternative routes are presented toward the goal of improved efficiency in synthesis of Tuv. One of these routes required modification of Mn-mediated radical addition conditions to accommodate a thiazole in the radical acceptor, allowing broader versatility of the radical addition approach to asymmetric amine synthesis. While this route did not meet the efficiency goals, it uncovered a case of 1,3-diastereocontrol in radical addition to β-alkoxyimino compounds: in combination with the chiral *N*-acylhydrazone as a second stereocontrol element, a kinetic resolution occurred, separating two diastereomeric radical acceptors according to their differential reactivity in radical addition. The second route merged our radical addition chemistry with a rapid cyclocondensation and oxidation sequence to afford the thiazole of Tuv, with greater improvement to the overall efficiency, facilitating a high-yielding assembly of the 11-*O*-benzyltubulysin V benzyl ester.

## 4 Experimental Section

### Materials and Methods

Reactions employed oven- or flame-dried glassware under nitrogen unless otherwise noted. Toluene, tetrahydrofuran (THF), and CH_2_Cl_2_ were purchased inhibitor-free, deoxygenated by sparging with argon, and passed through columns of activated alumina under an argon atmosphere prior to use. Nitrogen was passed successively through columns of anhydrous CaSO_4_ and R3-11 catalyst for removal of water and oxygen, respectively. All other materials were used as received from commercial sources unless otherwise noted. Thin-layer chromatography (TLC) employed glass 0.25 mm silica gel plates with a UV indicator. Flash chromatography columns were packed with 230–400 mesh silica gel as a slurry in the initial elution solvent. Gradient flash chromatography was conducted by adsorption of product mixtures on silica gel, packing over a short pad of clean silica gel as a slurry in hexane, and eluting with a continuous gradient as indicated. Radial chromatography refers to centrifugally accelerated thin-layer chromatography performed using commercially supplied rotors. Nuclear magnetic resonance (NMR) data were obtained at operating frequencies indicated in the text and are reported in units of ppm. Infrared spectra were recorded using a single beam FT-IR spectrophotometer by standard transmission method. Low- and high-resolution mass spectra (TOF) were obtained from local instrumentation facilities services.

#### Conditions for Compatibility of *N*-Heteroaromatics with Mn-Mediated Radical Addition

##### General Procedure A (Table 1, Footnote a)

A Schlenk flask charged with *N*-acylhydrazone **15** (1 equiv), *N*-heteroaromatic additive (1 equiv), indium chloride (2.2 equiv), and CH_2_Cl_2_ (0.11 M) was stirred for 30 min. Isopropyl iodide (10 equiv) was added via syringe, followed by dimanganese decacarbonyl (1 equiv). The Schlenk flask was sealed and irradiated (300 nm, Rayonet photoreactor) for 6 h. Concentration and flash chromatography or gradient flash chromatography furnished the *N*-acylhydrazine **16**. Preparation and characterization of hydrazine **16** have been previously reported.^5^

##### General Procedure B (Table 1, Footnote b)

A Schlenk flask charged with *N*-acylhydrazone **15** (1 equiv), additive (1 equiv), indium chloride (3.5 equiv), and CH_2_Cl_2_ (0.017 M) was stirred for 30 min. Isopropyl iodide (3 equiv) was added via syringe, followed by dimanganese decacarbonyl (1 equiv). The Schlenk flask was sealed and irradiated (broad spectrum with maximum at 300 nm, Rayonet photoreactor)^64^ for 6 h. Concentration and flash chromatography or gradient flash chromatography furnished the *N*-acylhydrazine **16**.^5^

#### Preparation and Characterization Data for New Compounds.^65^

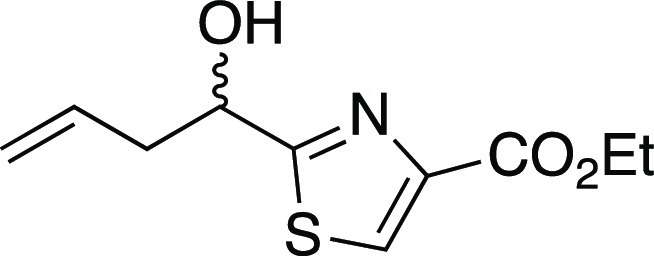



##### Ethyl 2-(1-Hydroxybut-3-enyl)thiazole-4-carboxylate (*rac*-**19**)

To a mixture of ZnCl_2_ (5.90 g, 43.3 mmol) and THF (120 mL) was added allylmagnesium chloride (2 M in THF, 19.7 mL, 39.4 mmol) slowly via syringe. After being stirred for 0.5 h, this mixture was transferred via cannula into a solution of formyl thiazole **18**(53) (7.20 g, 39.4 mmol) in THF (150 mL). After 3 h, the reaction was quenched with water (20 mL) and extracted with EtOAc. The organic phase was dried over Na_2_SO_4_. Concentration and gradient flash chromatography (25% EtOAc in petroleum ether to 50% EtOAc in petroleum ether) afforded homoallylic alcohol *rac*-**19** (5.27 g, 60% yield) as a pale yellow solid: mp 46.0–46.5 °C; IR (film) 3411, 2983, 1725, 1489, 1215 cm^–1^; ^1^H NMR (300 MHz, CDCl_3_) δ 8.13 (s, 1H), 5.90–5.76 (m, 1H), 5.27–5.20 (m, 2H), 5.11 (dd, *J* = 7.9, 4.0 Hz, 1H), 4.42 (q, *J* = 7.1 Hz, 2 H), 2.91–2.82 (m, 1H), 2.79 (br s, 1H), 2.66–2.55 (m, 1H), 1.40 (t, *J* = 7.2 Hz, 3H); ^13^C{^1^H} NMR (75 MHz, CDCl_3_) δ 175.9, 161.5, 146.9, 132.8, 127.5, 119.7, 71.0, 61.6, 42.4, 14.4; HRMS (ESI) *m*/*z* [M + H]^+^ calcd for C_10_H_14_NO_3_S 228.0694; found 228.0686.
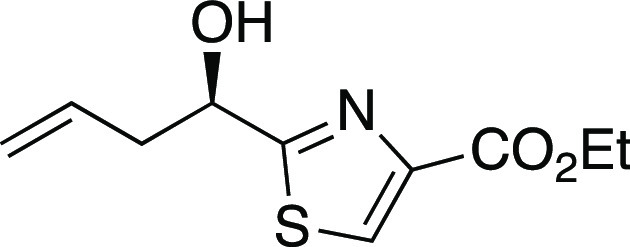


##### Ethyl 2-(1*R*-Hydroxybut-3-enyl)thiazole-4-carboxylate ((*R*)-**19**)

To activated 4 Å molecular sieves (23 mg) were added (*R*)-BINOL (6.2 mg, 0.022 mmol). CH_2_Cl_2_ (0.6 mL) and Ti(O-*i*-Pr)_4_ (12 μL, 0.040 mmol) were added via syringe. After 1 h, formyl thiazole **18**(53) (20 mg, 0.11 mmol) was added. After cooling to −78 °C, allyltributylstannane (0.10 mL, 0.32 mmol) was added via syringe. The mixture was stirred for 10 min at −78 °C and then stored in a freezer (ca. −20 °C) for 24 h. The mixture was partitioned between saturated aqueous sodium bicarbonate solution (2 mL) and EtOAc, and the organic phase was dried over Na_2_SO_4_. Concentration and flash chromatography (17% EtOAc in hexane to 33% EtOAc in hexane to 50% EtOAc in hexane) afforded alcohol (*R*)-**19** (22.7 mg, 93% yield) as a pale yellow solid: [α]_D_^20.6^ = +70.6 (*c* 1.14, CHCl_3_).
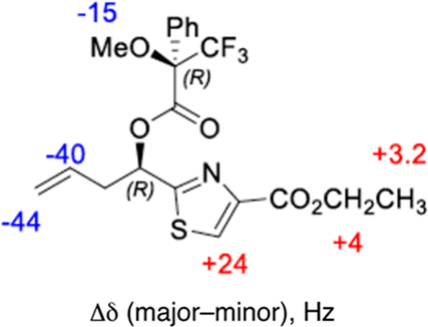


##### Configuration Assignment: Mosher Ester of (R)-**19** (**S1**)

To a solution of (*R*)-**19** (1 mg, 4.4 μmol) in CH_2_Cl_2_ (0.25 mL) was added pyridine (1 μL, 0.0124 mmol) via syringe. (*S*)-Mosher’s acid chloride (5 mg, 0.020 mmol) was added. After 24 h, reaction mixture was concentrated. Flash chromatography afforded Mosher’s ester **S1** (1.6 mg, 82% yield) as a colorless oil: ^1^H NMR (400 MHz, CDCl_3_) δ 8.14 (s, 1H), 8.09 (s, minor diastereomer peak), 7.58–7.51 (m, 2H), 7.45–7.36 (m, 3H), 6.43 (dd, *J* = 7.5, 4.7 Hz, 1H), 5.80- 5.70 (m, minor diastereomer peak), 5.70–5.60 (m, 1H), 5.19–5.11 (m, minor diastereomer peak), 5.08–5.00 (m, 2H), 4.43 (dd, *J* = 14.3, 7.0 Hz, 2H), 3.58 (s, minor diastereomer peak), 3.54 (s, 3H), 2.95–2.76 (m, 2H), 1.40 (t, minor diastereomer peak), 1.41 (t, *J* = 7.1 Hz, 3H).
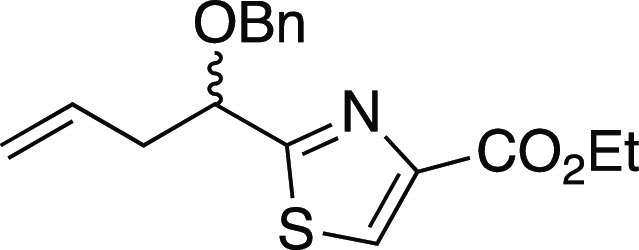


##### Ethyl 2-(1-(Benzyloxy)but-3-enyl)thiazole-4-carboxylate) (**S2**)

To a solution of homoallylic alcohol *rac*-**19** (1.16 g, 5.12 mmol) in THF (100 mL) was added KH (30 wt % in mineral oil, 821 mg, 6.14 mmol) in one portion. After 5 min, benzyl bromide (0.73 mL, 6.14 mmol) was added via syringe. After 4 h, the reaction mixture was quenched with water (30 mL), concentrated to remove THF, and extracted with EtOAc. The organic phase was dried over Na_2_SO_4_. Concentration and flash chromatography (9% EtOAc in petroleum ether) afforded benzyl ether **S2** (884 mg, 54% yield) as a pale yellow oil: IR (film) 3015, 2910, 1725, 1487, 1315, 1217, 1096 cm^–1^; ^1^H NMR (300 MHz, CDCl_3_) δ 8.17 (s, 1H), 7.36–7.28 (m, 5H), 5.90–5.77 (m, 1H), 5.15–5.05 (m, 2H), 4.91 (dd, *J* = 7.0, 5.4 Hz, 1H), 4.58 (ABq, *Δν* = 23.9 Hz, *J* = 11.6 Hz, 2H), 4.44 (q, *J* = 7.1 Hz, 2H), 2.74–2.63 (m, 2H), 1.41 (t, *J* = 7.1 Hz, 3H); ^13^C{^1^H} NMR (75 MHz, CDCl_3_) δ 174.8, 161.5, 147.1, 137.4, 133.0, 128.6, 128.1, 128.0 (2C), 118.5, 78.8, 72.3, 61.6, 41.4, 14.5; HRMS (ESI) *m*/*z* [M + H]^+^ calcd for C_17_H_20_NO_3_S 318.1164, found 318.1154.
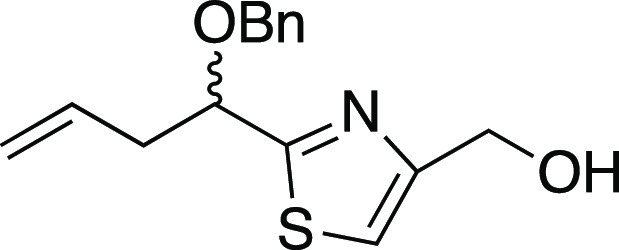


##### (2-(1-(Benzyloxy)but-3-enyl)thiazol-4-yl)methanol (*rac*-**20**)

A solution of ester **S2** (1.56 g, 4.91 mmol) in THF (98 mL) was cooled to −78 °C, and lithium aluminum hydride (2.0 M in THF, 4.91 mL, 9.82 mmol) was added slowly via syringe. After 1 h, the reaction was warmed to room temperature, quenched by slow addition of water (50 mL), and extracted with EtOAc. The organic phase was dried over Na_2_SO_4_ and concentrated. Flash chromatography (67% EtOAc in petroleum ether) afforded alcohol *rac*-**20** (1.26 g, 93% yield) as a pale yellow oil: IR (film) 3339, 3078, 3031, 2978, 2869, 1642, 1454, 1324, 1075 cm^–1^; ^1^H NMR (300 MHz, CDCl_3_) δ 7.40–7.25 (m, 5H), 7.20 (s, 1H), 5.93–5.74 (m, 1H), 5.19–5.02 (m, 2H), 4.83–4.74 (m, 3H), 4.58 (ABq, *Δν* = 36.5 Hz, *J* = 11.6 Hz, 2H), 3.36 (br s, 1H), 2.80–2.57 (m, 2H); ^13^C{^1^H} NMR (75 MHz, CDCl_3_) δ 174.2, 156.2, 137.6, 133.2, 128.5, 128.0 (2C), 118.2, 115.3, 78.7, 72.0, 60.8, 41.4; HRMS (ESI) *m*/*z* [M + H]^+^ calcd for C_15_H_18_NO_2_S 276.1058; found 276.1053.
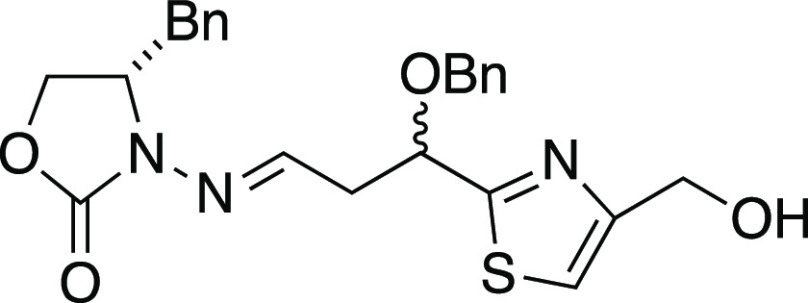


##### (*S*)-3-(3-(Benzyloxy)-3-(4-(hydroxymethyl)thiazol-2-yl)propylideneamino)-4-benzyloxazolidin-2-one (**22**)

To a solution of alkene *rac*-**20** (86 mg, 0.31 mmol) in THF (21 mL) and water (21 mL) was added osmium tetraoxide (2.5 wt % in *tert*-butyl alcohol, 0.12 mL, 0.012 mmol). After 5 min, sodium periodate (265 mg, 1.24 mmol) was added. After 5.5 h, the reaction was quenched with saturated aqueous sodium thiosulfate solution (50 mL) and extracted with EtOAc. The organic phase was dried over Na_2_SO_4_, concentrated, then filtered through a short column of silica gel, eluting with EtOAc. Concentration furnished the crude aldehyde, which was dissolved in CH_2_Cl_2_ (31 mL) along with (*S*)-3-amino-4-phenylmethyl-2-oxazolidinone (**21**, 119 mg, 0.62 mmol). After 12.5 h, concentration and gradient flash chromatography (83% EtOAc in petroleum ether to EtOAc to 5% methanol in EtOAc) afforded **22** (106 mg, 75% yield, inseparable 1:1 mixture of diastereomers) as a colorless wax: IR (film) 3404, 3013, 1763, 1405, 1217, 1092 cm^–1^; ^1^H NMR (300 MHz, CDCl_3_) δ 7.91–7.84 (m, 1H), 7.38–7.19 (m, 9H), 7.09–7.04 (m, 2H), 5.08–5.02 (m, 1H), 4.75–4.52 (m, 4H), 4.33–4.23 (m, 1H), 4.20–4.15 (m, 1H), 4.10–4.03 (m, 1H), 3.73 (br s, 1H), 3.15–2.93 (m, 3H), 2.77–2.63 (m, 1H), (diastereomer peaks were unresolved); ^13^C{^1^H} NMR (75 MHz, CDCl_3_) δ 172.5, 156.8, 154.0, 149.6 and 149.4, 137.0, 134.9, 129.2, 128.8, 128.43 and 128.42, 127.9 (2C), 127.2, 115.4, 76.5 and 76.4, 71.9 and 71.8, 65.6, 60.6, 56.4, 40.04 and 39.97, 36.1, and 36.0 (some diastereomer peaks were unresolved); HRMS (ESI) *m*/*z* [M + Na]^+^ calcd for C_24_H_25_N_3_O_4_SNa 474.1463; found 474.1451.
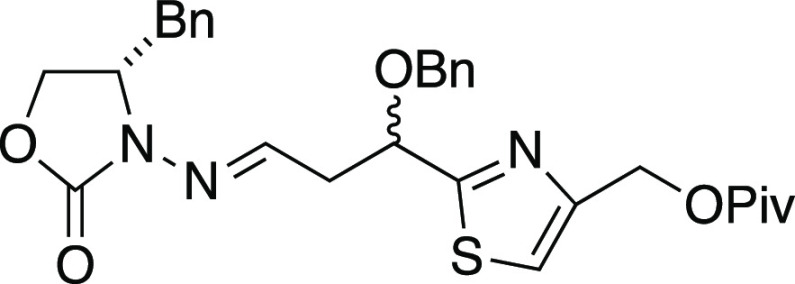


##### (*S*)-3-(3-(Benzyloxy)-3-(4-((pivaloyloxymethyl)thiazol-2-yl)propylideneamino)-4-benzyloxazolidin-2-one (**23**)

To a solution of alcohol **22** (220 mg, 0.487 mmol) and pyridine (0.20 mL, 2.4 mmol) in CH_2_Cl_2_ (4.9 mL) was added pivaloyl chloride (0.09 mL, 0.7 mmol) via syringe. After 16 h, concentration and flash chromatography (50% EtOAc in petroleum ether) afforded pivaloate **23** (209 mg, 80% yield, inseparable 1:1 mixture of diastereomers) as a colorless oil: IR (film) 3019, 2980, 2939, 1742, 1208, 1144, 1047 cm^–1^; ^1^H NMR (300 MHz, CDCl_3_) δ 8.02–7.95 (m, 1H), 7.38–7.23 (m, 9H), 7.14–7.04 (m, 2H), 5.22–5.19 (m, 2H), 5.09–5.04 (m, 1H), 4.74–4.56 (m, 2H), 4.37–4.18 (m, 2H), 4.14–4.08 (m, 1H), 3.20–3.08 (m, 1H), 3.08–3.03 (m, 2H), 2.80–2.65 (m, 1H), 1.23 (s, 9H) (diastereomer peaks were unresolved); ^13^C{^1^H} NMR (75 MHz, CDCl_3_) δ 178.1, 172.53 and 172.48, 154.1, 152.0, 150.0 and 149.8, 137.2, 135.1, 129.4, 129.0, 128.6, 128.1 (2C), 127.4, 117.21 and 117.17, 76.6 and 76.5, 72.1 and 72.0, 65.7, 61.9, 57.0, 40.24 and 40.16, 38.9, 36.5 and 36.4, 27.2 (some diastereomer peaks were unresolved); HRMS (ESI) *m*/*z* [M + H]^+^ calcd for C_29_H_34_N_3_O_5_S 536.2219; found 536.2208.
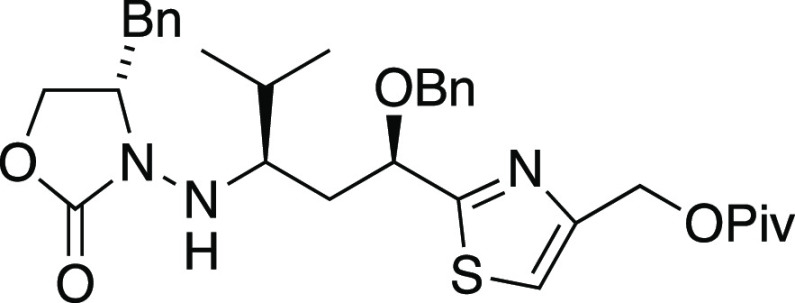


##### (1*S*,1′*R*,3′*R*)-3-(1′-(Benzyloxy)-1′-(4″-(pivaloyloxymethyl)thiazol-2″-yl)-4′-methylpentan-3′-ylamino)-4-benzyloxazolidin-2-one (**24**)

From *N*-acylhydrazone **23** (104 mg, 0.194 mmol, 1:1 mixture of diastereomers) via general procedure B was obtained amine **24** (53 mg, 47% yield) as a colorless oil: IR (film) 3290, 2963, 2872, 1755, 1604, 1497, 1398, 1368, 1281, 1216, 1160, 1095 cm^–1^; ^1^H NMR (300 MHz, CDCl_3_) δ 7.40–7.22 (m, 9H), 7.12–7.05 (m, 2H), 5.22 (s, 2H), 5.08 (dd, *J* = 7.7, 3.9 Hz, 1H), 4.64 (ABq, *Δν* = 36.2 Hz, *J* = 11.4 Hz, 2H), 4.35 (br s, 1H), 4.05 (dd, apparent t, *J* = 8.2 Hz, 1H), 3.95 (dd, *J* = 8.8, 4.5 Hz, 1H), 3.91–3.80 (m, 1H), 3.21 (dd, *J* = 13.4, 3.4 Hz, 1H), 3.17–3.08 (m, 1H), 2.49 (dd, *J* = 13.4, 9.9 Hz, 1H), 2.14–1.78 (m, 3H), 1.24 (s, 9H), 0.94 (d, *J* = 6.8 Hz, 3H), 0.88 (d, *J* = 6.9 Hz, 3H); ^13^C{^1^H} NMR (75 MHz, CDCl_3_) δ 178.1, 174.2, 158.5, 151.8, 137.3, 135.7, 129.1, 128.8, 128.5, 128.0, 127.9, 127.0, 116.7, 77.6, 72.4, 65.5, 61.9, 59.8, 57.8, 38.8, 36.4, 35.8, 28.7, 27.2, 18.9, 15.9; HRMS (ESI) *m*/*z* [M + H]^+^ calcd for C_32_H_42_N_3_O_5_S 580.2845; found 580.2840.
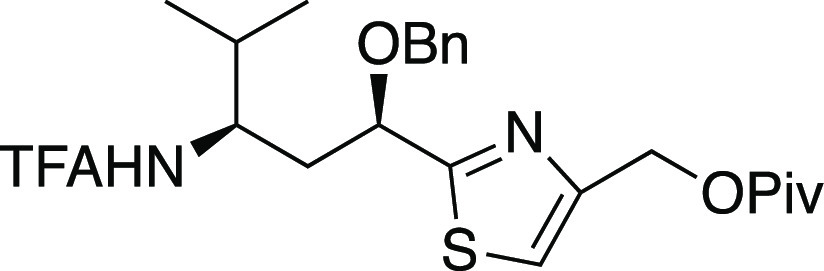


##### (2-((1′*R*,3′*R*)-3′-(Trifluoroacetamido)-1′-(benzyloxy)-4′-methylpentyl)thiazol-4-yl)methyl pivaloate (**33**), from Chiral Oxazolidinone Precursor **24**

To the solution of amine **24** (31 mg, 0.053 mmol) and DMAP (130 mg, 1.06 mmol) in CH_2_Cl_2_ (0.5 mL) was added TFAA (0.5 mL). After 15 h, mixture was filtered and concentrated. Flash chromatography (25% EtOAc in hexane) afforded intermediate (34 mg, 94% yield) as colorless oil. Part of this intermediate (6 mg, 0.0089 mmol) was dissolved in THF (0.045 mL) and MeOH (0.045 mL, dried over activated 4 Å molecular sieves), to which samarium iodide solution (0.2 M in THF) was added until the blue color persisted for 1 s. After quenching by exposure to air, concentration and flash chromatography (25% EtOAc in hexane) afforded TFA protected amine **33** (3.3 mg, 70% yield over two steps) as a colorless oil. IR (film) 3336, 3020, 2963, 2927, 2853, 1720, 1457 cm^–1^; ^1^H NMR (400 MHz, CDCl_3_) δ 7.39–7.30 (m, 5H), 7.25 (s, 1H), 6.88 (d, *J* = 9.2 Hz, 1H), 5.21 (s, 2H), 4.81 (dd, *J* = 8.9, 4.3 Hz, 1H), 4.55 (ABq, *Δν* = 58.4 Hz, *J* = 10.7 Hz, 2H), 4.17–4.07 (m, 1H), 2.15–1.99 (m, 2H), 1.83–1.73 (m, 1H), 1.25 (s, 9H), 0.94 (d, *J* = 6.8 Hz, 3H), 0.90 (d, *J* = 6.8 Hz, 3H); ^13^C{^1^H} NMR (100 MHz, CDCl_3_) δ 178.2, 173.8, 157.2 (q, *J* = 36 Hz), 152.0, 136.8, 128.8, 128.7, 128.5, 117.2, 116.1 (t, *J* = 288 Hz), 76.5, 72.9, 61.9, 52.7, 39.0, 38.9, 31.7, 27.3, 19.0, 18.4; HRMS (ESI) *m*/*z* [M + H]^+^ calcd for C_24_H_32_F_3_N_2_O_4_S 501.2035; found 501.2031.

##### (2-((1′R*,3′R*)-3-(2,2,2-Trifluoroacetamido)-1-(benzyloxy)-4-methylpentyl)thiazol-4-yl)methyl Pivalate (*rac*-**33**), from Achiral Oxazolidinone Precursor *rac*-**32**

To the solution of amine *rac-***32** (10 mg, 0.020 mmol) and DMAP (49 mg, 0.4 mmol) in CH_2_Cl_2_ (0.2 mL) was added TFAA (0.2 mL). After 27 h, the mixture was filtered and concentrated. Flash chromatography (17% EtOAc in hexane) afforded a colorless oil: HRMS (ESI) *m*/*z* [M + H]^+^ calcd for C_27_H_35_F_3_N_3_O_6_S 586.2199; found 586.2185. To a solution of this material in THF (0.05 mL) and MeOH (0.05 mL, dried over activated 4 Å molecular sieves) was added samarium iodide solution (0.2 M) until the blue color remained for 1 s. After quenching by exposure to air, concentration and flash chromatography (17% EtOAc in hexane) afforded TFA protected amine *rac*-**33** (9.4 mg, 92% yield over two steps) as colorless oil.
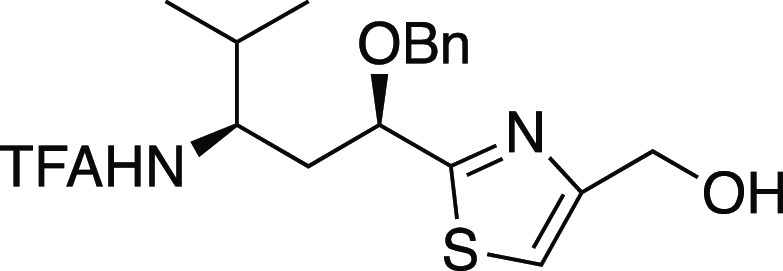


##### (2-((1′*R*,3′*R*)-3′-(Trifluoroacetamido)-1′-(benzyloxy)-4′-methylpentyl)thiazol-4-yl)methanol (**S3**)

To a solution of pivaloate **33** (9.0 mg, 0.018 mmol**)** in MeOH (1.8 mL) was added potassium carbonate (5.0 mg, 0.036 mmol). After 16 h, saturated aqueous ammonium chloride solution (0.5 mL) was added. This mixture was extracted with EtOAc, and the organic layer was dried over Na_2_SO_4_. Concentration and gradient flash chromatography (17% EtOAc in hexane to 67% EtOAc in hexane) afforded 24% recovery of reactant **33** and alcohol **S3** (5.7 mg, 76% yield) as a colorless oil: [α]_D_^20^ + 22.4 (*c* 0.285, CHCl_3_); IR (film) 3279, 3018, 2966, 2400, 1720, 1215 cm^–1^; ^1^H NMR (400 MHz, CDCl_3_) δ 7.40–7.31 (m, 5H), 7.22 (s, 1H), 6.72 (d, *J* = 8.8 Hz, 1H), 4.81 (dd, *J* = 9.3, 3.7 Hz, 1H), 4.77 (s, 2H), 4.55 (ABq, *Δν* = 64.5 Hz, *J* = 10.7 Hz, 2H), 4.18–4.07 (m, 1H), 2.63 (br s, 1H), 2.15–1.96 (m, 2H), 1.83–1.72 (m, 1H), 0.93 (d, *J* = 6.8 Hz, 3H), 0.89 (d, *J* = 6.8 Hz, 3H); ^13^C{^1^H} NMR (100 MHz, CDCl_3_) δ 174.1, 157.2 (q, *J* = 36 Hz), 156.2, 136.7, 128.6, 128.5, 128.4, 116.2 (q, *J* = 290 Hz), 115.4, 76.4, 72.9, 60.9, 52.5, 38.9, 31.7, 18.8, 18.3; HRMS (ESI) *m*/*z* [M + Na]^+^ calcd for C_19_H_23_F_3_N_2_O_3_SNa 439.1279; found 439.1270.
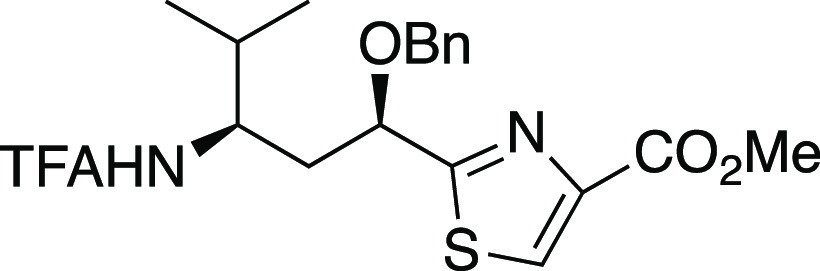


##### Methyl (2-((1′R,3′R)-1′-Benzyloxy-4′-methyl-3′-(trifluoroacetamido)pent-1′-yl)-thiazol-4-yl)carboxylate (N-TFA-11-O-benzyltubuvaline methyl ester, **10**). Procedure via oxidation of alcohol **S3**

A solution of TEMPO (0.7 mg, 5 μmol), BAIB (30 mg, 0.092 mmol) and CH_2_Cl_2_ (0.6 mL) was prepared. A portion (ca. 10%) of this solution was added into alcohol **S3** (1.9 mg, 0.0046 mmol), then water (0.012 mL) was added. After 30 min, an additional portion of TEMPO (0.3 mg, 2 μmol) was added. After 20 h, the reaction mixture was partitioned between diethyl ether and saturated sodium bicarbonate aqueous solution. The aqueous phase was acidified with 2 M HCl, then extracted with EtOAc. The organic phase was dried over Na_2_SO_4_ and concentrated to afford the crude carboxylic acid as colorless oil. To a solution of this carboxylic acid in DMF (0.2 mL) was added potassium carbonate (0.7 mg, 5.2 μmol) and iodomethane (0.6 mg, 3.9 μmol; measured via syringe weight before and after addition). After 21 h, flash chromatography (25% EtOAc in hexane) afforded methyl ester **10** as colorless oil (1.3 mg, 64% yield).

##### Procedure via oxidation of thiazolidine **35**

To a solution of thiazolidine **35** (10 mg, 0.02 mmol) in anhydrous acetonitrile was added MnO_2_ (20 mg, 0.2 mmol). After 9 h, another portion of MnO_2_ (20 mg, 0.2 mmol) was added. After 12 h, the reaction mixture was filtered through Celite and concentrated. Flash chromatography (2:1 petroleum ether/ethyl acetate) afforded **10** (8 mg, 80% yield) as a pale yellow oil: [α]_D_^23^ +12.0 (*c* 0.26, CHCl_3_); IR (film) 3583, 3315, 2962, 2924, 1722, 1710, 1207, 1180; ^1^H NMR (400 MHz, CDCl_3_) δ 8.21 (s, 1H), 7.48–7.29 (m, 5H), 6.63 (d, *J* = 9.7 Hz, 1H), 4.89 (dd, *J* = 9.7, 3.8 Hz, 1H), 4.54 (ABq, *J* = 10.6 Hz, *Δν* = 35 Hz, 2H), 4.29–4.12 (m, 1H), 3.97 (s, 3H), 2.12–1.98 (m, 2H), 1.78 (dq, *J* = 13.4, 6.7 Hz, 1H), 0.94 (d, *J* = 6.8 Hz, 3H), 0.90 (d, *J* = 6.8 Hz, 3H); ^13^C{^1^H} NMR (100 MHz, CDCl_3_) 174.8, 161.6, 157.0 (q, *J* = 36 Hz), 146.9, 136.4, 128.6, 128.6, 128.4, 128.2, 115.9 (q, *J* = 288 Hz), 76.3, 73.2, 52.6, 52.1, 39.2, 31.8, 18.9, 18.0; MS (ESI) *m*/*z* (rel intensity) 467 ([M + Na]^+^, 100), 445 [M + H]^+^, 26); HRMS (ESI) *m*/*z* [M + Na]^+^ calcd for C_20_H_23_F_3_N_2_O_4_SNa 467.1228; found: 467.1224.
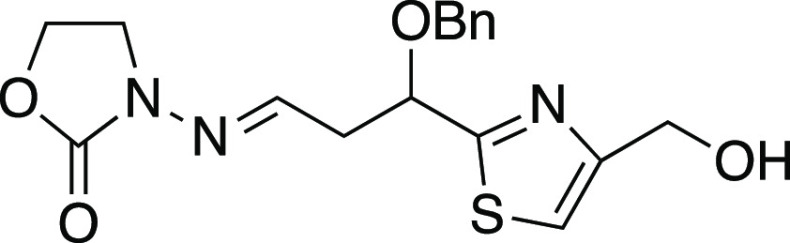


##### 3-(3-(Benzyloxy)-3-(4-(hydroxymethyl)thiazol-2-yl)propylideneamino)oxazolidin-2-one (*rac*-**30**)

To a solution of alkene *rac-***20** (170 mg, 0.62 mmol) in THF (31 mL) and water (31 mL) was added osmium tetraoxide (2.5 wt % in *tert*-butyl alcohol, 0.06 mL, 0.006 mmol). After 5 min, sodium periodate (535 mg, 2.5 mmol) was added. After 13 h, the reaction was quenched with saturated aqueous sodium thiosulfate solution (100 mL) and extracted with EtOAc. The organic phase was dried over Na_2_SO_4_, concentrated, and filtered through a short column of silica gel, eluting with EtOAc. Concentration furnished the crude aldehyde (180 mg), part of which (150 mg) was dissolved in CH_2_Cl_2_ (50 mL) along with 3-amino-2-oxazolidinone (120 mg, 1.18 mmol). After 12 h, concentration and flash chromatography (5% MeOH in CH_2_Cl_2_) afforded hydrazone *rac*-**30** (98 mg, 53% yield over two steps, racemic) as a colorless wax: IR (film) 3400, 3018, 2252, 1772, 1407, 1215, 1091 cm^–1^; ^1^H NMR (600 MHz, CDCl_3_) δ 7.37–7.27 (m, 5H), 7.22 (s, 1H), 6.94 (t, *J* = 5.5 Hz, 1H), 4.98 (dd, *J* = 6.8, 5.5 Hz, 1H), 4.74 (s, 2H), 4.61 (ABq, *Δν* = 85.0 Hz, *J* = 11.7 Hz, 2H), 4.44 (dd, apparent t, *J* = 8.0 Hz, 2H), 3.69–3.62 (m, 2H), 3.28 (br s, 1H), 3.06–2.95 (m, 2H); ^13^C{^1^H} NMR (150 MHz, CDCl_3_) δ 172.5, 156.7, 154.6, 143.6, 137.2, 128.6, 128.19, 128.16, 115.5, 76.6, 72.0, 61.5, 60.8, 42.1, 39.4; HRMS (ESI) *m*/*z* [M + H]^+^ calcd for C_17_H_20_N_3_O_4_S 362.1174; found 362.1167.
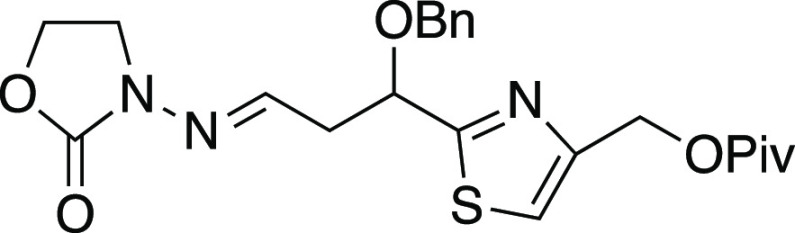


##### 3-(3-(Benzyloxy)-3-(4-((pivaloyloxymethyl)thiazol-2-yl)propylideneamino)oxazolidin-2-one (*rac*-**31**)

To a solution of alcohol *rac*-**30** (88 mg, 0.24 mmol) in CH_2_Cl_2_ (2.4 mL) was added pivaloyl chloride (44 μL, 0.36 mmol) and pyridine (58 μL, 0.72 mmol) via syringe. After 7 h, gradient flash chromatography (9% EtOAc in hexane to 17% EtOAc in petroleum ether) afforded pivaloate *rac*-**31** (90 mg, 83% yield) as a colorless oil: IR (film) 3017, 2976, 1772, 1728, 1479, 1407, 1216, 1152 cm^–1^; ^1^H NMR (600 MHz, CDCl_3_) δ 7.38–7.29 (m, 5H), 7.24 (s, 1H), 6.97 (dd, apparent t, *J* = 5.5 Hz, 1H), 5.21 (s, 2H), 4.98 (dd, *J* = 6.8, 5.7 Hz, 1H), 4.62 (ABq, Δν = 84.2 Hz, *J* = 11.7 Hz, 2H), 4.47 (t, *J* = 7.9 Hz, 2 H), 3.71–3.64 (m, 2H), 3.09–2.97 (m, 2H), 1.24 (s, 9H); ^13^C{^1^H} NMR (150 MHz, CDCl_3_) δ 178.2, 172.3, 154.5, 152.1, 143.7, 137.3, 128.7, 128.3, 128.2, 117.3, 76.6, 72.0, 61.9, 61.4, 42.2, 39.4, 39.0, 27.3; HRMS (ESI) *m*/*z* [M + H]^+^ calcd for C_22_H_28_N_3_O_5_S 446.1750; found 446.1743.
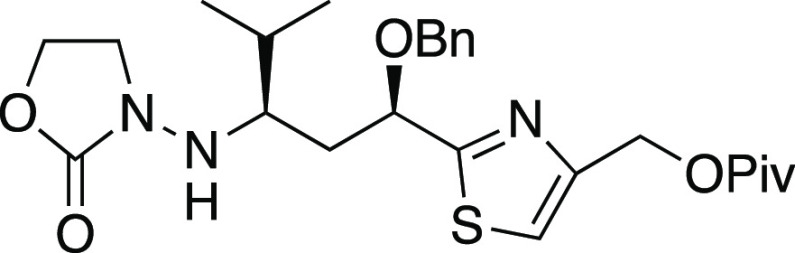


##### (1′R*,3′R*)-3-(1′-(Benzyloxy)-1′-(4″-(pivaloyloxymethyl)thiazol-2″-yl)-4′-methylpentan-3′-ylamino)oxazolidin-2-one (rac-**32**)

From *N*-acylhydrazone *rac*-**31** (49 mg, 0.11 mmol) via general procedure B was obtained amine *rac*-**31** (24 mg, 45% yield) as a colorless oil: IR (film) 3440, 3293, 2960, 2872, 1731, 1479, 4555, 1397, 1368, 1281, 1150 cm^–1^; ^1^H NMR (600 MHz, CDCl_3_) δ 7.40–7.25 (m, 5H), 7.22 (s, 1H), 5.21 (s, 2H), 5.01 (dd, *J* = 8.2, 3.6 Hz, 1H), 4.62 (ABq, *Δν* = 83.0 Hz, *J* = 11.5 Hz, 2H), 4.37 (br s, 1H), 4.25–4.18 (m, 2H), 3.50 (dd, *J* = 14.9, 8.0 Hz, 1H), 3.42 (dd, *J* = 16.1, 8.1 Hz, 1H), 3.03 (d, *J* = 9.42 Hz, 1H), 2.00–1.93 (m, 1H), 1.92–1.84 (m, 1H), 1.78–1.70 (m, 1H), 1.24 (s, 9H), 0.89 (d, *J* = 6.8 Hz, 3H), 0.87 (d, *J* = 6.9 Hz, 3H); ^13^C{^1^H} NMR (150 MHz, CDCl_3_) δ 178.2, 174.5, 159.2, 151.8, 137.6, 128.6, 128.2, 128.0, 116.8, 77.3, 72.4, 62.0, 61.3, 60.5, 47.9, 38.9, 35.9, 29.1, 27.3, 19.0, 16.0; HRMS (ESI) *m*/*z* [M + H]^+^ calcd for C_25_H_36_N_3_O_5_S 490.2376; found 490.2363.
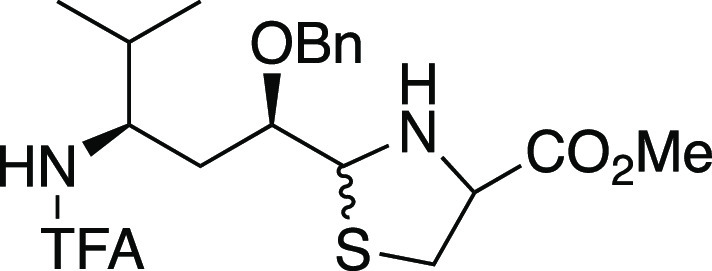


##### Methyl (2-((1′*R*,3′*R*)-3′-(Trifluoroacetamido)-1′-(benzyloxy)-4′-methylpentyl)thiazolidine-4-yl)carboxylate (**35**)

A solution of DMSO (0.02 mL, 0.27 mmol) in 0.128 mL CH_2_Cl_2_ was cooled to −78 °C followed by addition of oxalyl chloride (0.013 mL, 0.15 mmol). The mixture was stirred at −78 °C for 20 min followed by addition of alcohol **34**(48) (23 mg, 0.06 mmol) as a solution in 0.057 mL CH_2_Cl_2_. After 2 h at −78 °C, triethylamine (0.086 mL, 0.63 mmol) was added, and the temperature was allowed to rise to 0 °C over a period of 2 h. The reaction mixture at 0 °C was then diluted using 0.3 mL anhydrous ethyl alcohol followed by addition of cysteine methyl ester hydrochloride (18 mg, 0.1 mmol). After 12 h, concentration and flash chromatography (1:1 petroleum ether/ethyl acetate) afforded **35** (24 mg, 77% yield, mixture of diastereomers) as pale yellow oil: [α]_D_^22^ −32.5 (*c* 0.25, CHCl_3_); IR (film) 3583, 3314, 2960, 2925, 1742, 1721, 1710, 1203, 1181, 1160; ^1^H NMR (CDCl_3_, 400 MHz) δ 7.41–7.27 (m, 10H), 6.74 (d, *J* = 9.0 Hz, 1H), 6.63 (d, *J* = 9.2 Hz, 1H), 5.02 (d, *J* = 10.6 Hz, 1H), 4.81–4.72 (m, 2H), 4.67–4.53 (m, 3H), 4.10–3.91 (m, 3H), 3.87–3.81 (m, 1H), 3.80 (s, 3H), 3.78 (s, 3H), 3.43–3.34 (m, 1H), 3.30–3.23 (m, 2H), 2.93 (dd, *J* = 10.6, 7.6 Hz, 1H), 2.83 (dd, *J* = 10.1, 9.4 Hz, 2H), 2.02–1.95 (m, 2H), 1.89–1.77 (m, 4H), 1.75–1.69 (m, 2H), 0.92 (d, *J* = 6.8 Hz, 3H), 0.91–0.86 (m, 9H), some diastereomer peaks were not resolved; ^13^C{^1^H} NMR (100 MHz, CDCl_3_) 171.8, 171.3, 156.9 (q, *J* = 36 Hz), 137.70, 137.67, 128.5, 128.3, 128.2, 128.02, 127.99, 115.9 (q, *J* = 288 Hz), 111.6, 80.5, 76.7, 75.2, 73.9, 73.2, 71.7, 65.4, 64.3, 53.3, 52.9, 52.6, 52.5, 37.53, 37.45, 35.9, 34.3, 31.9, 31.8, 19.0, 18.5, 18.2, some diastereomer peaks were not resolved; MS (ESI) *m*/*z* (rel intensity) 471 ([M + Na]^+^, 12), 449 ([M + H]^+^, 100); HRMS (ESI) *m*/*z* [M + H]^+^ calcd for C_20_H_28_F_3_N_2_O_4_S 449.1722; found: 449.1714.
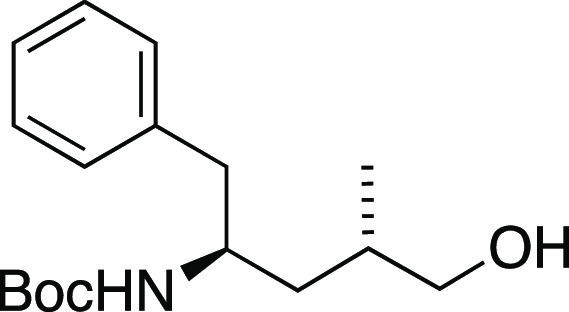


##### (2*S*,4*R*)-4-(*tert*-Butoxycarbonylamino)-2-methyl-5-phenyl-1-pentanol (**37**)

A solution of trifluoroacetyl amino alcohol **36**(48) (57 mg, 0.02 mmol) in a suspension of methanol–water (9.5 mL, 5:1) was cooled to 0 °C followed by addition of Ba(OH)_2_·8H_2_O (124 mg, 0.39 mmol). The reaction mixture was allowed to warm to room temperature over 3 h and then heated at 40 °C with an oil bath. After 12 h, the mixture was cooled to room temperature and concentrated. To a solution of the residual material in diethyl ether (2.7 mL) at 0 °C were added triethylamine (0.033 mL, 0.23 mmol) and Boc anhydride (17 mg, 0.078 mmol), and the reaction was allowed to reach ambient temperature. After 12 h, the reaction was quenched with saturated aqueous NaHCO_3_ and partitioned between water and CH_2_Cl_2_. The organic phase was washed with brine, dried over Na_2_SO_4_, and concentrated. Flash chromatography (petroleum ether to 1:1 petroleum ether/ethyl acetate) afforded the known alcohol **37**(28) (25 mg, 44%).
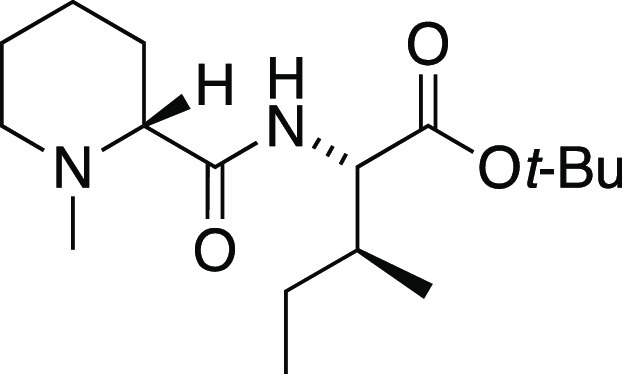


##### *N*-Methyl-d-pipecolyl-l-isoleucine *tert*-Butyl Ester (**S4**)

A solution of *N*-methyl-d-pipecolic acid^66^ (100 mg, 0.55 mmol) in DMF (9.1 mL) was cooled to 0 °C followed by addition of diisopropylethylamine (0.387 mL, 2.22 mmol). To this mixture was added l-isoleucine *tert*-butyl ester hydrochloride (56 mg, 0.35 mmol) followed by addition of diethyl cyanophosphate (DECP, 0.101 mL, 0.66 mmol). The reaction was allowed to reach ambient temperature. After 12 h, the reaction was quenched with water and partitioned between saturated aqueous NaHCO_3_ and EtOAc. The organic phase was washed with brine, dried over Na_2_SO_4_, and concentrated. Flash chromatography (1:1 petroleum ether/ethyl acetate to 1:4 petroleum ether/ethyl acetate) afforded **S4** (144 mg, 83% yield) as a pale yellow oil: [α]_D_^26^ +70.5 (c 0.96, CHCl_3_); IR (film) 3388, 2965, 2936, 2791, 1736, 1727, 1677, 1502, 1147, 847 cm^–1^; ^1^H NMR (CDCl_3_, 400 MHz) δ 6.96 (d, *J* = 9.5 Hz, 1H), 4.38 (dd, *J* = 9.2, 4.5 Hz, 1H), 2.83 (br d, *J* = 11.6 Hz, 1H), 2.41 (dd, *J* = 11.2, 3.4 Hz, 1H), 2.15 (s, 3H), 1.99 (ddd, *J* = 12.7, 11.6, 3.1 Hz, 1H), 1.88–1.80 (m, 2H), 1.71–1.62 (m, 1H), 1.60–1.46 (m, 2H), 1.46–1.36 (m, 2H), 1.38 (s, 9H), 1.28–1.03 (m, 2H), 0.92 (t, *J* = 6.7 Hz, 3H), 0.91 (d, *J* = 6.8 Hz, 3H); ^13^C{^1^H} NMR (100 MHz, CDCl_3_) 174.0, 170.7, 81.4, 69.6, 56.1, 55.3, 44.7, 37.7, 30.6, 27.9, 25.2, 25.1, 23.2, 15.6, 11.6; MS (ESI) *m*/*z* (rel intensity) 335 ([M + Na]^+^, 4), 313 ([M + H]^+^, 100); HRMS (ESI) *m*/*z* [M + H]^+^ calcd for C_17_H_33_N_2_O_3_ 313.2491; found 313.2498.
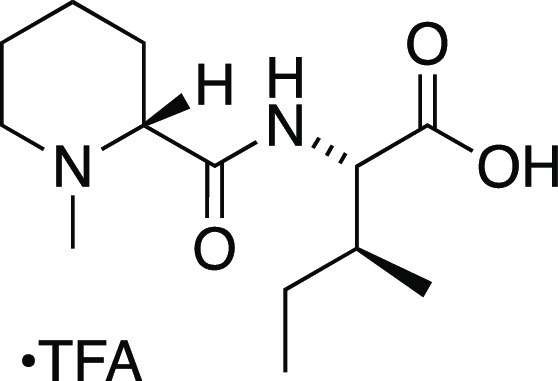


##### *N*-Methyl-d-pipecolyl-l-isoleucine Trifluoroacetate Salt (**42**)

A solution of *tert*-butyl ester **S4** (50 mg, 0.16 mmol) in dichloromethane (1.6 mL) was treated with trifluoroacetic acid (0.204 mL, 2.66 mmol) followed by reflux for 4 h with heat applied by oil bath. The mixture was then cooled to room temperature and concentrated to afford the carboxylic acid **42**(62) as the TFA salt, which was used without further purification. MS (ESI) *m*/*z* (rel intensity) 257 ([M + H]^+^, 100).
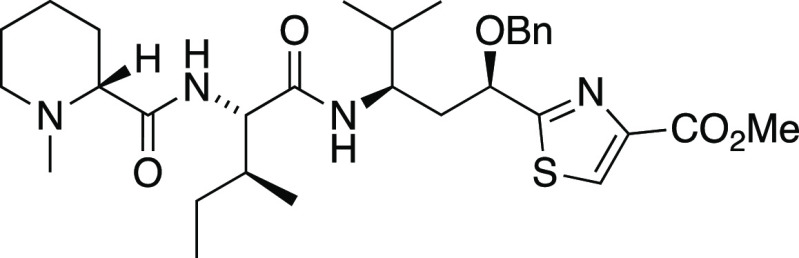


##### *N*-Methyl-d-pipecolyl-l-isoleucinyl-(*O*-benzyl)tubuvaline Methyl Ester (**43**)

A solution of trifluoroacetyl amino ester **10** (12 mg, 0.02 mmol) in methanol–water (1 mL, 5:1) was cooled to 0 °C followed by addition of Ba(OH)_2_·8H_2_O (42 mg, 0.16 mmol). The reaction mixture was allowed to warm ambient temperature over 3 h and then maintained at 40 °C overnight with heat applied by oil bath. The mixture was then cooled to room temperature and concentrated to afford the crude amino acid: MS (ESI) *m*/*z* (rel intensity) 357 ([M + Na]^+^, 5), 335 ([M + H]^+^, 100). The crude amino acid was treated with methanolic HCl (2 mL of a stock solution prepared from 5 mL methanol and 0.71 mL acetyl chloride) at 0 °C for 10 min and then heated at reflux for 2.5 h. The reaction mixture was cooled to 0 °C and concentrated. The residue was taken up in toluene and concentrated to ensure azeotropic removal of traces of acetic acid. Flash chromatography (2:1 petroleum ether/ethyl acetate to 9:1 dichloromethane/methanol) afforded the Tuv methyl ester **41** (9 mg, 96% yield) as a pale yellow oil; MS (ESI) *m*/*z* (rel intensity) 371 ([M + Na]^+^, 3), 349 ([M + H]^+^, 100). This material was used immediately in the coupling reaction.

To a solution of the Mep-Ile dipeptide **42** (12 mg, 0.03 mmol) in anhydrous ethyl acetate (0.212 mL) was added *N*-methyl morpholine (0.004 mL, 0.03 mmol). The mixture was cooled to −10 °C and isobutyl chloroformate (0.005 mL, 0.034 mmol) was added via syringe, followed by a solution of Tuv amino ester **41** (6 mg, 0.01 mmol) in anhydrous ethyl acetate (0.085 mL) via cannula. The reaction mixture was allowed to warm slowly to ambient temperature. After 12 h, the reaction mixture was partitioned between water and EtOAc. The organic phase was washed with brine, dried over MgSO_4_, and concentrated. Flash chromatography (petroleum ether to dichloromethane to 9:1 dichloromethane/methanol) afforded the tripeptide **43** (10 mg, > 99%) as a pale yellow oil; [α]_D_^25^ −2.7 (*c* 0.07, CDCl_3_); IR (film) 3583, 2959, 2925, 1737, 1726, 1691, 1678; ^1^H NMR (CDCl_3_, 400 MHz) δ 8.17 (s, 1H), 7.55–7.28 (m, 5H), 7.00 (d, *J* = 8.5 Hz, 1H), 6.18 (d, *J* = 9.9 Hz, 1H), 4.85 (dd, *J* = 9.2, 4.2 Hz, 1H), 4.56 (ABq, *J* = 10.3 Hz, *Δν* = 38 Hz, 2H), 4.33–4.23 (m, 1H), 4.09 (dd, *J* = 8.6, 8.4 Hz, 1H), 3.96 (s, 3H), 2.91 (br d, *J* = 11.7 Hz, 1H), 2.50 (dd, *J* = 11.2, 3.4 Hz, 1H), 2.22 (s, 3H), 2.09–1.86 (m, 6H), 1.81–1.69 (m, 2H), 1.57–1.47 (m, 2H), 1.44–1.33 (m, 1H), 1.24–1.13 (m, 2H), 0.97 (d, *J* = 6.7 Hz, 3H), 0.96–0.88 (m, 3H), 0.87 (d, *J* = 2.4 Hz, 3H), 0.86 (d, *J* = 2.7 Hz, 3H); ^13^C{^1^H} NMR (100 MHz, CDCl_3_) 175.9, 174.9, 171.0, 161.8, 146.9, 137.1, 128.6, 128.5, 128.2, 128.0, 77.2, 73.5, 69.7, 57.8, 55.4, 52.5, 50.5, 45.1, 40.2, 35.3, 32.2, 30.9, 25.2, 24.9, 23.3, 19.0, 17.8, 16.2, 10.7; MS (ESI) *m*/*z* (rel intensity) 609 ([M + Na]^+^, 100), 587 ([M + H]^+^, 15); HRMS (ESI) *m*/*z* [M + H]^+^ calcd for C_31_H_47_N_4_O_5_S 587.3267; Found: 587.3255.
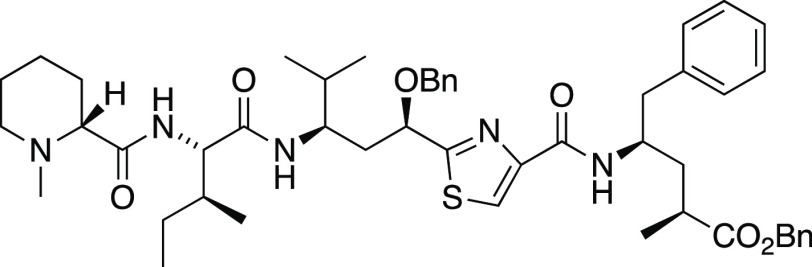


##### 11-*O*-Benzyltubulysin V Benzyl Ester (**12**)

To a solution of tripeptide **43** (5 mg, 8 μmol) in THF (0.22 mL) was added an aqueous solution of LiOH·H_2_O (1 N, 0.021 mL). The reaction mixture was stirred for 72 h and then acidified using TFA to pH 1–2. The mixture was partitioned between water and ethyl acetate, and the organic phase was dried over Na_2_SO_4_. Concentration afforded carboxylic acid **44** that was used without further purification; MS (ESI) *m*/*z* (rel intensity) 595 ([M + Na]^+^, 20), 573 ([M + H]^+^, 100).

To a solution of *N*-Boc amine **39**(34) (6 mg, 0.015 mmol) in CH_2_Cl_2_ (0.02 mL) was added TFA (0.02 mL) at ambient temperature. After 2 h the mixture was concentrated to furnish the trifluoroacetate salt of Tup benzyl ester (**40**) that was used without further purification.

To a solution of carboxylic acid **44** (5 mg, 7 μmol) and *i*-Pr_2_NEt (5.2 μL, 0.029 mmol) in DMF (0.01 mL) at 0 °C was added a solution of **40** (4.5 mg, 0.015 mmol) in DMF (0.110 mL) followed by diethyl cyanophosphonate (DECP, 1.2 μL, 8 μmol). The mixture was allowed to warm to ambient temperature. After 12 h, the reaction was quenched with water and partitioned between saturated aqueous NaHCO_3_ and EtOAc. The organic phase was washed with brine and dried over Na_2_SO_4_. Flash chromatography (CH_2_Cl_2_ to 24:1 CH_2_Cl_2_/MeOH) afforded tetrapeptide **12** (7 mg, > 99%) as a pale yellow oil: ^1^H NMR (CDCl_3_, 400 MHz) δ 8.02 (s, 1H), 7.66–7.28 (m, 15H), 7.03 (d, *J* = 8.8 Hz, 1H), 6.27–6.08 (d, *J* = 9.8 Hz, 1H), 5.09 (q, *J* = 12.3 Hz, 2H), 4.68 (dd, *J* = 10.6, 2.6 Hz, 1H), 4.61–4.42 (m, 3H), 4.33–4.21 (m, 1H), 4.20–4.07 (m, 2H), 3.02–2.83 (m, 3H), 2.76–2.63 (m, 1H), 2.52 (dd, *J* = 11.6, 3.5 Hz, 1H), 2.23 (s, 3H), 2.11–1.98 (m, 11H), 1.97–1.70 (m, 4H), 1.20 (d, *J* = 7.1 Hz, 3H), 0.96 (d, *J* = 6.7 Hz, 3H), 0.93–0.87 (m, apparent overlap of doublets, 9H); MS (ESI) *m*/*z* (rel intensity) 874 ([M + Na]^+^, 100), 852 ([M + H]^+^, 75); HRMS (ESI) *m*/*z* [M + H]^+^ calcd for C_49_H_65_N_5_O_6_S 852.4734; found 852.4723. Due to material loss, ^13^C NMR data were unavailable for this compound. Treatment of **12** with SnCl_4_, using the procedure given for **45**, furnished a yellow oil with mass spectral data consistent with 2-fold debenzylation: HRMS (ESI) *m*/*z* [M + H]^+^ calcd for C_35_H_53_N_5_O_6_S 672.3795; found 672.3801.
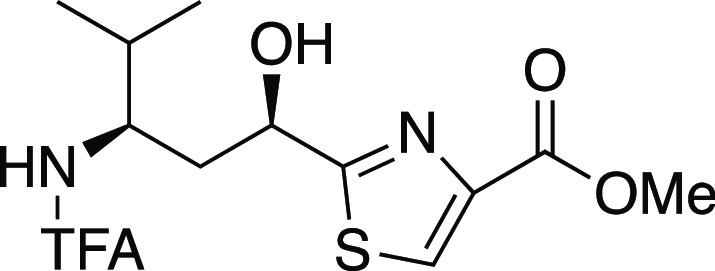


##### Methyl (2-((1′*R*,3′*R*)-1′-Hydroxy-4′-methyl-3′-(trifluoroacetamido)pent-1′-yl)thiazol-4-yl)carboxylate (**45**)

To a solution of *N*-TFA amino ester **10** (23 mg, 0.05 mmol) in CH_2_Cl_2_ (0.25 mL) was added tin(IV) chloride (0.03 mL, 0.25 mmol) at ambient temperature. The reaction mixture was then heated at reflux for 25 h. After cooling to 0 °C the reaction was quenched by dropwise addition of satd NaHCO_3_ and extracted with CH_2_Cl_2_. The organic phase was washed with brine and dried over Na_2_SO_4_. Concentration and flash chromatography afforded alcohol **45**(67) (15 mg, 83% yield) as a pale yellow oil: ^1^H NMR (CDCl_3_, 400 MHz) δ 8.15 (s, 1H), 6.46 (d, *J* = 9.5 Hz, 1H), 4.96 (ddd, *J* = 11.1, 4.8, 2.4 Hz, 1H), 4.24 (d, *J* = 5.0 Hz, 1H), 4.08–4.16 (m, 1H), 3.93 (s, 3H), 2.26 (ddd, *J* = 14.1, 11.6, 2.4 Hz, 1H), 2.02–1.83 (m, 2H), 1.00 (d, *J* = 7 Hz, 3H), 0.99 (d, *J* = 7 Hz, 3H); ^13^C{^1^H} NMR (100 MHz, CDCl_3_) 175.8, 161.8, 158.6 (q, *J* = 37 Hz), 146.6, 127.8, 115.8 (q, *J* = 287 Hz), 68.7, 52.6, 52.4, 40.2, 31.8, 19.2, 18.2; MS (ESI) *m*/*z* (rel intensity) 377 ([M + Na]^+^, 100), 355 ([M + H]^+^, 8).

## Supporting Information Available

Comparisons of tubulysin syntheses, ^1^H and ^13^C NMR spectra for new compounds, enantiomer ratio and configuration assignment of (*R*)-**19** (PDF)
