# Supplementary material for: Diastereocontrol in Radical Addition to β-Benzyloxy Hydrazones: Revised Approach to Tubuvaline and Synthesis of O-Benzyltubulysin V Benzyl Ester
Source: J Org Chem. 2021 Oct 12;86(21):15139–52. doi: 10.1021/acs.joc.1c01798 (PMC8576829; doi:10.1021/acs.joc.1c01798)
Supplement: Supplementary file 1 — jo1c01798_si_001.pdf [file jo1c01798_si_001.pdf]

# Diastereocontrol in Radical Addition to β-Benzyloxy Hydrazones: Revised Approach to Tubuvaline and Synthesis of *O*-Benzyltubulysin V Benzyl Ester

Manshu Li, Koushik Banerjee, and Gregory K. Friestad*

*Department of Chemistry, University of Iowa, Iowa City, Iowa 52242 USA*

email: gregory-friestad@uiowa.edu

## Supporting Information

**Contents**

Brief Review of Synthetic Approaches to Tubuvaline and Tubulysins S2

$^{1}$H NMR and $^{13}$C NMR Spectra for New Compounds S8

Enantiomer Ratio and Configuration Assignment of (*R*)-**19** S49

## Brief Review of Synthetic Approaches to Tubuvaline and Tubulysins

To put the present tubulysin study into a larger context, we briefly review the diverse and creative strategies that complement our work. The primary drivers of innovation tend to focus on stereocontrol at the alcohol- or ester-bearing center at C11, stereocontrol at the C13 chiral amine, and introduction of the thiazole.

In Wipf's route to a Tuv-Tup dipeptide,[1] the Tuv C13 stereocenter was obtained from valine, while the C11 OH was installed by substrate-controlled enolate oxidation; a multistep dipeptide cyclodehydration sequence was applied to construct the thiazole. A revised approach, en route to $N_{14}$-desacetoxytubulysin H,[2] employed thiazole anion addition to a homovalinal derivative, generating alcohol **A** (dr 2:1). An oxidation and reduction sequence corrected the configuration of the undesired diastereomer.[3]

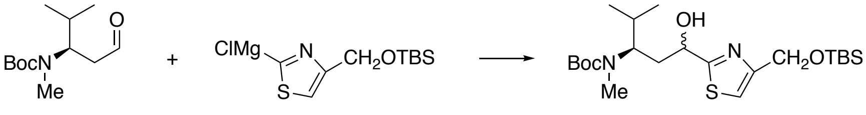

**A** (dr 2:1)

Dömling and Wessjohann reported total synthesis of C2-epi-tubulysins U and V,[4] with the Tuv C13 configuration originating in a valine derivative. A multicomponent reaction (MCR) constructed the thiazole **B** with modest stereocontrol at C11 (dr 75:25). A similar MCR approach to Tuv was used in the 2009 synthesis of tubulysin B by Wessjohann et al.[5] More recently, a catalytic asymmetric Passerini reaction MCR was achieved by Dömling et al. with good stereoselectivity (dr 92:8), and dehydrative cyclization to the thiazole and acyl transfer gave the Mep-Ile-Tuv tripeptide of tubulysins.[6]

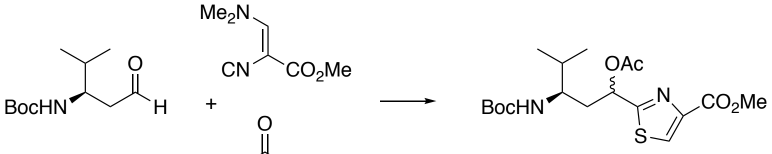

**B** (dr 75:25)

Ellman reported the first total synthesis of tubulysin D in 2006 using a strategy that constructed the C11 and C13 stereocenters of Tuv by sequential metalloenamine aldol addition and hydride reduction.[7] Both reactions employed the *N*-sulfinyl group as a chiral auxiliary, and the sequence

afforded tubuvaline precursor **C** with 92:8 and 91:9 diastereomer ratios at C11 and C13, respectively.

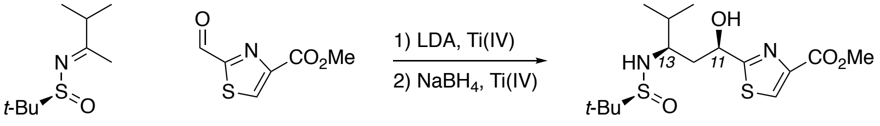


**C** (dr 92:8 at C11, 91:9 at C13)

In 2007, Zanda reported a total synthesis of tubulysin U and V.[8] The Tuv C13 stereocenter was constructed via racemic aza-Michael addition, with C11 controlled by oxazaborolidine-catalyzed ("CBS") borane reduction of a β-aminoketone. This led to a separable mixture of **XX** and 13-epi-**XX** in 67% yield. Zanda also reported a Mannich addition to generate the racemic β-aminoketone that was then reduced as before.[9]

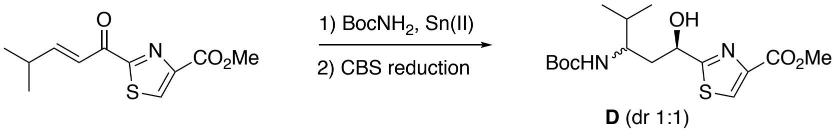


**D** (dr 1:1)

A gram-scale synthesis of Tuv-Tup dipeptide fragment was reported by Chandrasekhar in 2009.[10] The C13 stereocenter (Tuv numbering) originated in *L*-valine, while the C11 alcohol was constructed by organocatalytic α-hydroxylation to afford **E** with excellent diastereoselectivity. After functional group manipulations, condensation with a cysteine derivative and oxidation afforded the thiazole.

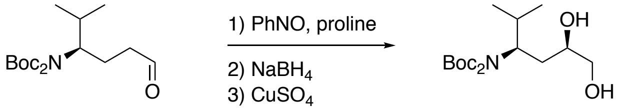


**E** (dr 96:4)

Fecik reported syntheses of several simplified tubulysin analogs in 2008, revealing the importance of the C11 stereocenter to cytotoxic potency.[11] The C13 stereocenter in the Tuv fragment was obtained from a natural source, while C11 was generated in the ketone oxidation state via addition to a Weinreb amide to furnish **F**, a thiazolyl ketone analog of Tuv. CBS reduction was then employed to control the C11 configuration. The 2009 synthesis by Kazmaier and Müller of a C11 des-acetoxy tubulysin analog, termed pretubulysin for its proposed biosynthetic role, confirmed the importance of the Tuv C11 functionality; its absence diminished the potency ten-

fold.[12] The Weinreb amide approach of Fecik was also used by Parker et al. en route to a tubulysin analog as payload for antibody-drug conjugates.[13]

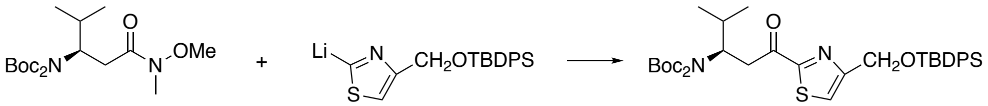


**F**

Tamura et al. exploited a diastereoselective 1,3-dipolar nitrone cycloaddition to introduce the C11 and C13 stereocenters using both *D*-gulosyl and camphorsultam chiral auxiliaries, setting both of the configurations in **G**.[14] The thiazole moiety was established in a multistep sequence, and N–O bond cleavage of the isoxazolidine ring then afforded Tuv methyl ester.

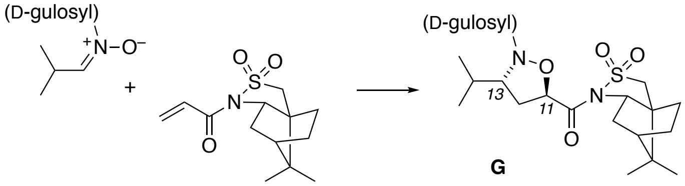


**G**

Chen targeted tubulysin U and its C4 epimer, employing a Tuv synthesis via thiazolyl ketone enolate addition to a chiral sulfinimine to generate the C13 configuration (dr 103:1).[15] Diastereoselective reduction of the ketone led to Tuv fragment **H** (dr 10:1).

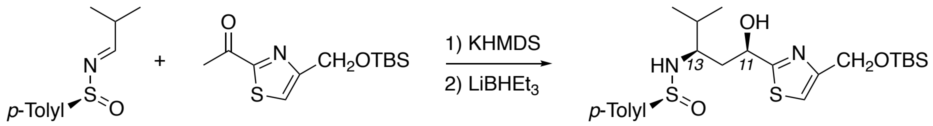


**H** (dr 10:1 at C11, 103:1 at C13)

The synthesis of tubulysin V reported in 2013 by Lin et al. exploited allyl addition to chiral sulfinimines to generate homovalinal, setting the C13 stereocenter (dr 96:4), and then adopted the previously described Wessjohann multicomponent coupling to prepare the thiazole and install the C11 configuration (dr 1:2.4).[16]

In 2016, Nicolaou reported syntheses of numerous tubulysin analogues, with the Tuv C13 stereocenter sourced from valine.[17] Reminiscent of the Wipf approach, a coupling of a homovalinal derivative with a thiazole nucleophile was enabled in this case by C-H activation. The C11 stereocenter was constructed by CBS reduction.

Other approaches to tubuvaline and analogs include a proline-catalyzed aldol reaction followed by substrate-controlled reductive amination,[18] asymmetric hetero-Diels–Alder reaction and reductive amination to generate a constrained tubuvaline analog,[19] and addition of isopropyl Grignard to a β-alkoxysulfinimine.[20]

**Table S1. Comparison of C11 and C13 stereocontrol in Tuv syntheses.**

| Contributor, Year | C11 Stereocontrol | C13 Stereocontrol |
|---|---|---|
| **Friestad, 2004** | **Enolate allylation, >98:2** | **Radical addition to C=N, >98:2** |
| Wipf 2004 | Enolate oxidation, >98:2 | *(natural source)* |
| Dömling, 2006 | MCR, 1:2.4 | *(natural source)* |
| Ellman, 2006 | Metalloenamine aldol, 92:8 | Sulfinimine reduction, 91:9 |
| Zanda, 2007 | CBS reduction, 95:5 | aza-Michael, 1:1 |
| Wipf, 2007 | 1,3-Diastereocontrol, 2:1 | *(natural source)* |
| Fecik, 2009 | CBS reduction, >98:2[a] | *(natural source)* |
| Chandrasekhar, 2009 | Enamine oxidation, 96:4 | *(natural source)* |
| Tamura, 2009 | Both C11 and C13 by nitrone cycloaddition, 85:15 | |
| Chen, 2013 | Borohydride reduction, 10:1 | Mannich, 103:1 |
| Lin, 2013 | *(natural source)* | Allylindium addition to C=N, 96:4 |
| Wei, 2016 | MCR, 1:2.4 | i-PrMgBr addition to C=N, 98:2 |
| Nicolau, 2016 | CBS reduction, >98:2[b] | *(natural source)* |
| **Friestad, this work** | **Keck allylation, 10:1** | **Radical addition to C=N, >98:2** |

[a] Almost exclusively one configuration, ratio not reported

[b] Single diastereomer after purification, ratio not reported

**Table S2. Comparison of C2 and C4 stereocontrol in Tup syntheses.**

| Contributor, Year | C2 Stereocontrol | C4 Stereocontrol |
|---|---|---|
| **Friestad, 2004** | ***(natural source)*** | **Radical addition to C=N, >98:2** |
| Wipf 2004 | Hydrogenation, 3:1 | *(natural source)* |
| Dömling, 2006 | Enolate alkylation, 19:81 | *(commercial)* |
| Ellman, 2006 | Both C2 and C4 by reductive addition to acrylate, 80:15:3:2 | |
| Zanda, 2007 | Hydrogenation, 1:1 | *(natural source)* |
| Fecik, 2009 | Enolate alkylation, 10:1 | *(natural source)* |
| Chandrasekhar, 2009 | *(natural source)* | Enamine oxidation and $S_N2$, 99:1 |
| Tamura, 2009 | Evans aldol, >98:2 | *(natural source)* |
| Chen, 2013 | Enolate allylation, 97:3[a] | BnMgBr addition to C=N, 6.6:1 |
| Lin, 2013 | Inversion via enolate, 6:1 | Allylzinc addition to C=N, 95:5 |
| Zanda, 2013 | Hydrogenation, 2:1 | *(natural source)* |
| Wei, 2016 | Enolate alkylation, >98:2 | Sulfinimine reduction, >9:1[b] |

[a] Ratio from literature cited by authors

[b] Minor isomer separated, ratio not reported

[1] Wipf, P.; Takada, T.; Rishel, M. J. Synthesis of the Tubuvaline-Tubuphenylalanine (Tuv-Tup) Fragment of Tubulysin. *Org. Lett.* **2004**, *6*, 4057-4060.

[2] Wipf, P.; Wang, Z. Total Synthesis of N14-Desacetoxytubulysin H. *Org. Lett.* **2007**, *9*, 1605-1607.

[3] Colombo, R.; Wang, Z.; Han, J.; Balachandran, R.; Daghestani, H. N.; Camarco, D. P.; Vogt, A.; Day, B. W.; Mendel, D.; Wipf, P. Total Synthesis and Biological Evaluation of Tubulysin Analogues. *J. Org. Chem.* **2016**, *81*, 10302-10320.

[4] Dömling, A.; Beck, B.; Eichelberger, U.; Sakamuri, S.; Menon, S.; Chen, Q.-Z.; Lu, Y.; Wessjohann, L. A. Total Synthesis of Tubulysin U and V. *Angew. Chem. Int. Ed.* **2006**, *45*, 7235-7239.

[5] Pando, O.; Dörner, S.; Preusentanz, R.; Denkert, A.; Porzel, A.; Richter, W.; Wessjohann, L. First Total Synthesis of Tubulysin B. *Org. Lett.* **2009**, *11*, 5567-5569.

[6] Vishwanatha, T. M.; Giepmans, B.; Goda, S. K.; Dömling, A. Tubulysin Synthesis Featuring Stereoselective Catalysis and Highly Convergent Multicomponent Assembly. *Org. Lett.* **2020**, *22*, 5396-5400.

[7] (a) Peltier, H. M.; McMahon, J. P.; Patterson, A. W.; Ellman, J. A. The Total Synthesis of Tubulysin D. *J. Am. Chem. Soc.* **2006**, *128*, 16018-16019. (b) Patterson, A. W.; Peltier, H. M.; Sasse, F.; Ellman, J. A., Design, Synthesis, and Biological Properties of Highly Potent Tubulysin D Analogues. *Chem. Eur. J.* **2007**, *13* (34), 9534-9541. (c) Patterson, A. W.; Peltier, H. M.; Ellman, J. A. Expedient Synthesis of N-Methyl Tubulysin Analogues with High Cytotoxicity. *J. Org. Chem.* **2008**, *73*, 4362-4369.

[8] Sani, M.; Fossati, G.; Huguenot, F.; Zanda, M. Total Synthesis of Tubulysins U and V. *Angew. Chem. Int. Ed.* **2007**, *46*, 3526-3529.

[9] Shankar, S. P.; Jagodzinska, M.; Malpezzi, L.; Lazzari, P.; Manca, I.; Greig, I. R.; Sani, M.; Zanda, M. Synthesis and structure–activity relationship studies of novel tubulysin U analogues – effect on cytotoxicity of structural variations in the tubuvaline fragment. *Org. Biomol. Chem.* **2013**, *11*, 2273-2287.

[10] Chandrasekhar, S.; Mahipal, B.; Kavitha, M. Toward Tubulysin: Gram-Scale Synthesis of Tubuvaline-Tubuphenylalanine Fragment. *J. Org. Chem.* **2009**, *74*, 9531-9534.

[11] Raghavan, B.; Balasubramanian, R.; Steele, J. C.; Sackett, D. L.; Fecik, R. A. Cytotoxic Simplified Tubulysin Analogues. *J. Med. Chem.* **2008**, *51*, 1530-1533.

[12] Ullrich, A.; Chai, Y.; Pistorius, D.; Elnakady, Y. A.; Herrmann, J. E.; Weissman, K. J.; Kazmaier, U.; Müller, R. Pretubulysin, a Potent and Chemically Accessible Tubulysin Precursor from Angiococcus disciformis. *Angew. Chem. Int. Ed.* **2009**, *48*, 4422-4425.

[13] (a) Parker, J. S.; McCormick, M.; Anderson, D. W.; Maltman, B. A.; Gingipalli, L.; Toader, D. The Development and Scale-Up of an Antibody Drug Conjugate Tubulysin Payload. *Org. Process Res. Dev.* **2017**, *21*, 1602-1609. (b) Toader, D.; Wang, F.; Gingipalli, L.; Vasbinder, M.; Roth, M.; Mao, S.; Block, M.; Harper, J.; Thota, S.; Su, M.; Ma, J.; Bedian, V.; Kamal, A. Structure–Cytotoxicity Relationships of Analogues of N14-Desacetoxytubulysin H. *J. Med. Chem.* **2016**, *59*, 10781-10787.

[14] (a) Shibue, T.; Hirai, T.; Okamoto, I.; Morita, N.; Masu, H.; Azumaya, I.; Tamura, O. Stereoselective synthesis of tubuvaline methyl ester and tubuphenylalanine, components of tubulysins, tubulin polymerization inhibitors. *Tetrahedron Lett.* **2009**, *50*, 3845-3848. Shibue, T.; Hirai, T.; Okamoto, I.; Morita, N.; Masu, H.; Azumaya, I.; Tamura, O. Total Syntheses of Tubulysins. *Chem. Eur. J.* **2010**, *16*, 11678-11688.

[15] Yang, X.-D.; Dong, C.-M.; Chen, J.; Ding, Y.-H.; Liu, Q.; Ma, X.-Y.; Zhang, Q.; Chen, Y. Total Synthesis of Tubulysin U and Its C-4 Epimer. *Chem. Asian J.* **2013**, *8*, 1213-1222.

[16] Wang, R.; Tian, P.; Lin, G. Stereoselective Total Synthesis of Tubulysin V. *Chin. J. Chem.* **2013**, *31*, 40-48.

[17] (a) Nicolaou, K. C.; Yin, J.; Mandal, D.; Erande, R. D.; Klahn, P.; Jin, M.; Aujay, M.; Sandoval, J.; Gavrilyuk, J.; Vourloumis, D. Total Synthesis and Biological Evaluation of Natural and Designed Tubulysins. *Journal of the American Chemical Society* **2016,** *138* (5), 1698-1708. (b) Nicolaou, K. C.; Erande, R. D.; Yin, J.; Vourloumis, D.; Aujay, M.; Sandoval, J.; Munneke, S.; Gavrilyuk, J. Improved Total Synthesis of Tubulysins and Design, Synthesis, and Biological Evaluation of New Tubulysins with Highly Potent Cytotoxicities against Cancer Cells as Potential Payloads for Antibody–Drug Conjugates. *J. Am. Chem. Soc.* **2018**, *140*, 3690-3711.

[18] Paladhi, S.; Das, J.; Samanta, M.; Dash, J. Asymmetric Aldol Reaction of Thiazole-Carbaldehydes: Regio- and Stereoselective Synthesis of Tubuvalin Analogues. *Adv. Synth. Catal.* **2014**, *356*, 3370-3376.

[19] Park, Y.; Lee, J. K.; Ryu, J.-S. Synthesis of a Cyclic Analogue of Tuv N-Methyl Tubulysin. *Synlett* **2015**, *26*, 1063-1068.
[20] Tao, W.; Zhou, W.; Zhou, Z.; Si, C.-M.; Sun, X. Wei, B.-G. An enantioselective total synthesis of tubulysin V. *Tetrahedron* **2016**, *72*, 5928-5933.

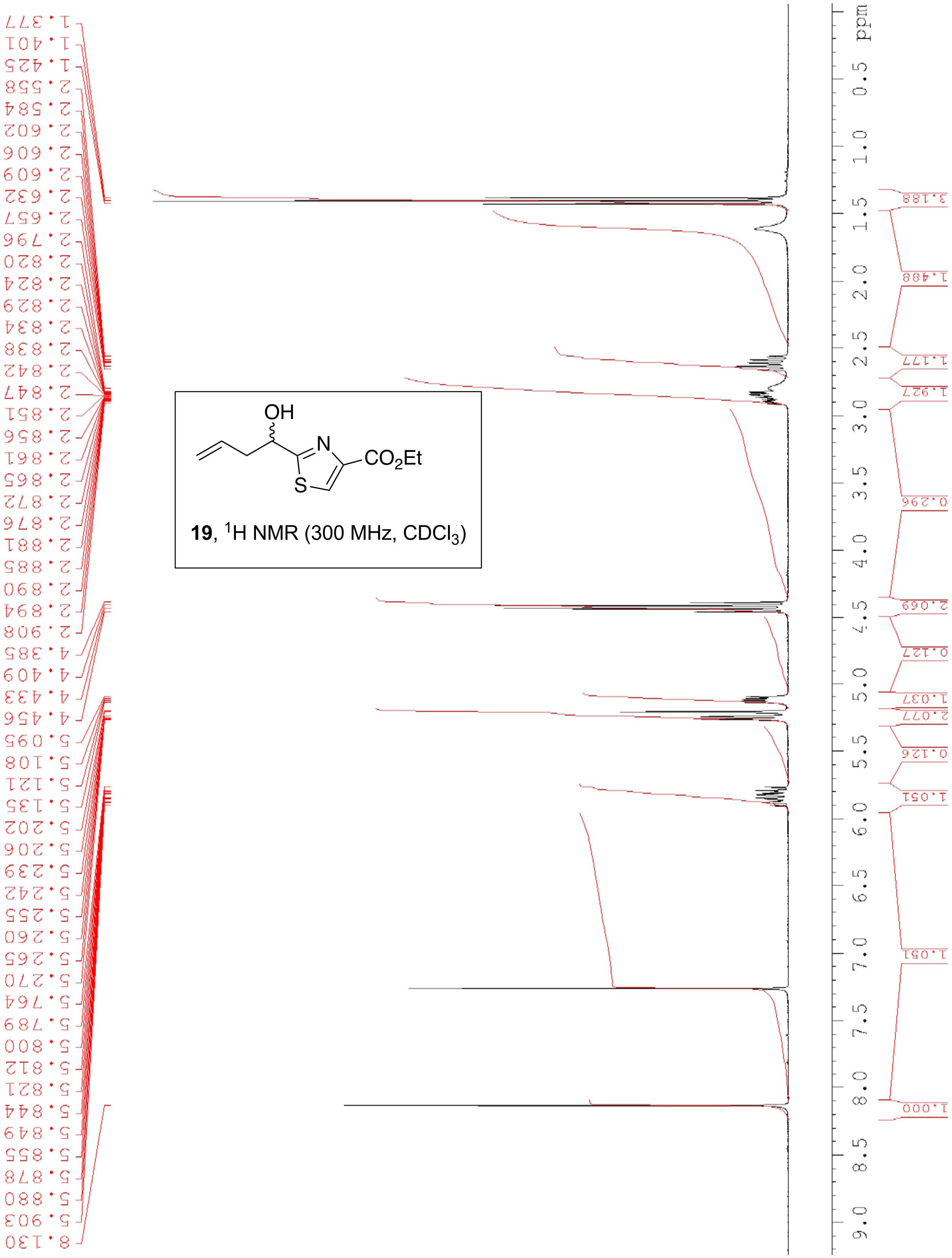


**19**, $^1H$ NMR (300 MHz, $CDCl_3$)

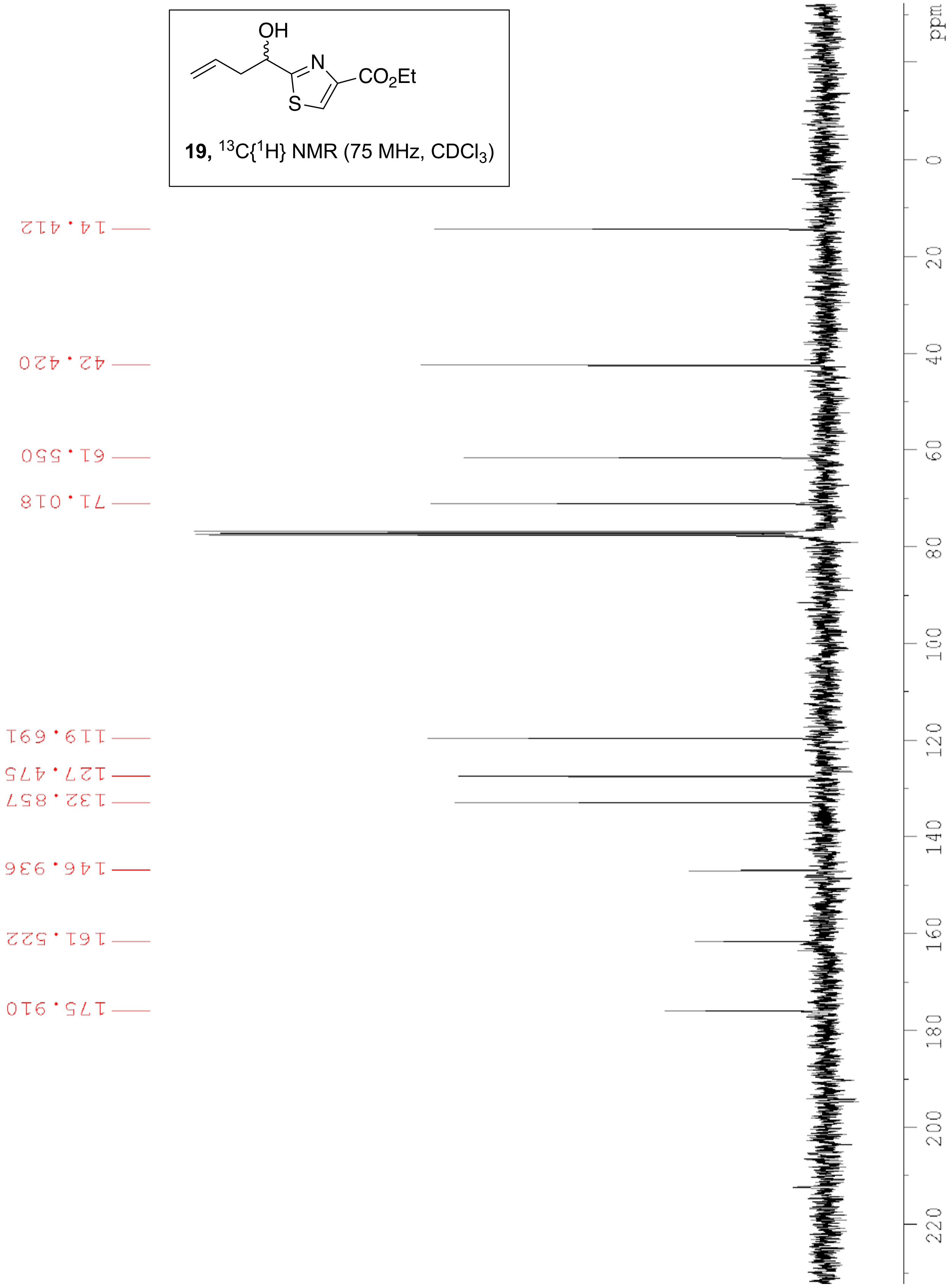


**19,** $^{13}C\{^1H\}$ NMR (75 MHz, $CDCl_3$)

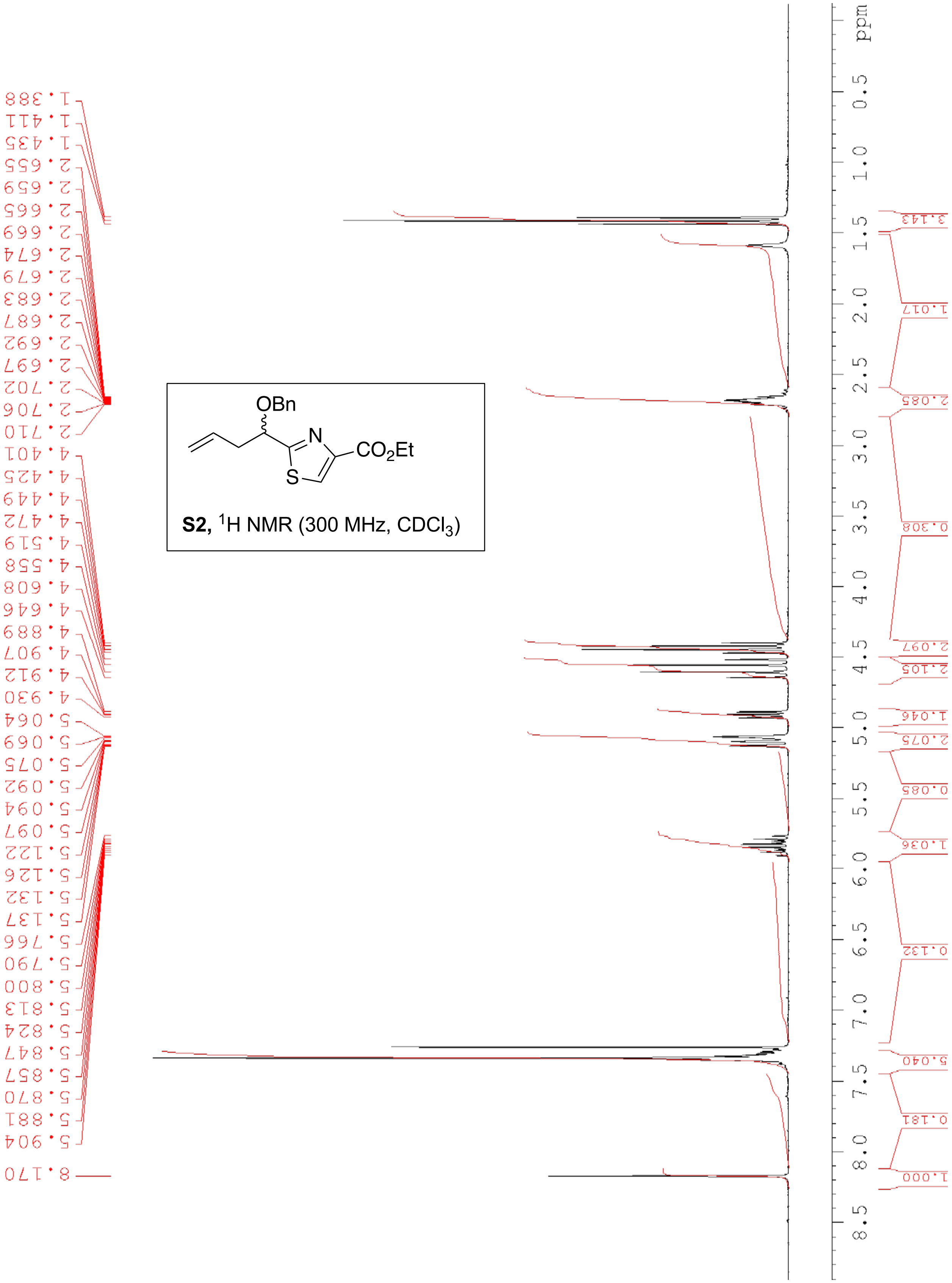

**S2,** $^{1}$H NMR (300 MHz, $CDCl_3$)

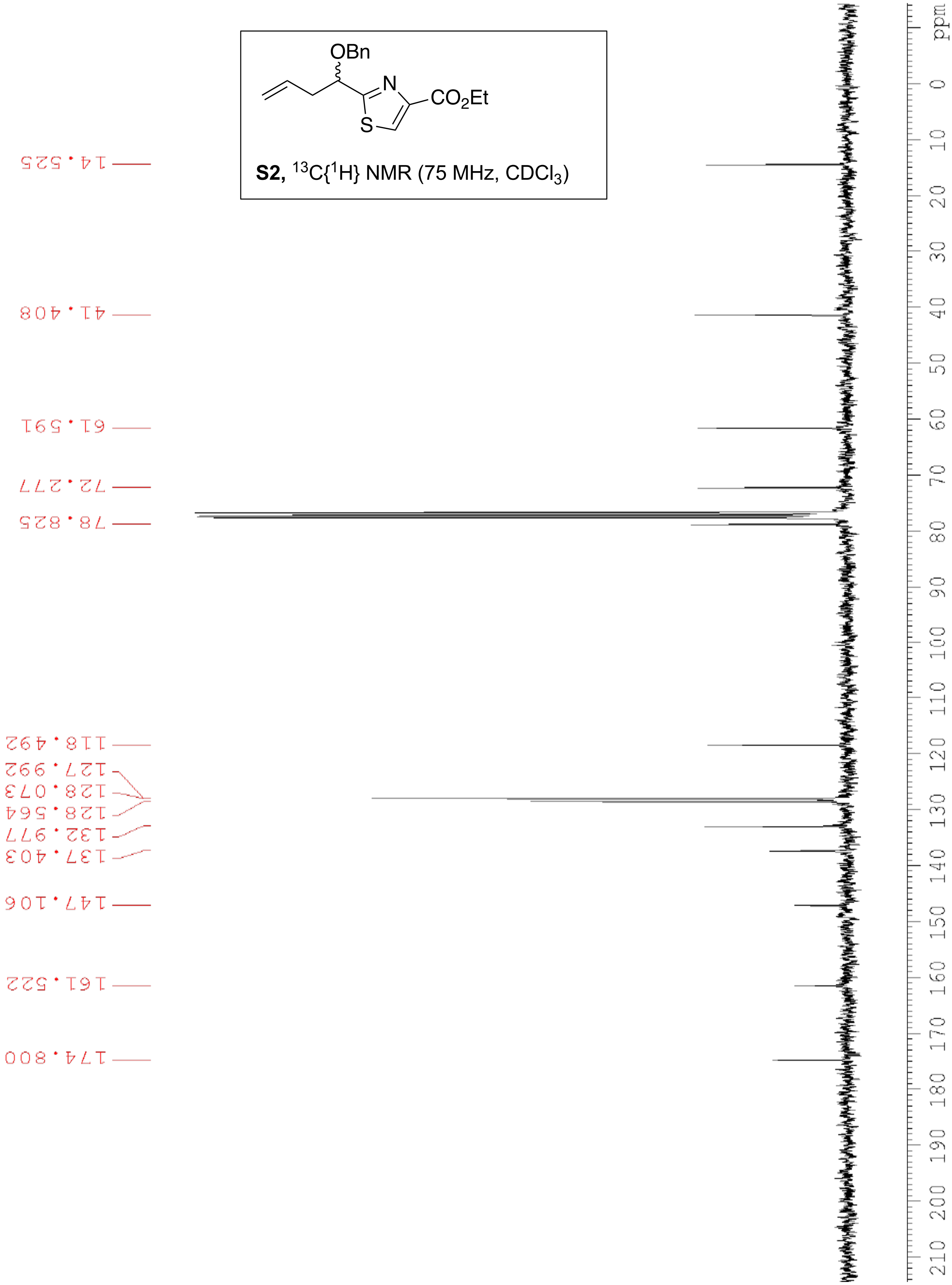
OBn
N
S
$CO_2Et$
**S2,** $^{13}C\{^1H\}$ NMR (75 MHz, $CDCl_3$)
14.525
41.408
61.591
72.277
78.825
118.492
127.992
128.073
128.564
132.977
137.403
147.106
161.522
174.800
ppm
0
10
20
30
40
50
60
70
80
90
100
110
120
130
140
150
160
170
180
190
200
210

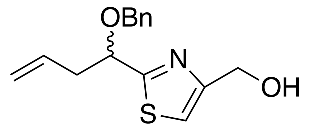


**20,** $^{1}$H NMR (300 MHz, $CDCl_3$)

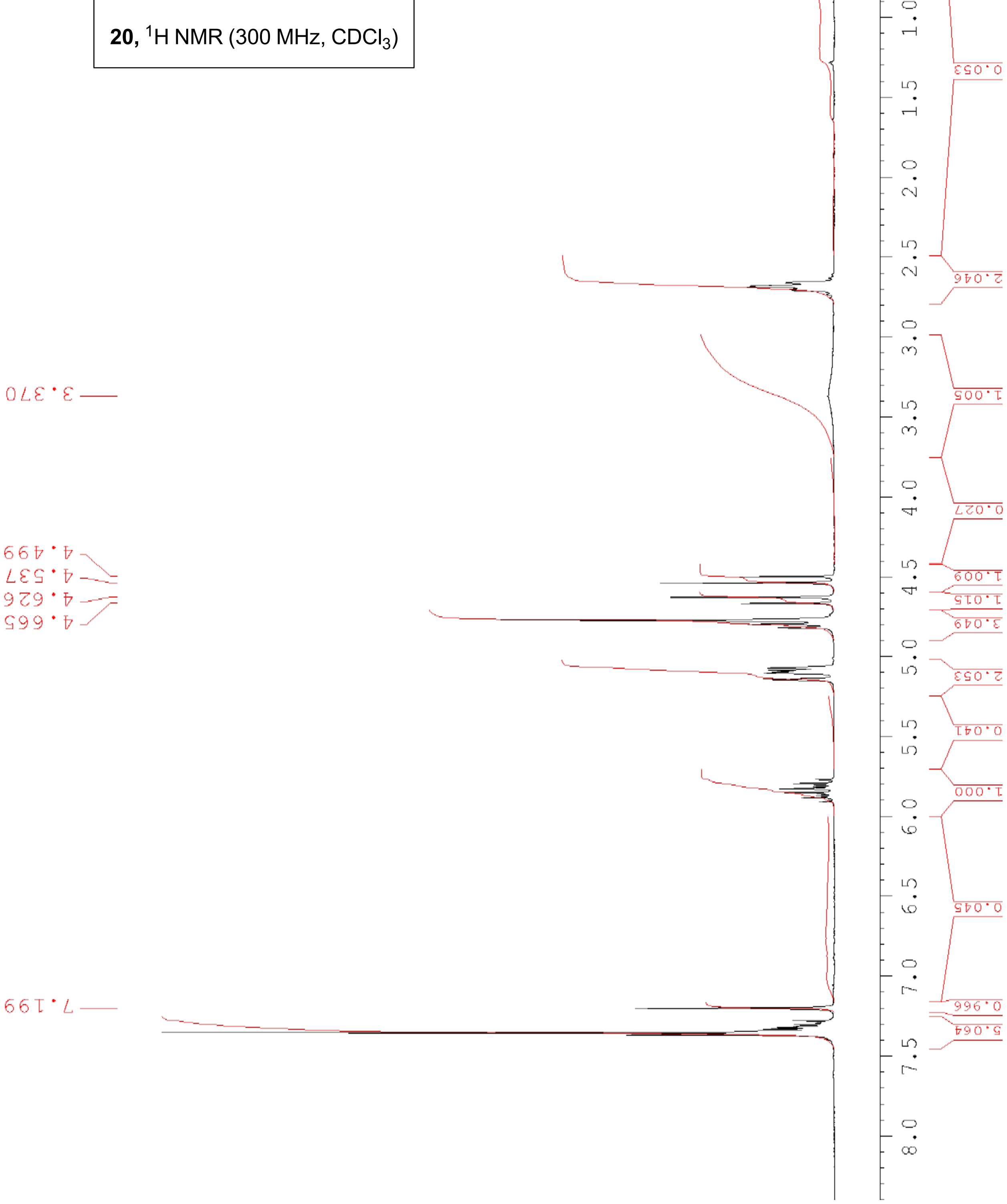

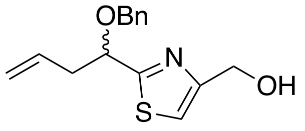


**20,** $^{13}C\{^{1}H\}$ NMR (75 MHz, $CDCl_3$)

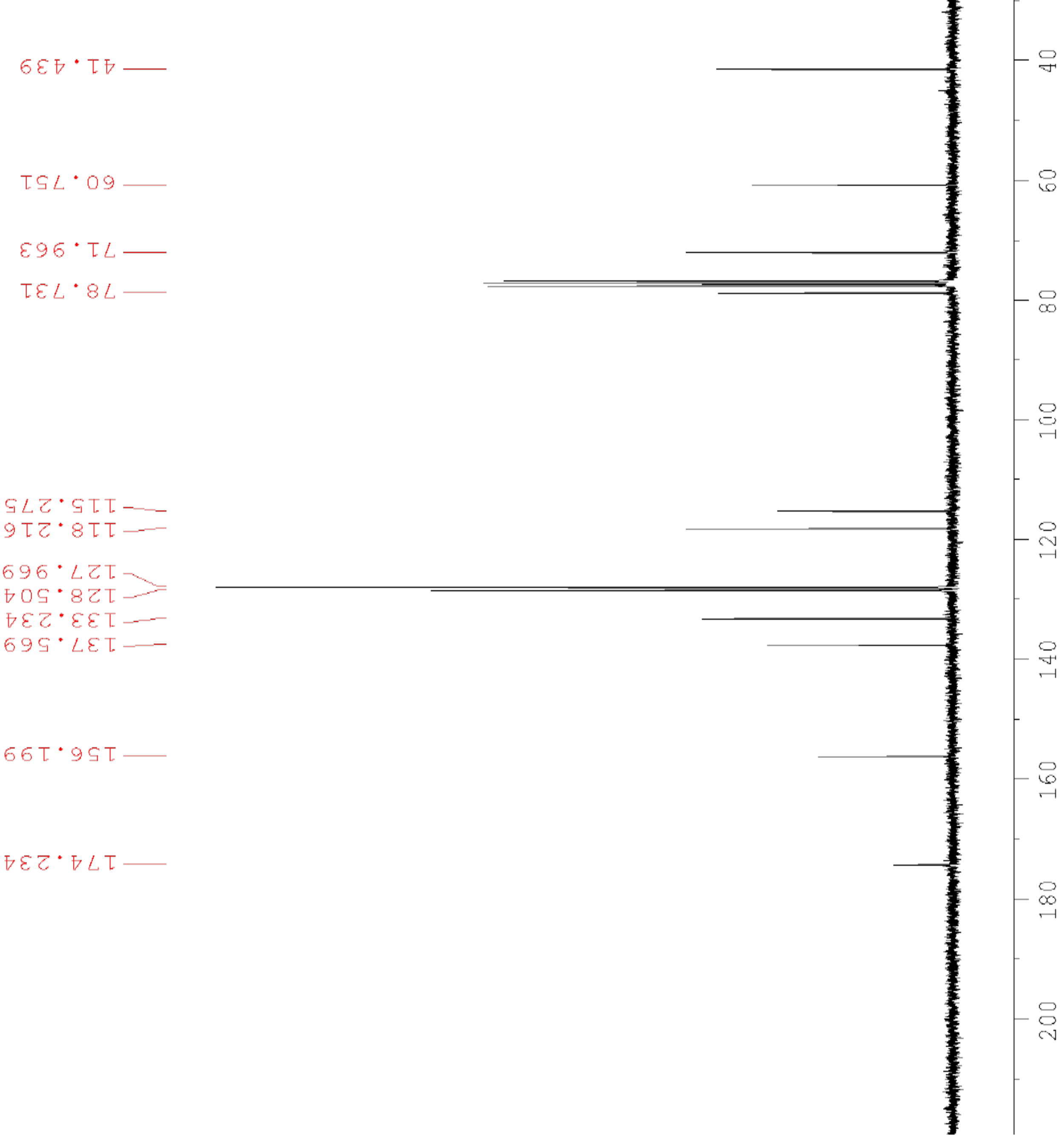

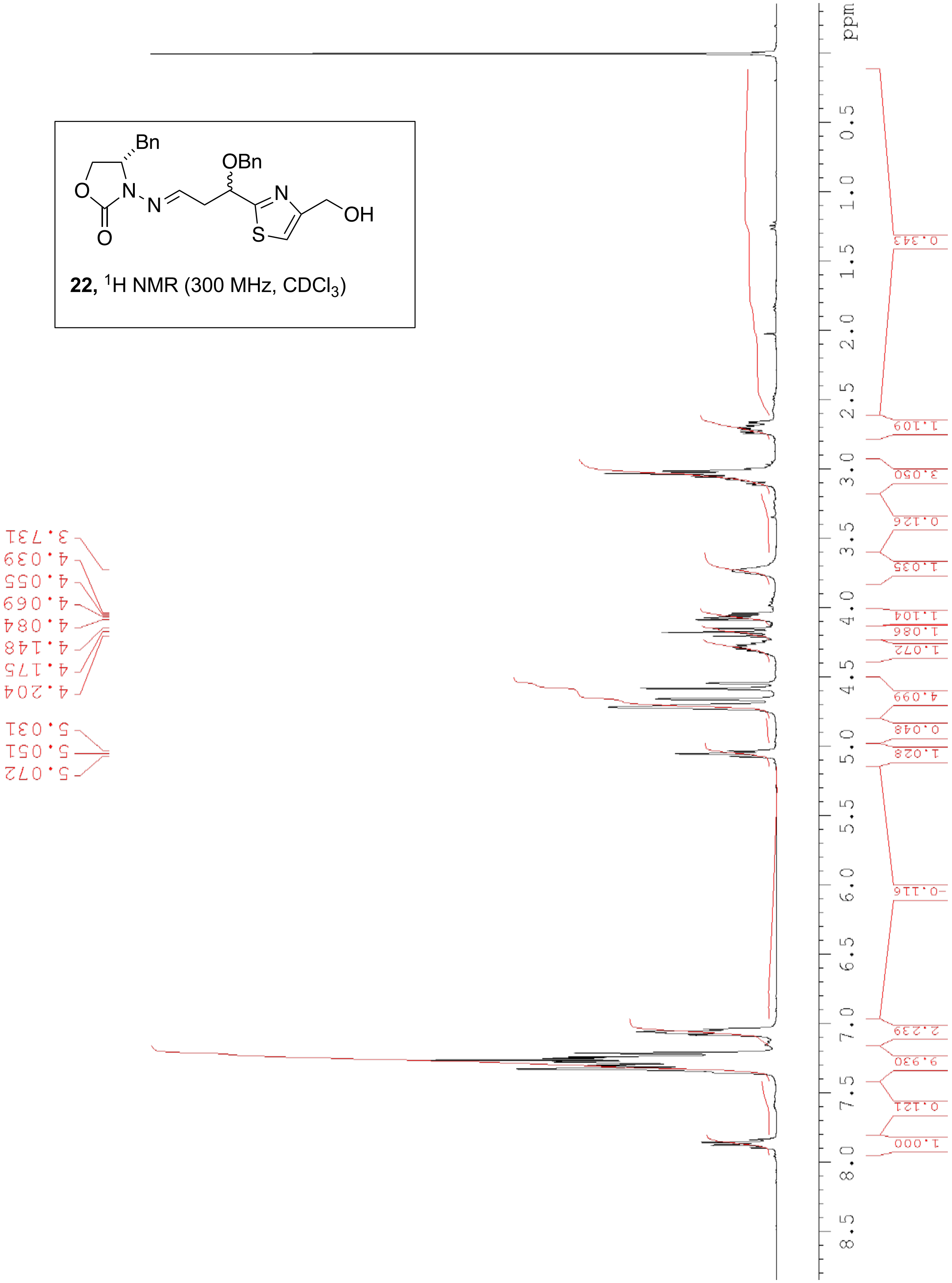


**22,** $^{1}H$ NMR (300 MHz, $CDCl_3$)

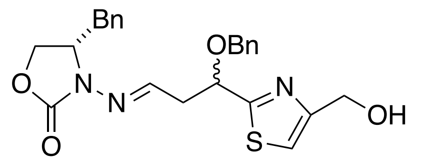


**22,** $^{13}C\{^1H\}$ NMR (75 MHz, $CDCl_3$)

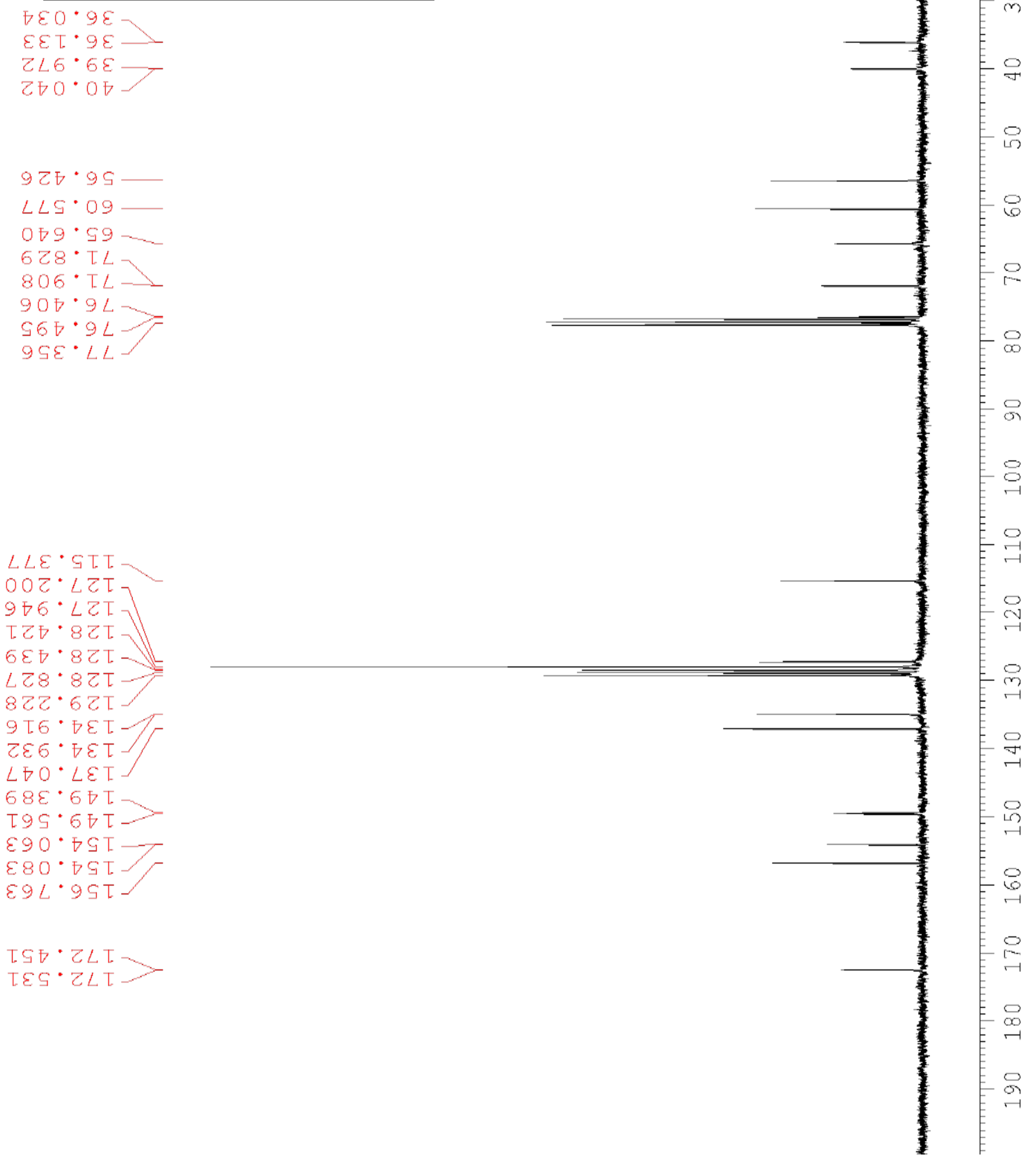

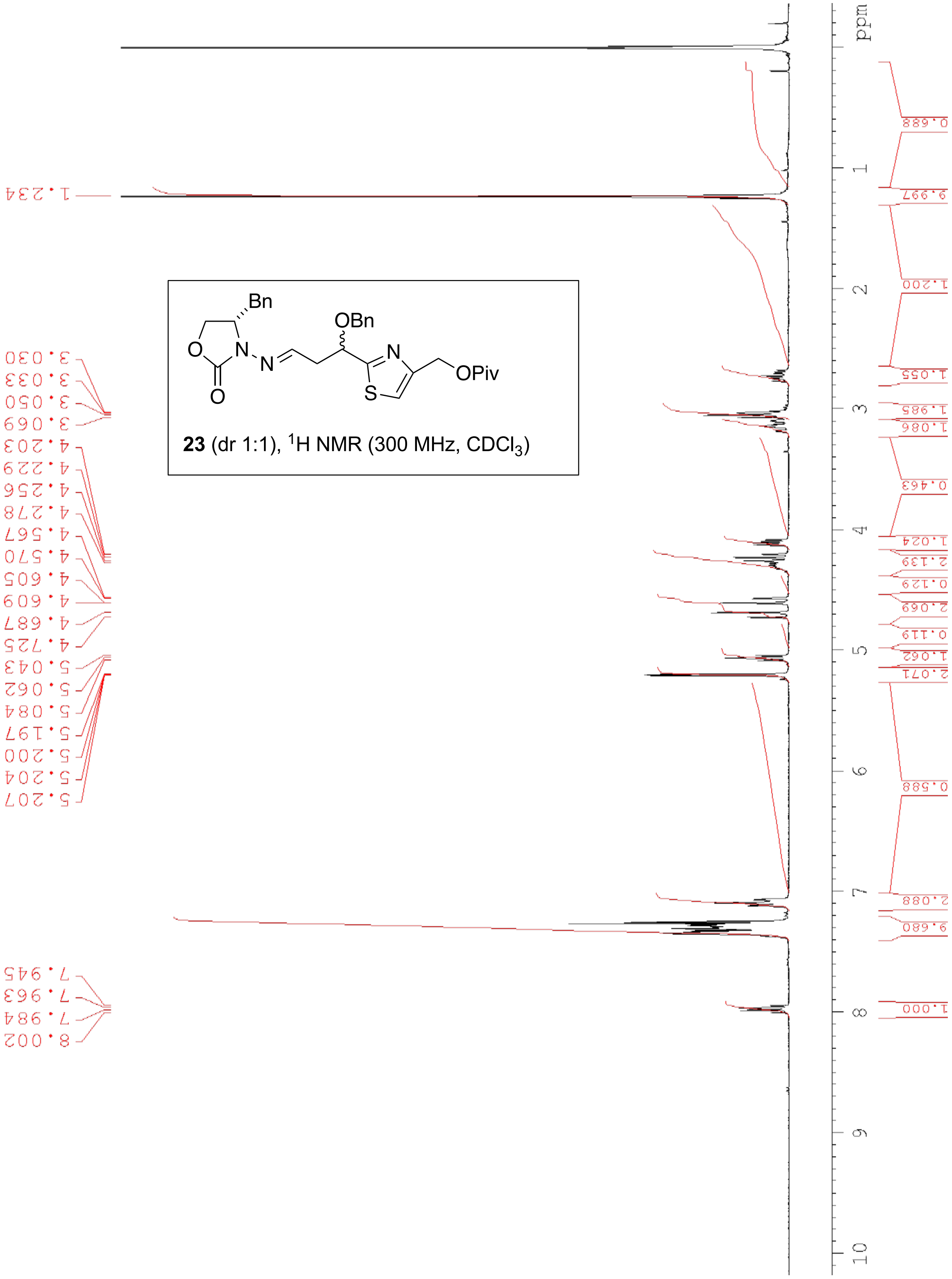


**23** (dr 1:1), $^{1}$H NMR (300 MHz, $CDCl_3$)

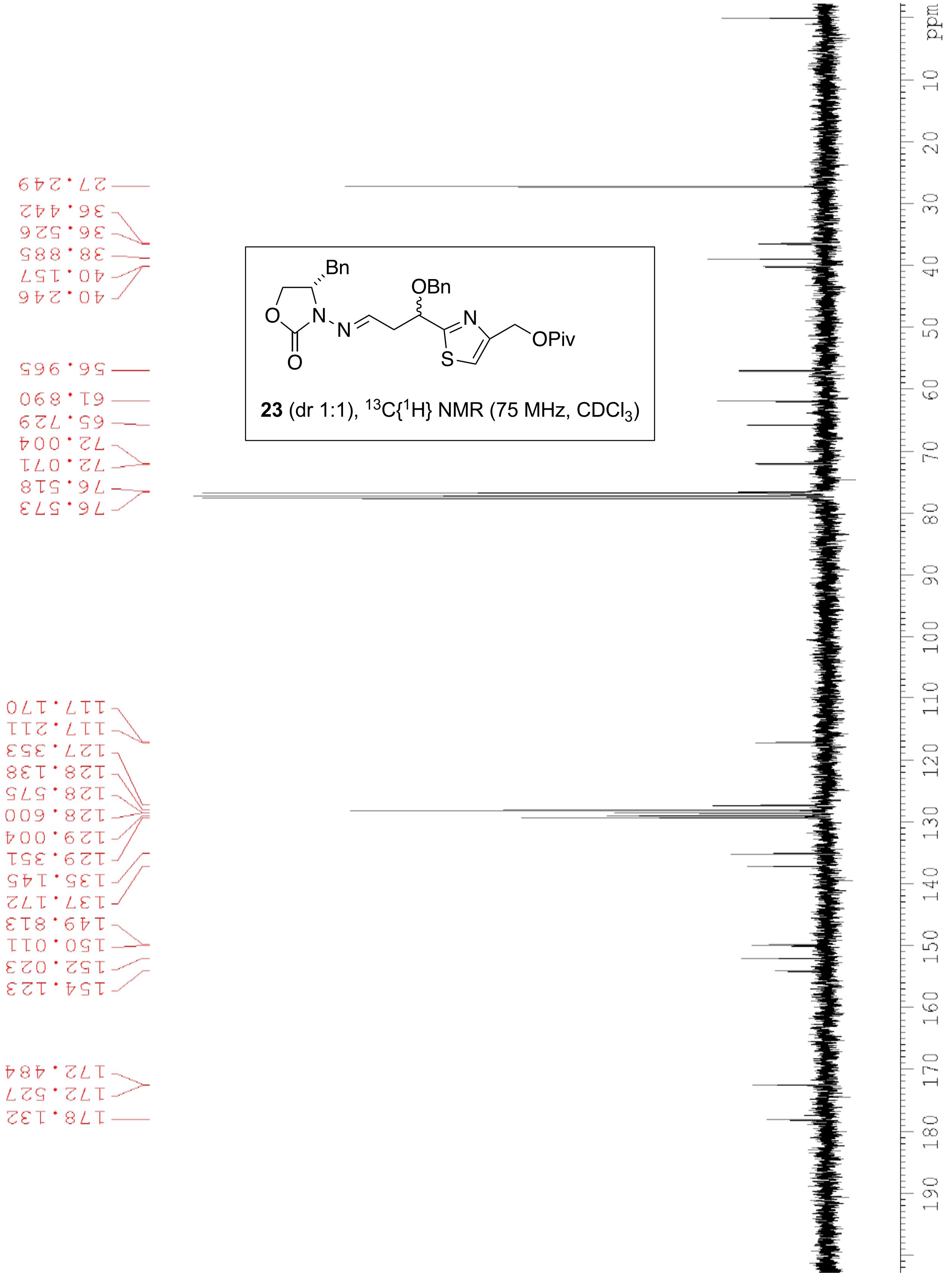


**23** (dr 1:1), $^{13}C\{^{1}H\}$ NMR (75 MHz, $CDCl_3$)

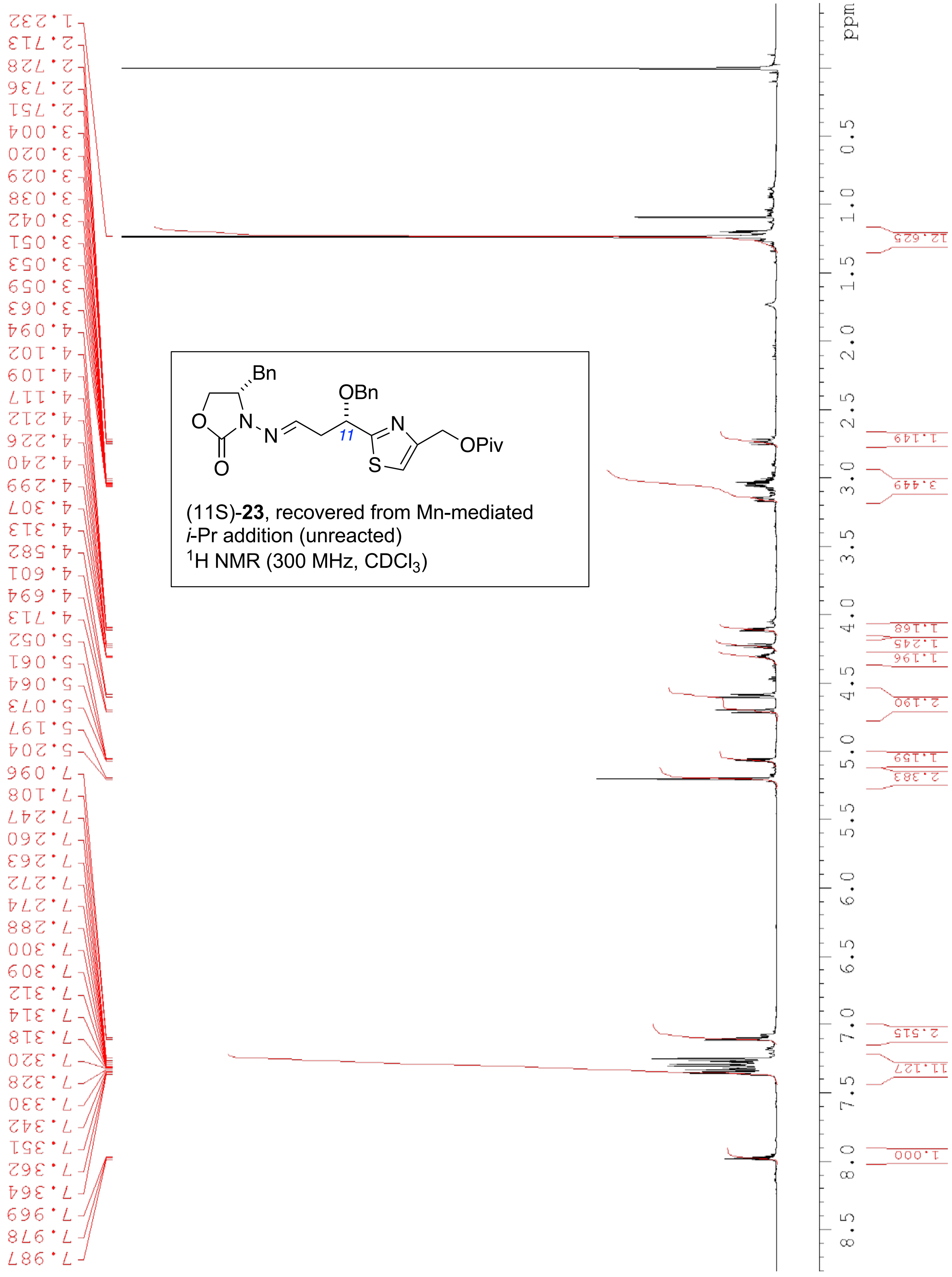
Bn
OBn
O
N
N
11
N
S
OPiv
O
(11S)-23, recovered from Mn-mediated
i-Pr addition (unreacted)
1H NMR (300 MHz, CDCl3)
ppm

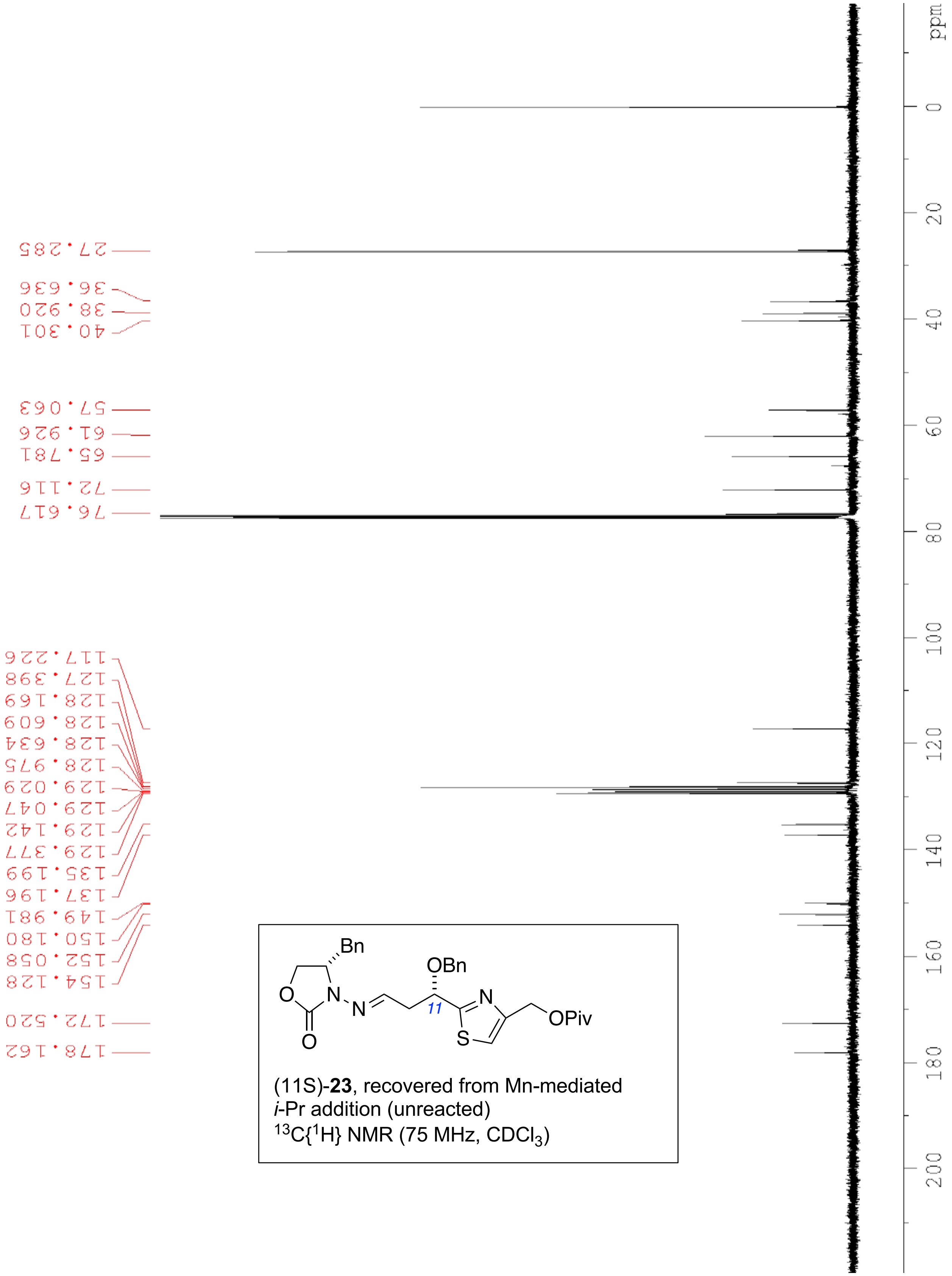


(11S)-**23**, recovered from Mn-mediated *i*-Pr addition (unreacted)
$^{13}C\{^1H\}$ NMR (75 MHz, $CDCl_3$)

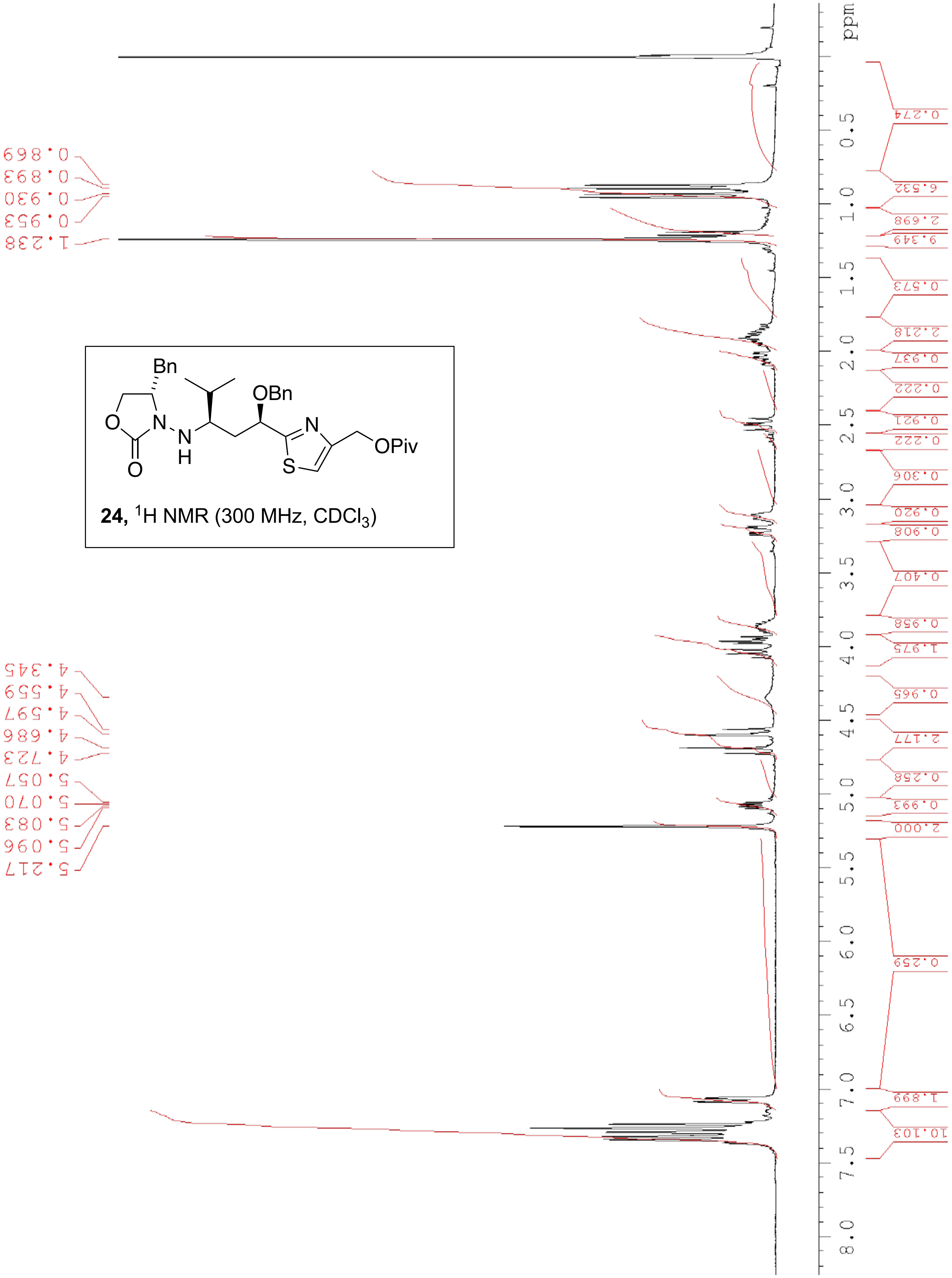


**24,** $^{1}$H NMR (300 MHz, $CDCl_3$)

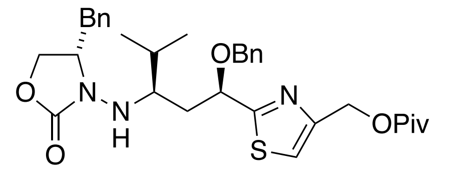


**24,** $^{13}C\{^{1}H\}$ NMR (75 MHz, $CDCl_3$)

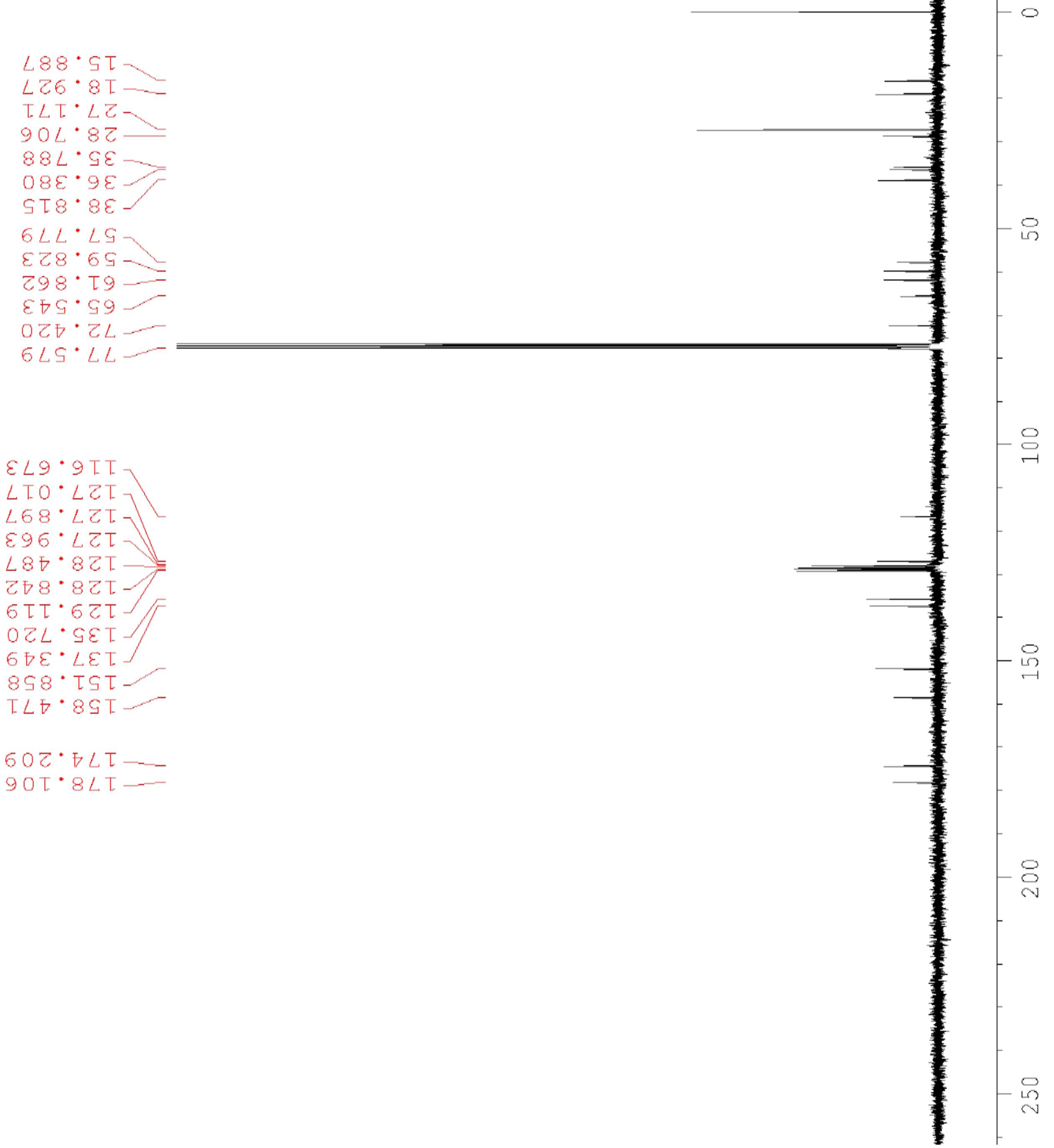

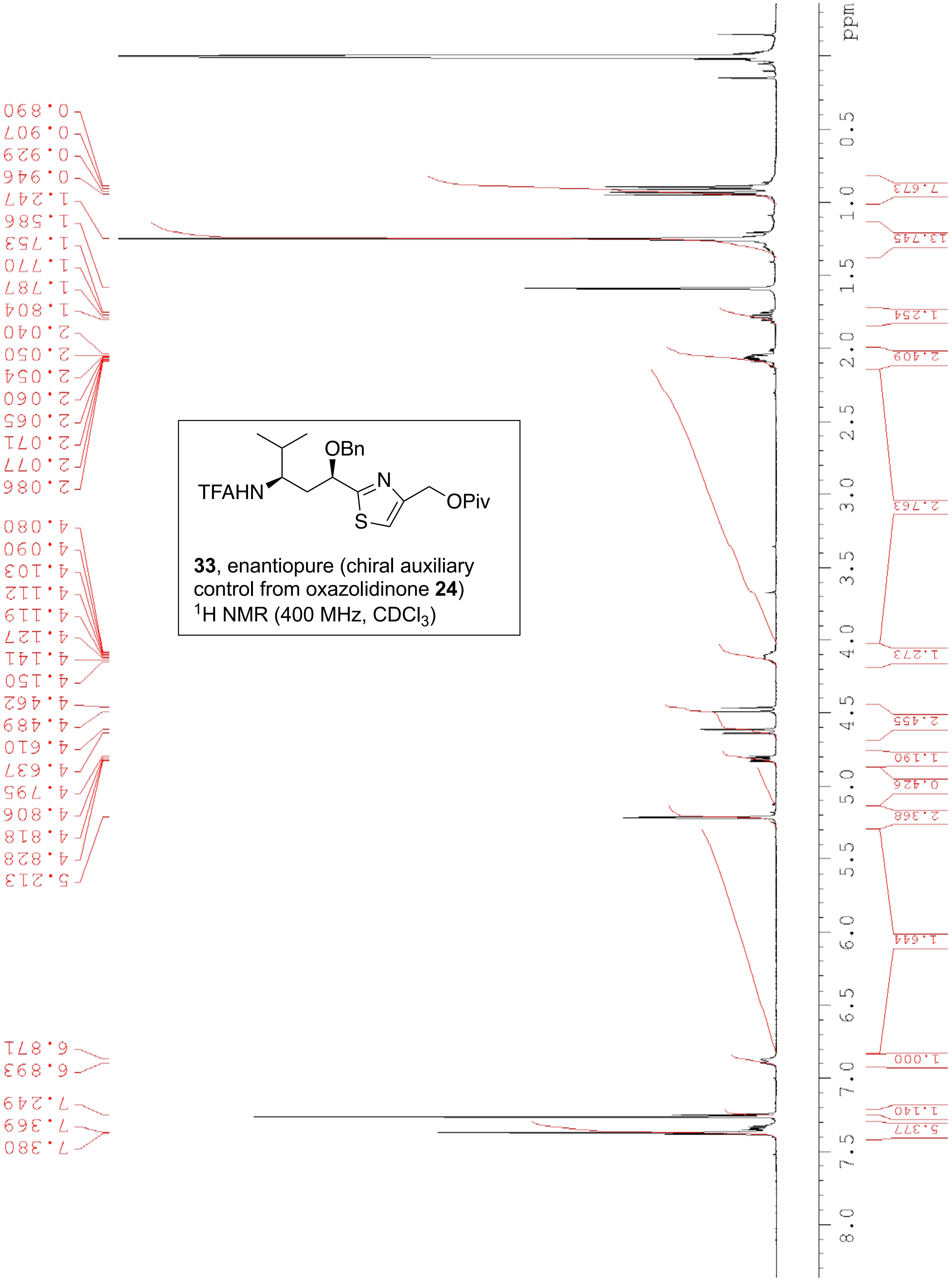


**33**, enantiopure (chiral auxiliary control from oxazolidinone **24**)
$^{1}$H NMR (400 MHz, $CDCl_3$)

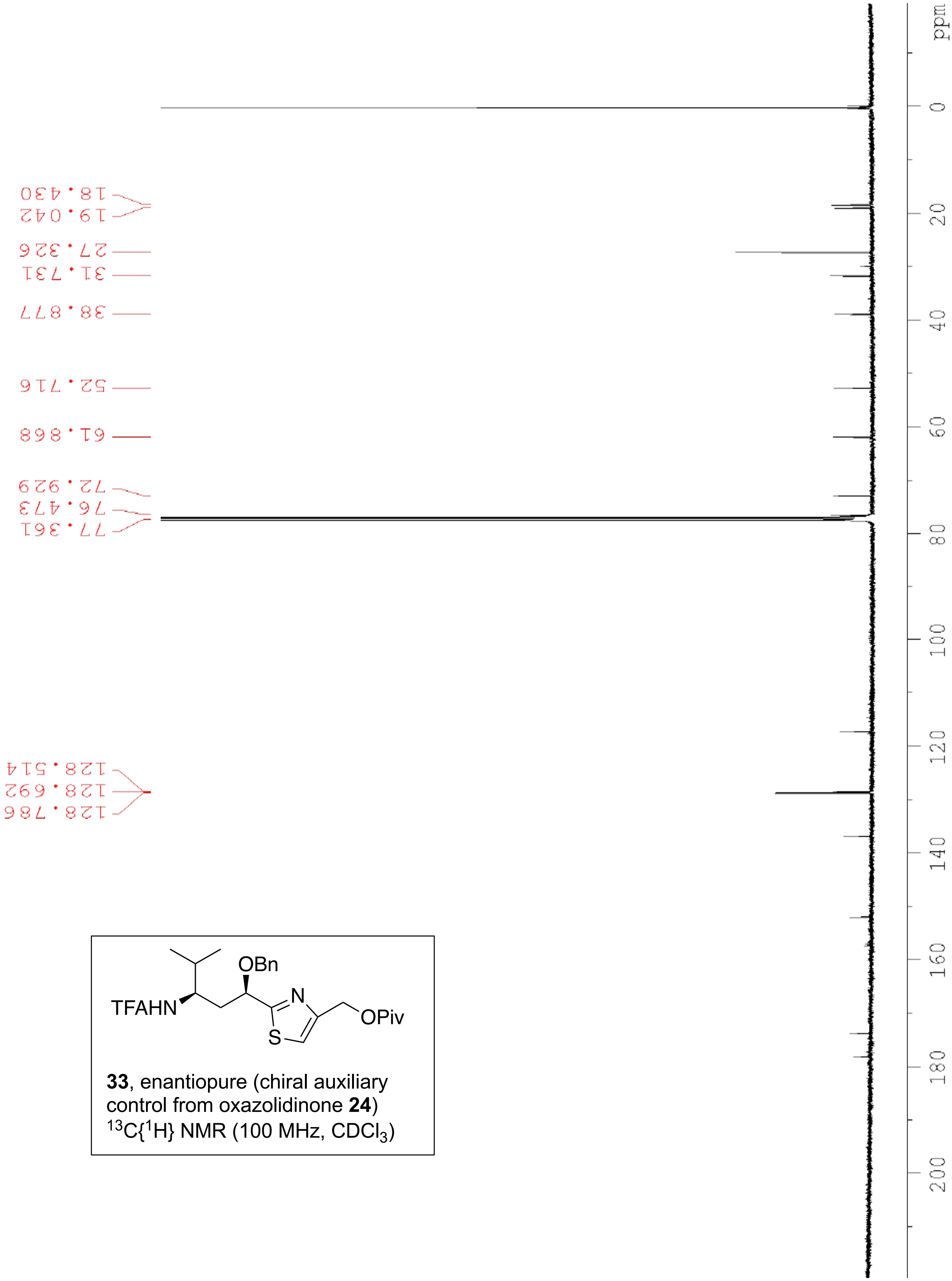


**33**, enantiopure (chiral auxiliary control from oxazolidinone **24**)
$^{13}C\{^{1}H\}$ NMR (100 MHz, $CDCl_3$)

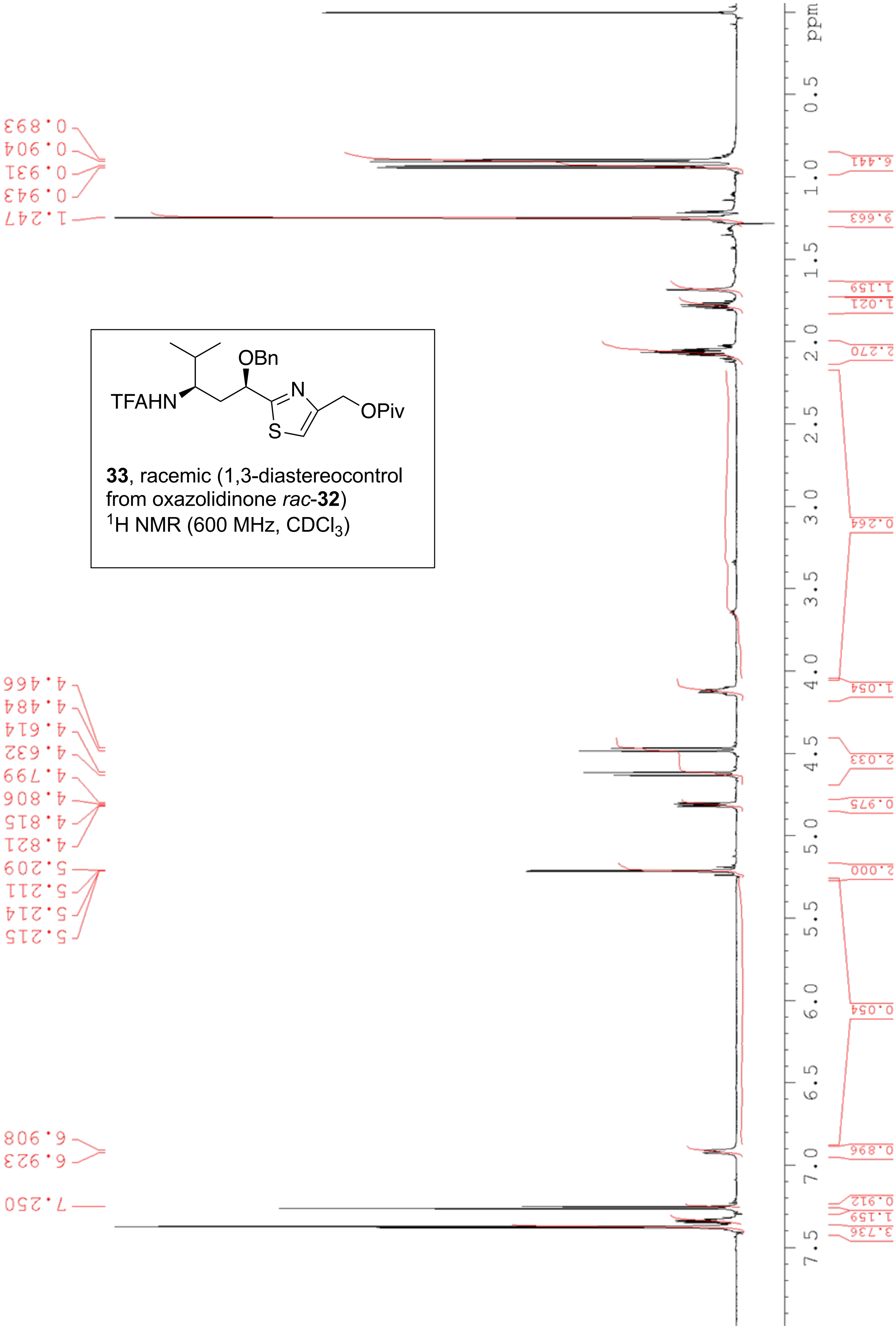


**33**, racemic (1,3-diastereocontrol from oxazolidinone *rac*-**32**)
$^{1}$H NMR (600 MHz, $CDCl_3$)

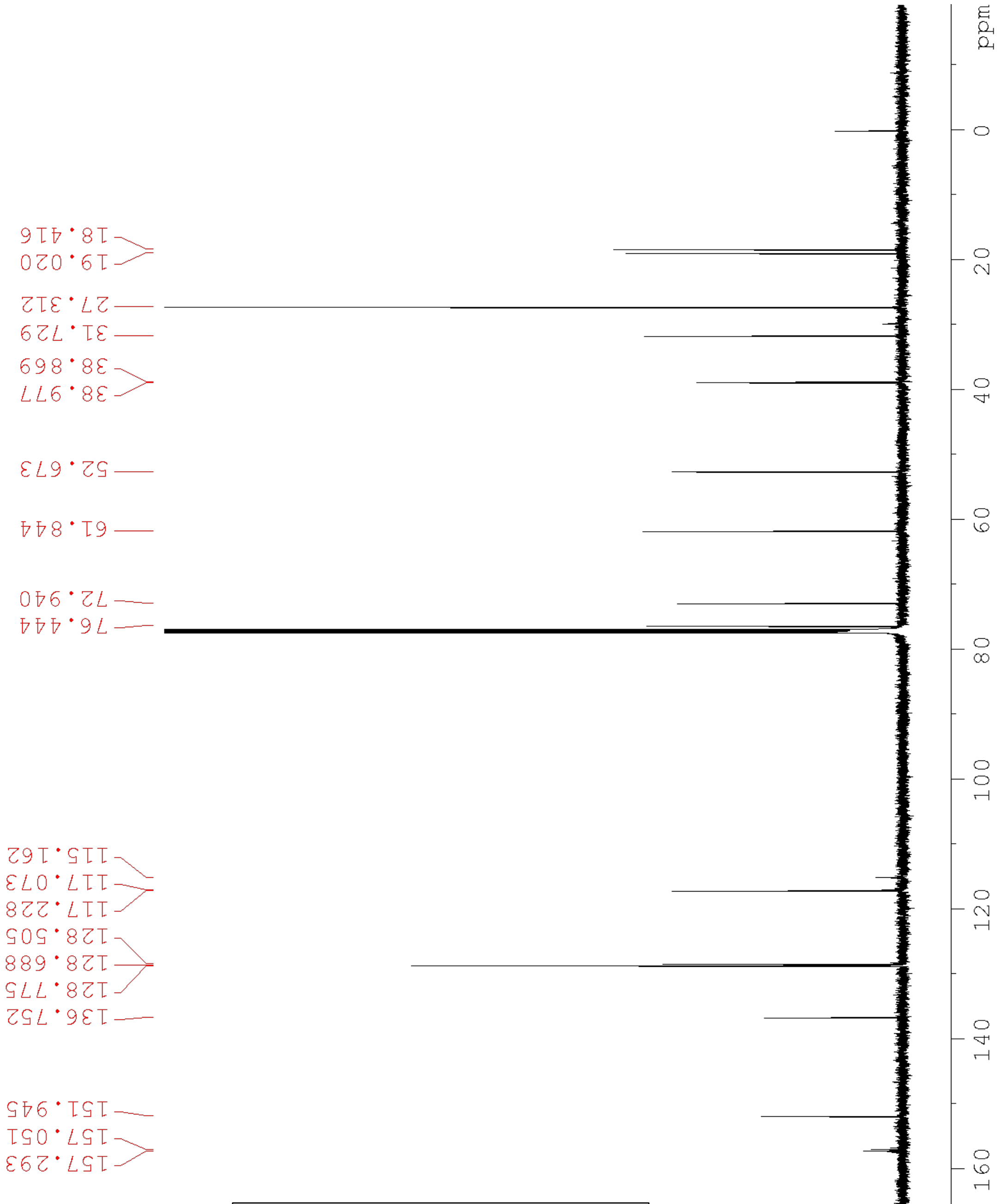


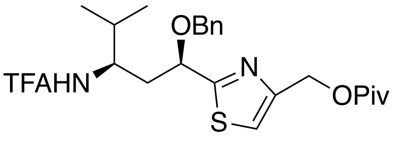


**33**, racemic (1,3-diastereocontrol from oxazolidinone *rac*-**32**)
$^{13}C\{^{1}H\}$ NMR (150 MHz, $CDCl_3$)

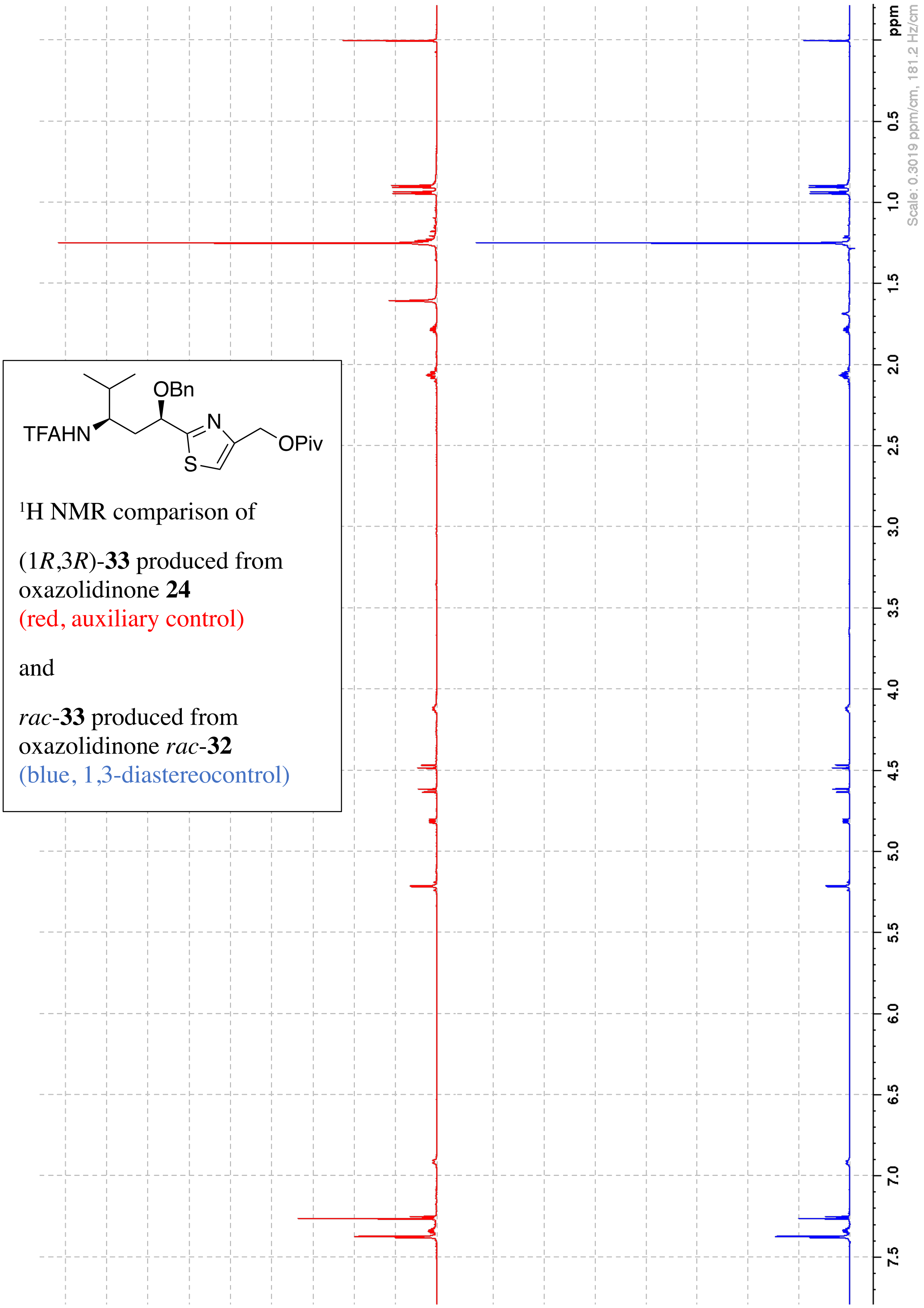


$^1$H NMR comparison of

(1*R*,3*R*)-**33** produced from oxazolidinone **24**
(red, auxiliary control)

and

*rac*-**33** produced from oxazolidinone *rac*-**32**
(blue, 1,3-diastereocontrol)

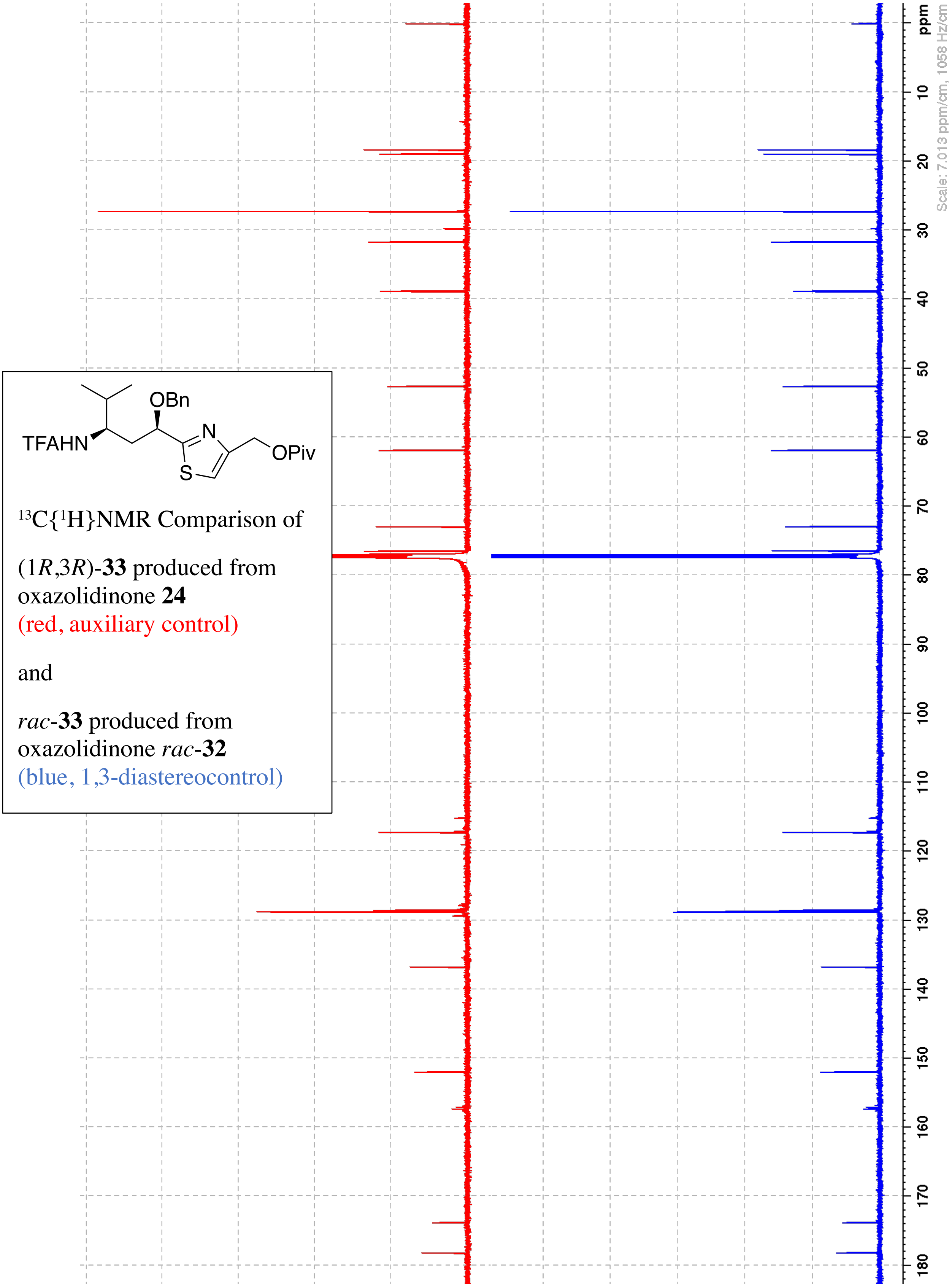


$^{13}C\{^{1}H\}$NMR Comparison of

(1*R*,3*R*)-**33** produced from oxazolidinone **24**
(red, auxiliary control)

and

*rac*-**33** produced from oxazolidinone *rac*-**32**
(blue, 1,3-diastereocontrol)

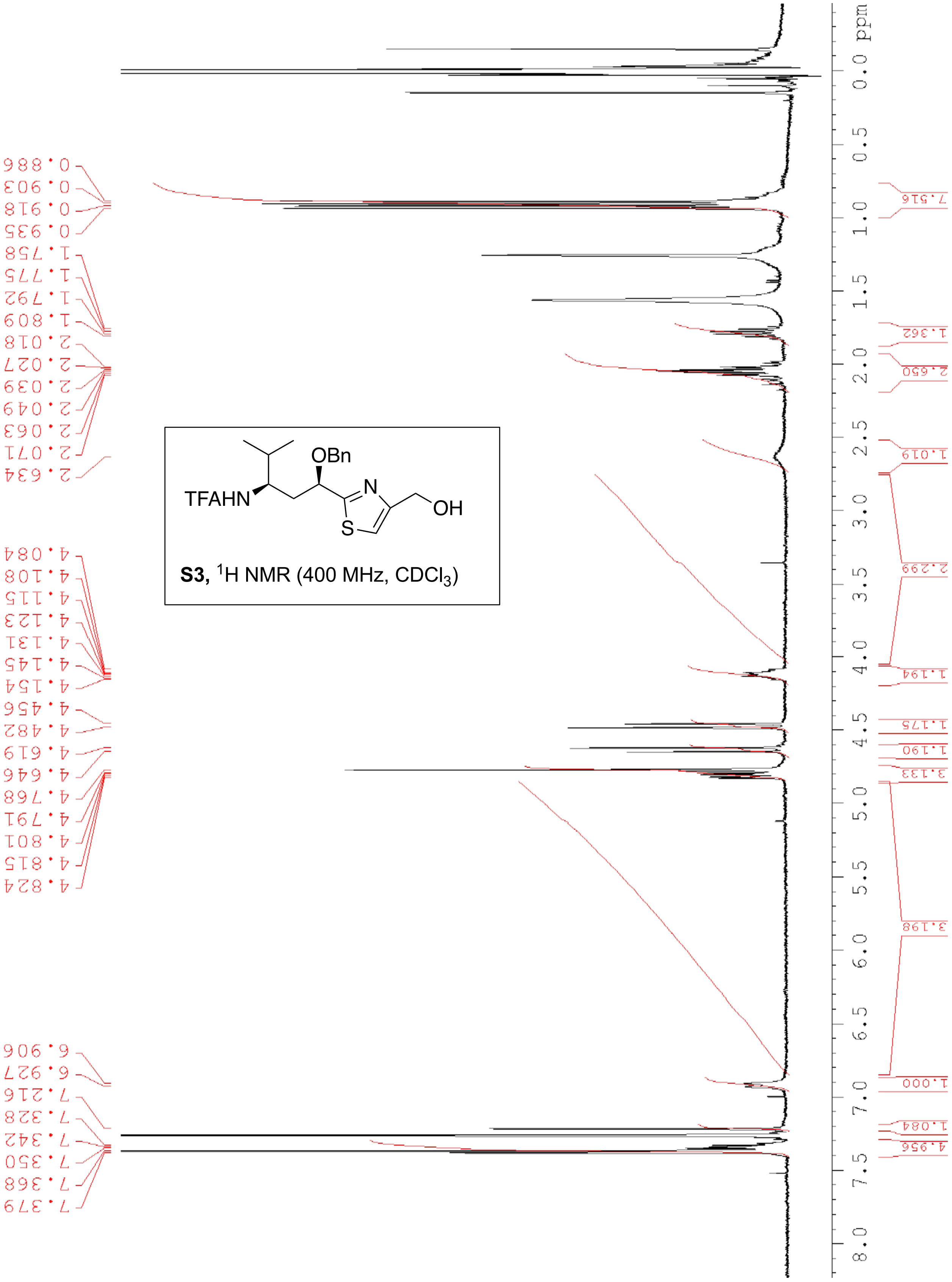


**S3,** ¹H NMR (400 MHz, $CDCl_3$)

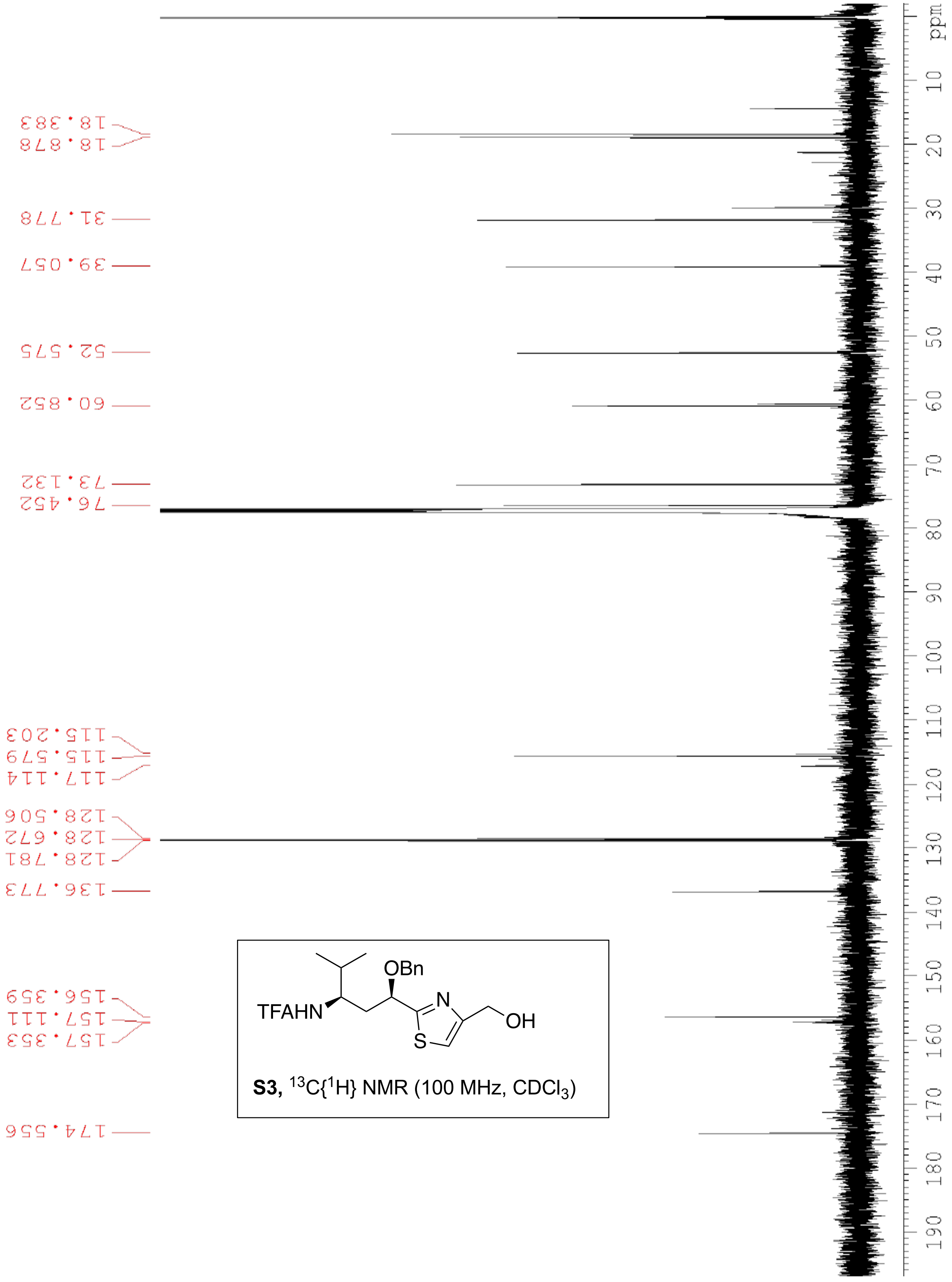


**S3,** $^{13}C\{^{1}H\}$ NMR (100 MHz, $CDCl_3$)

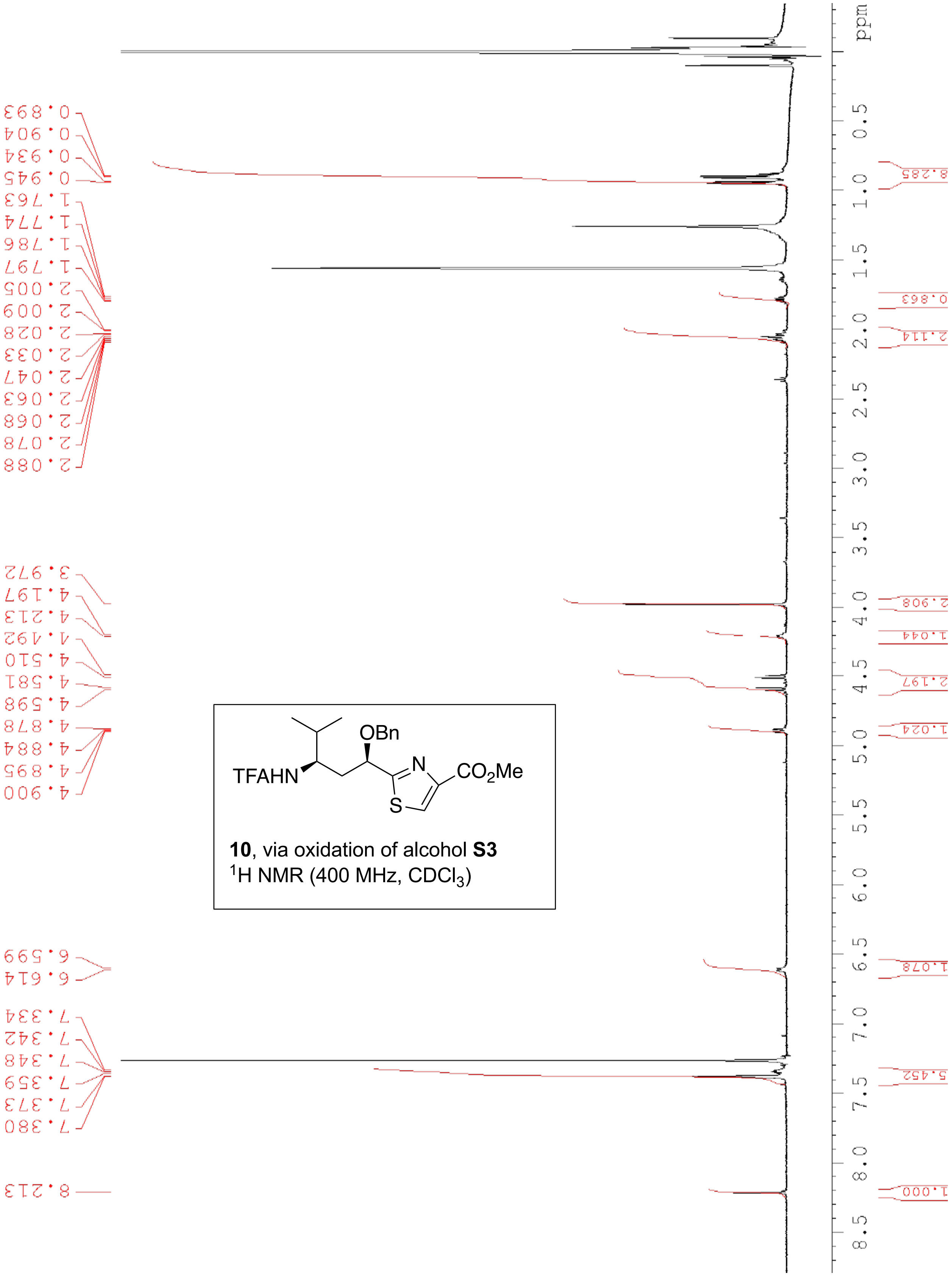


**10**, via oxidation of alcohol **S3**
$^1$H NMR (400 MHz, $CDCl_3$)

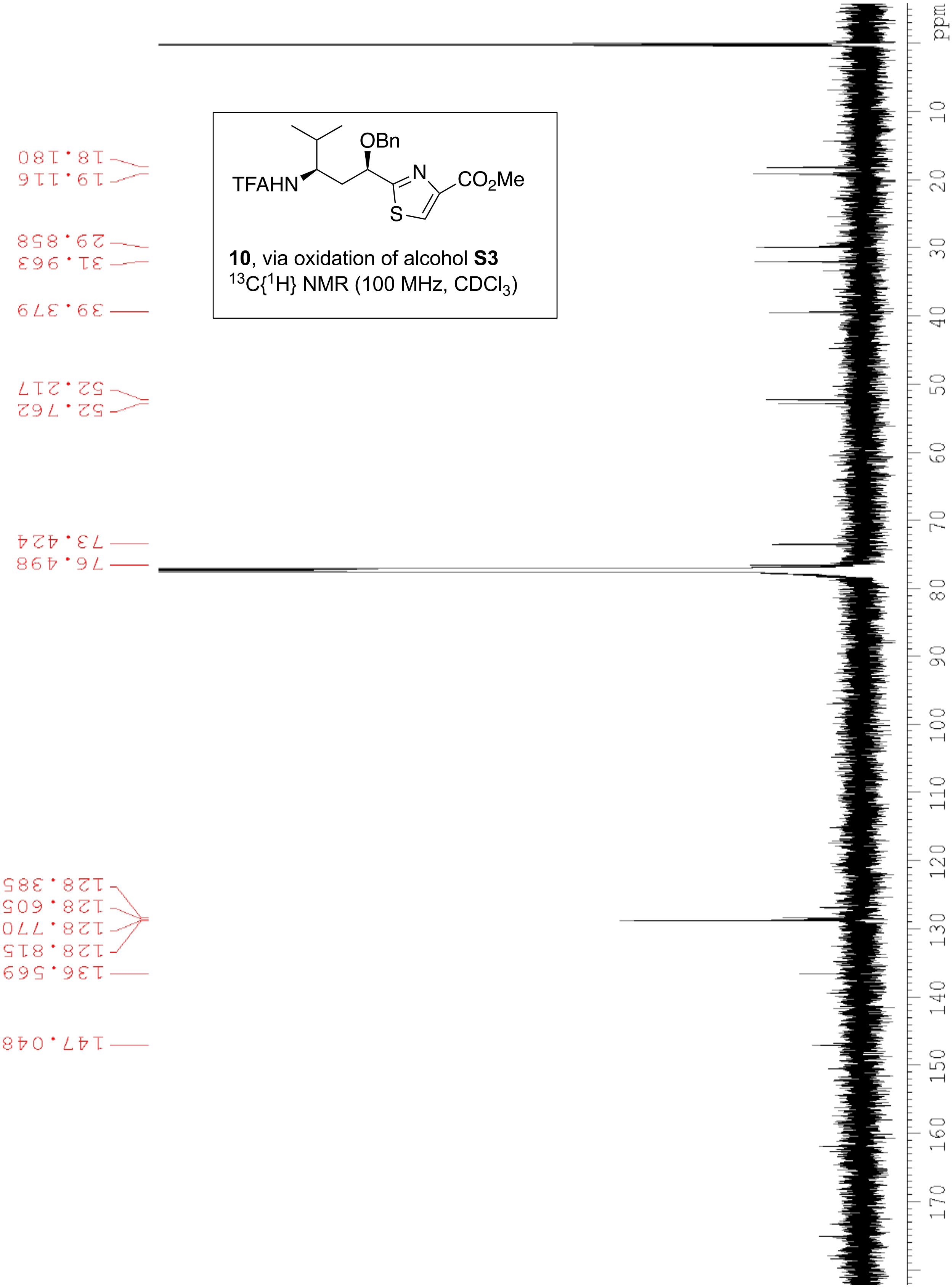


**10**, via oxidation of alcohol **S3**
$^{13}C\{^{1}H\}$ NMR (100 MHz, $CDCl_3$)

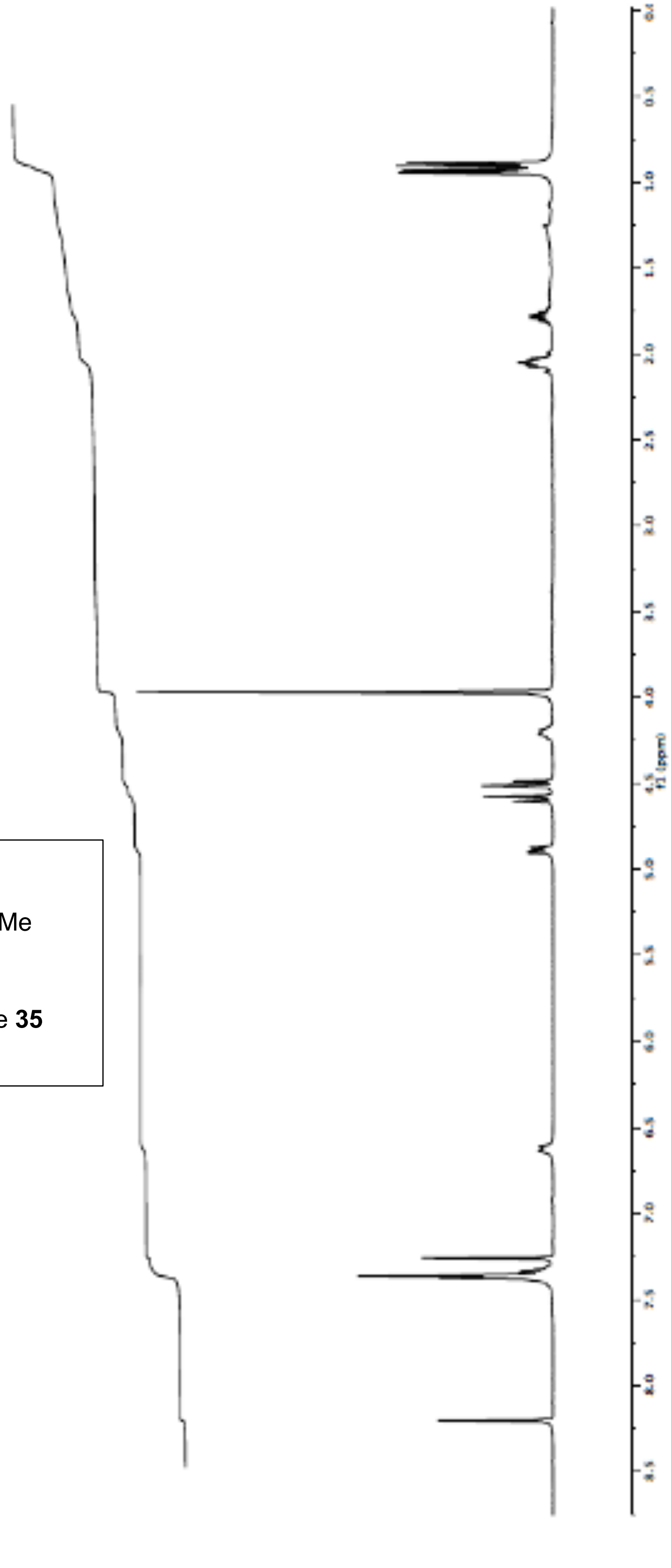

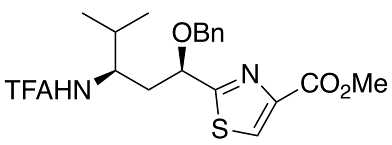


**10**, via oxidation of thiazolidine **35**
$^1$H NMR (400 MHz, $CDCl_3$)

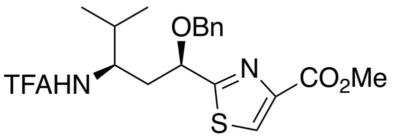


**10**, via oxidation of thiazolidine **35**
$^{13}C\{^{1}H\}$ NMR (400 MHz, $CDCl_3$)

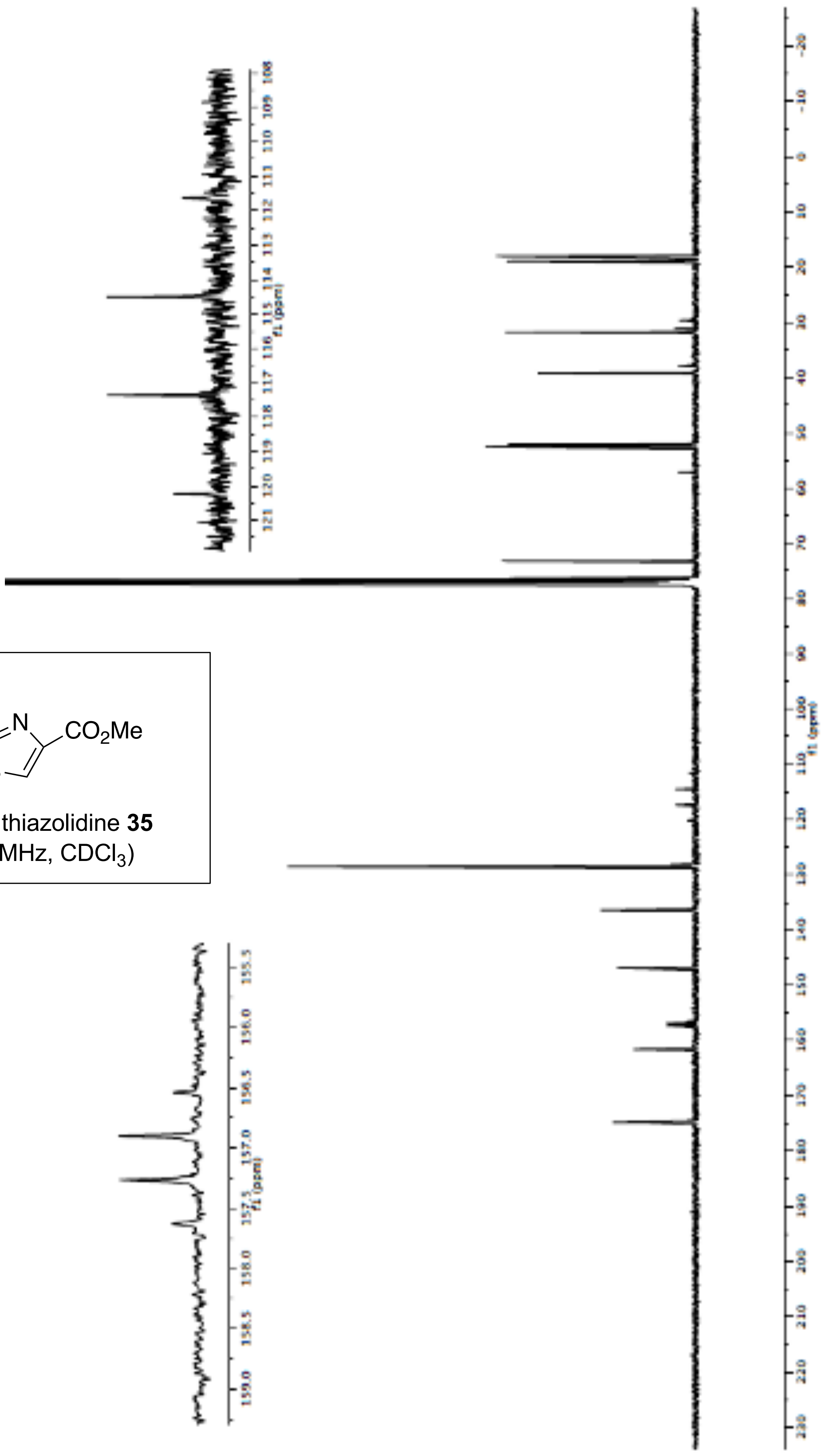

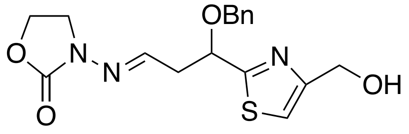


*rac*-**30**, $^{1}$H NMR (600 MHz, $CDCl_3$)

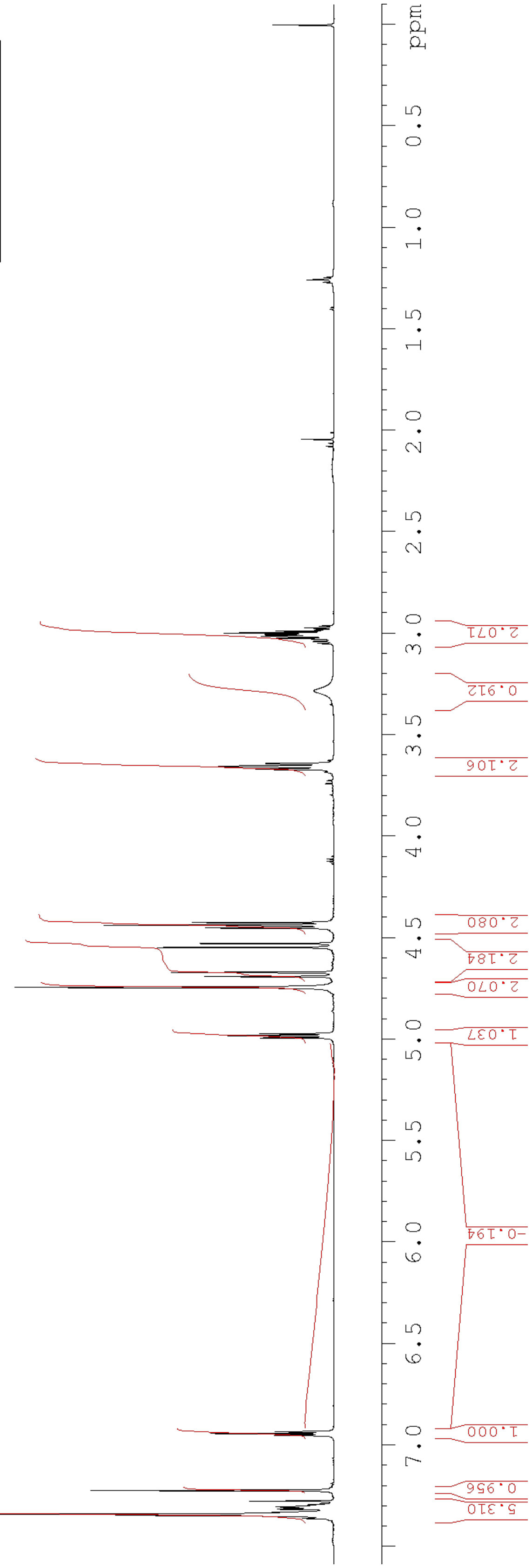

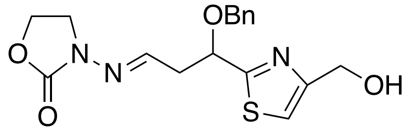


*rac*-**30,** $^{13}C\{^1H\}$ NMR (150 MHz, $CDCl_3$)

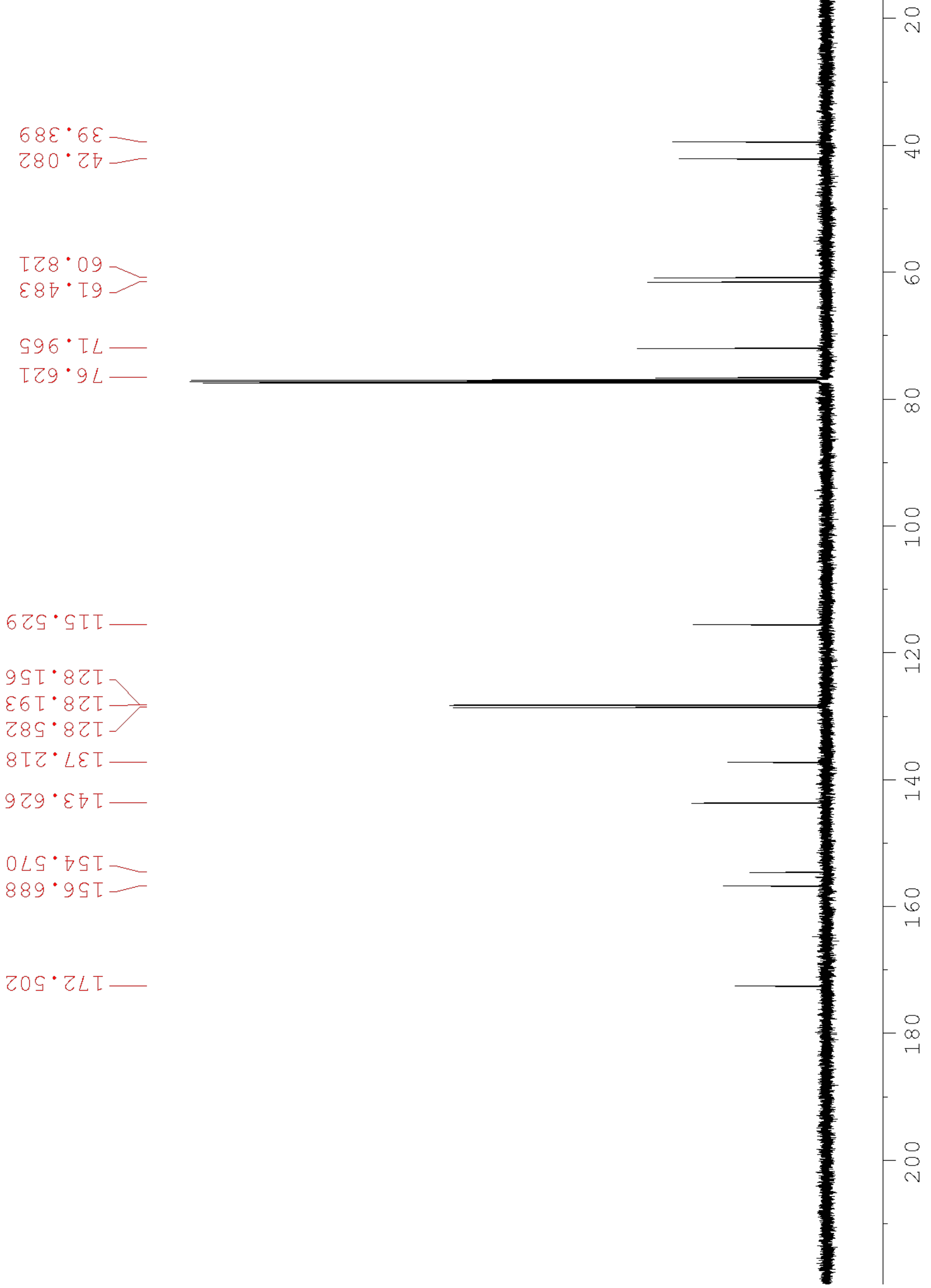

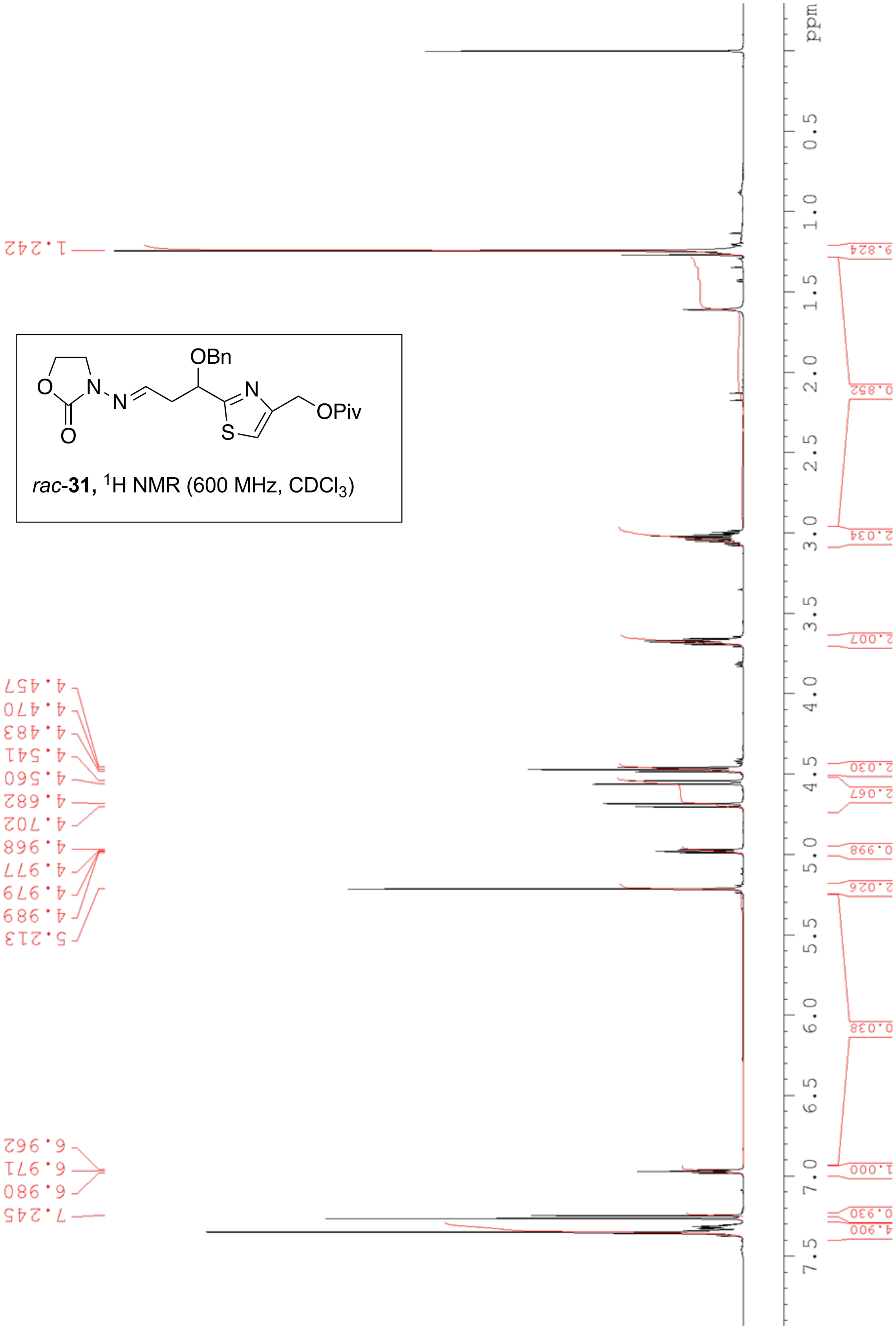


*rac*-**31,** $^{1}$H NMR (600 MHz, $CDCl_3$)

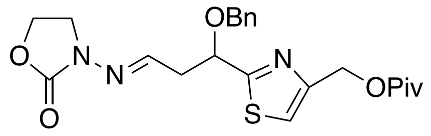


*rac*-**31,** $^{13}C\{^1H\}$ NMR (150 MHz, $CDCl_3$)

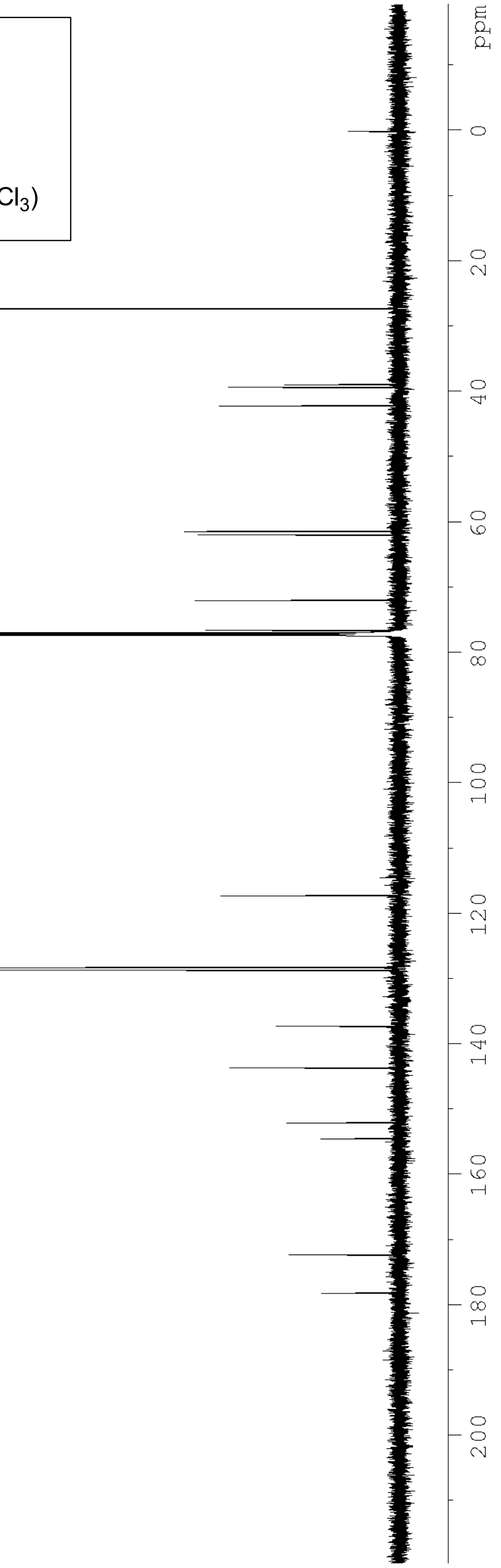

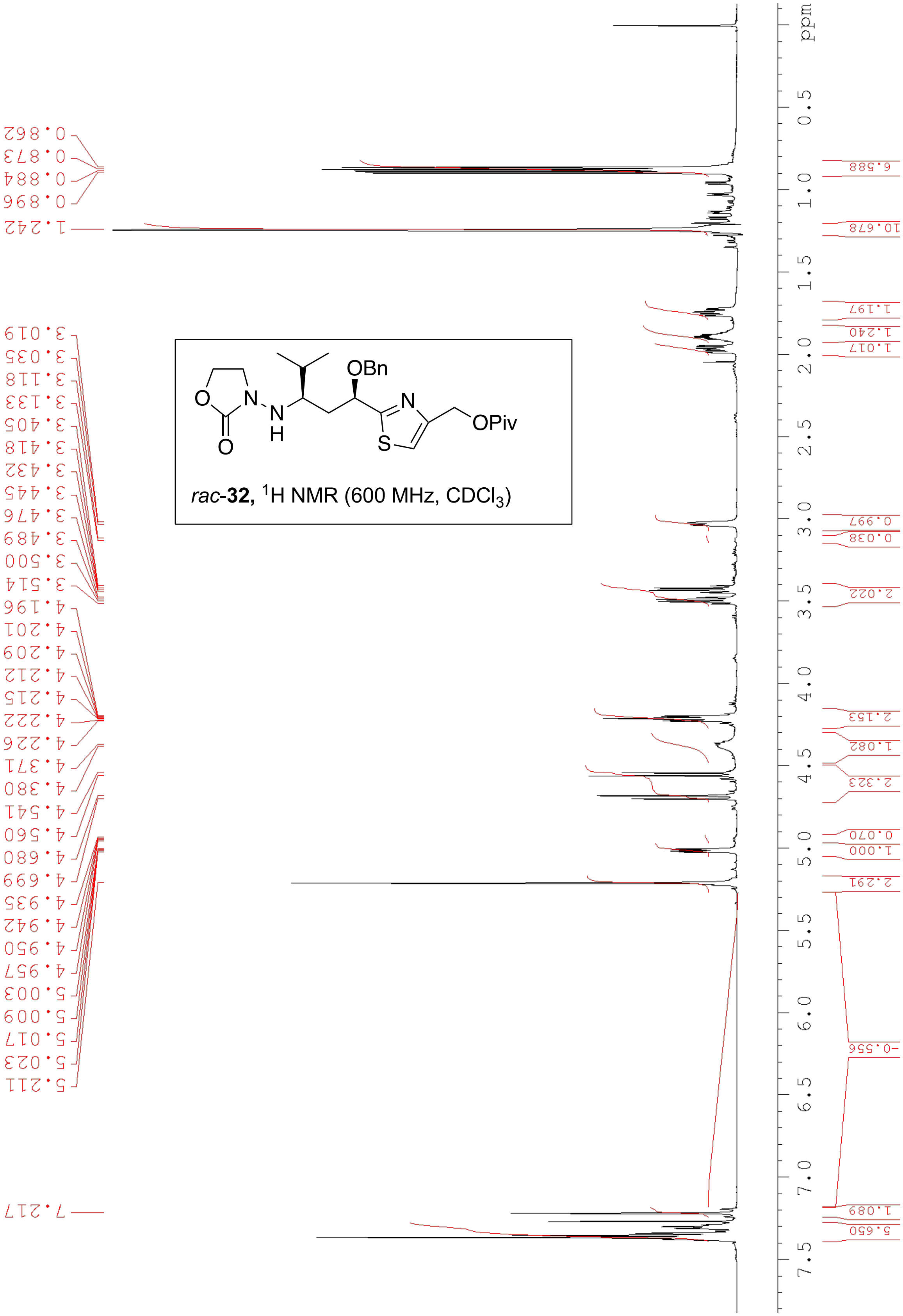


*rac*-**32**, $^1$H NMR (600 MHz, $CDCl_3$)

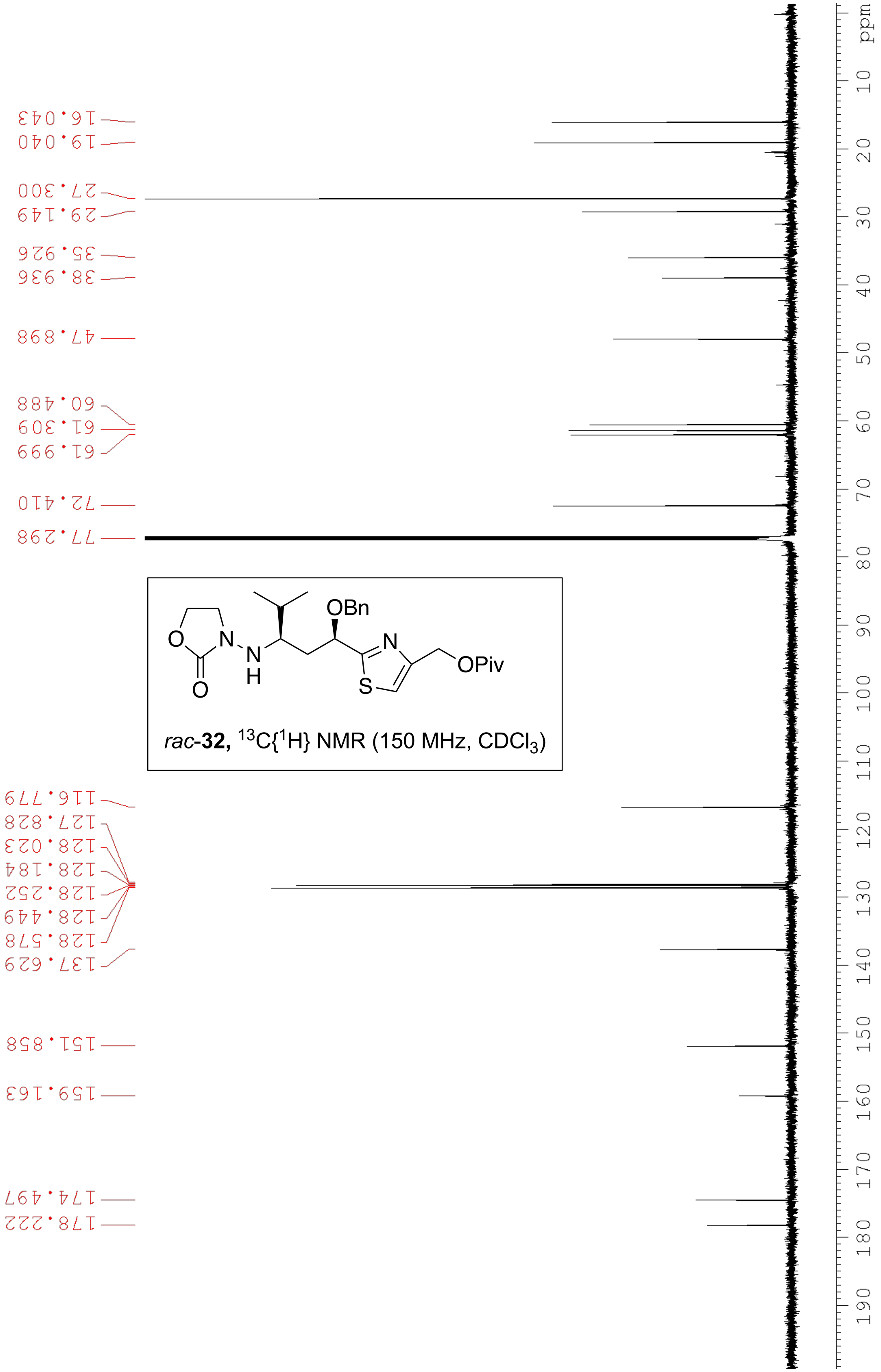
16.043
19.040
27.300
29.149
35.926
38.936
47.898
60.488
61.309
61.999
72.410
77.298
116.779
127.828
128.023
128.184
128.252
128.449
128.578
137.629
151.858
159.163
174.497
178.222
OBn
OPiv
*rac*-**32,** $^{13}C\{^{1}H\}$ NMR (150 MHz, $CDCl_3$)
ppm

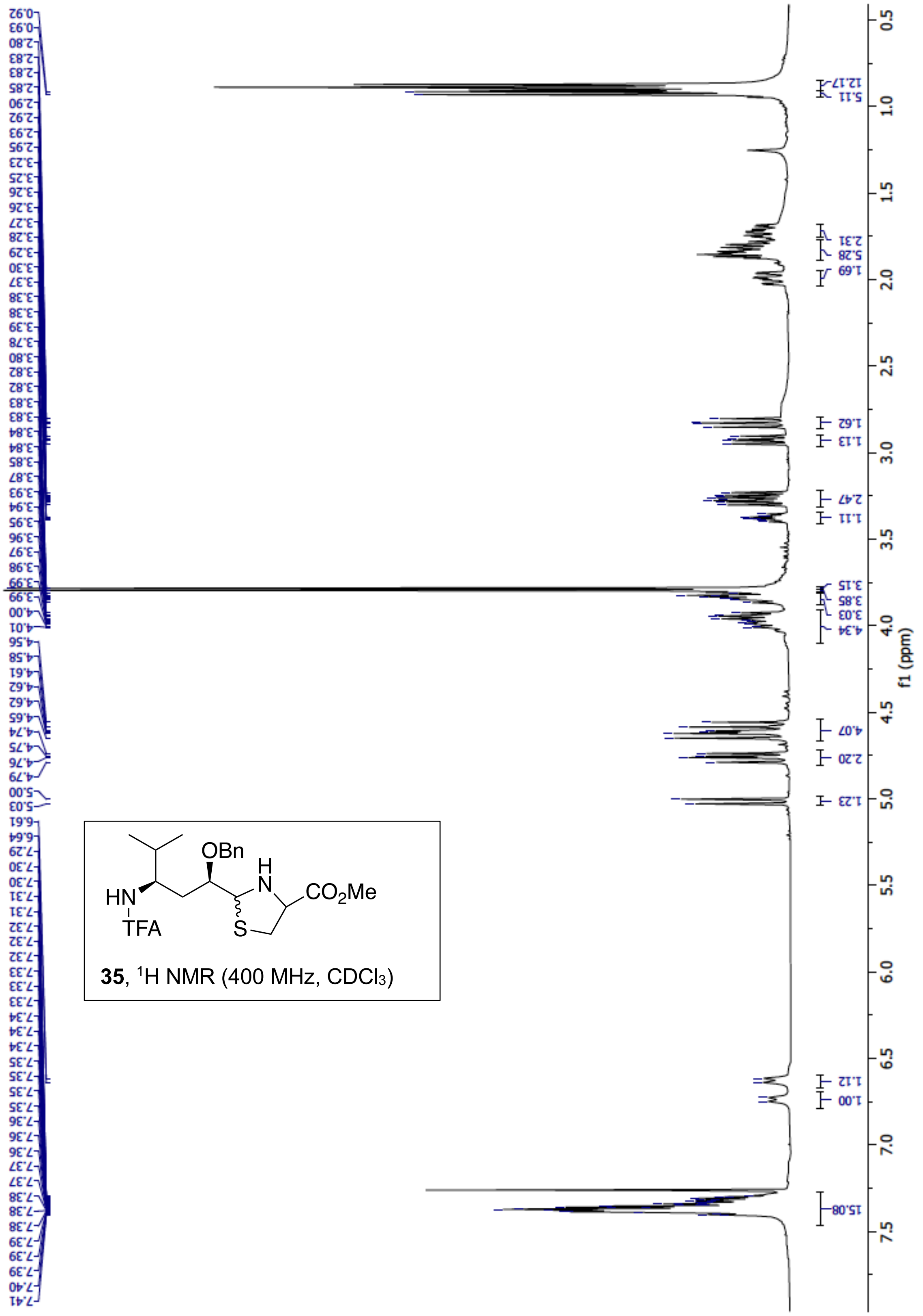


**35**, $^{1}H$ NMR (400 MHz, $CDCl_3$)

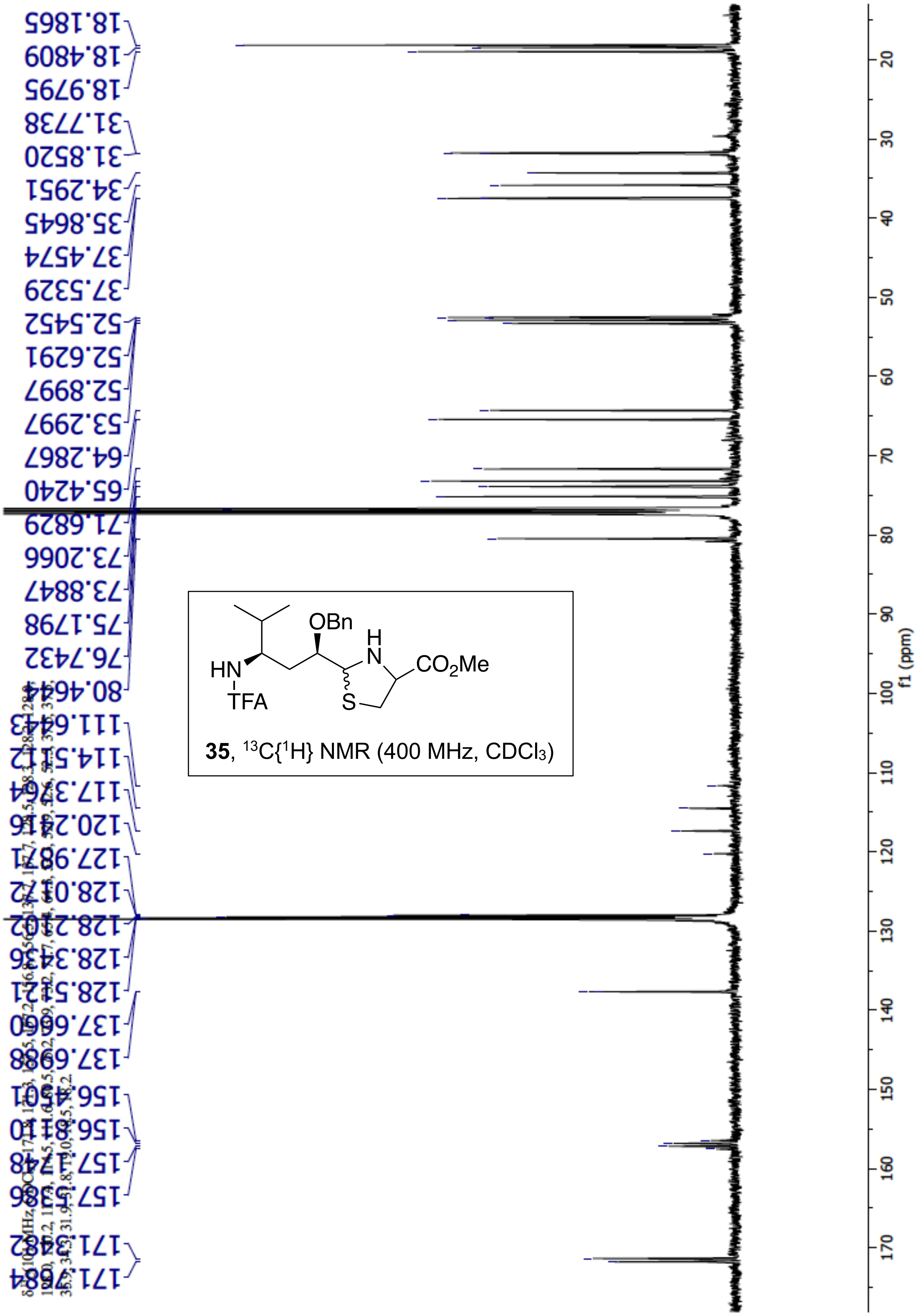


**35**, $^{13}C\{^1H\}$ NMR (400 MHz, $CDCl_3$)

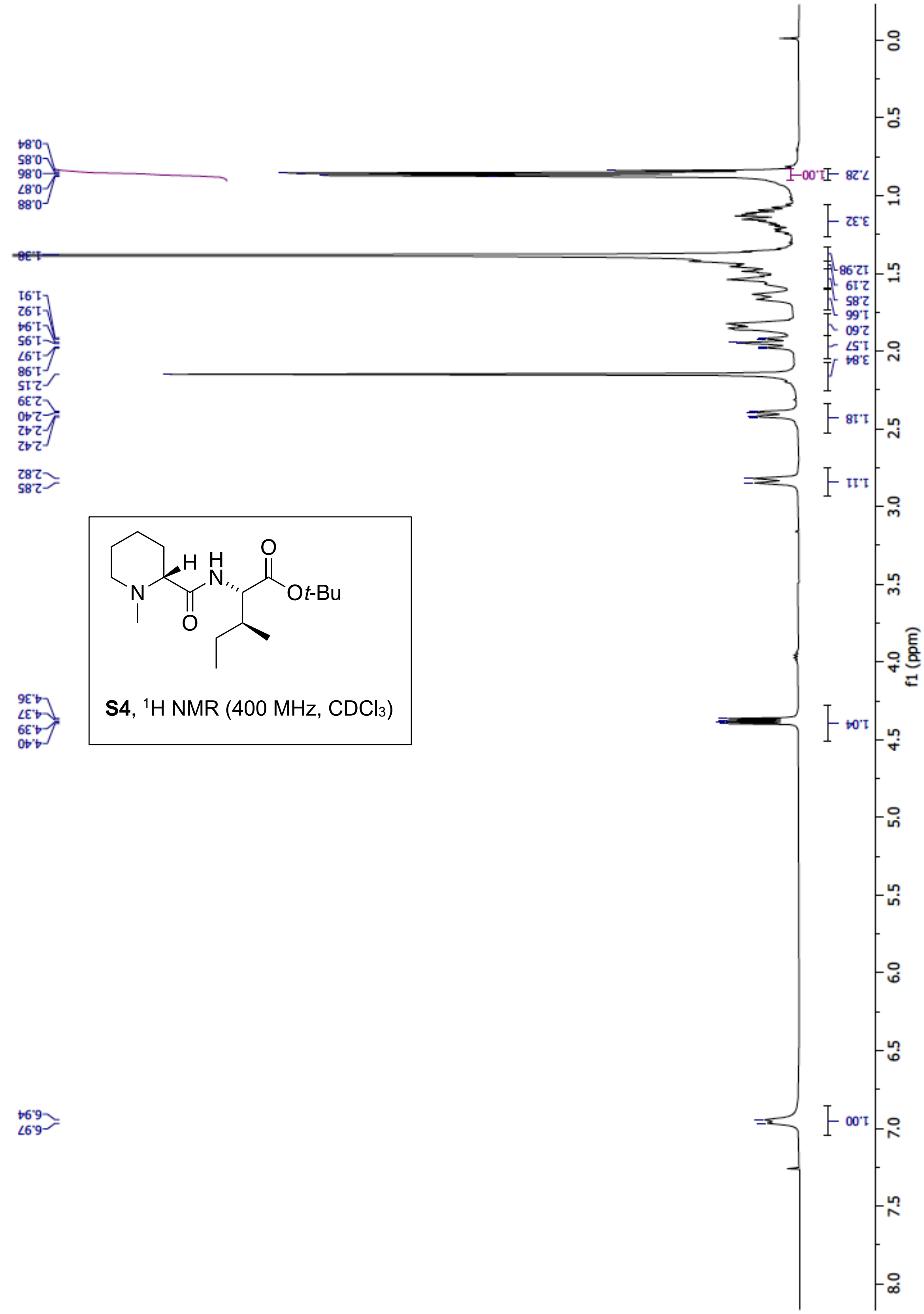

**S4**, $^1$H NMR (400 MHz, $CDCl_3$)

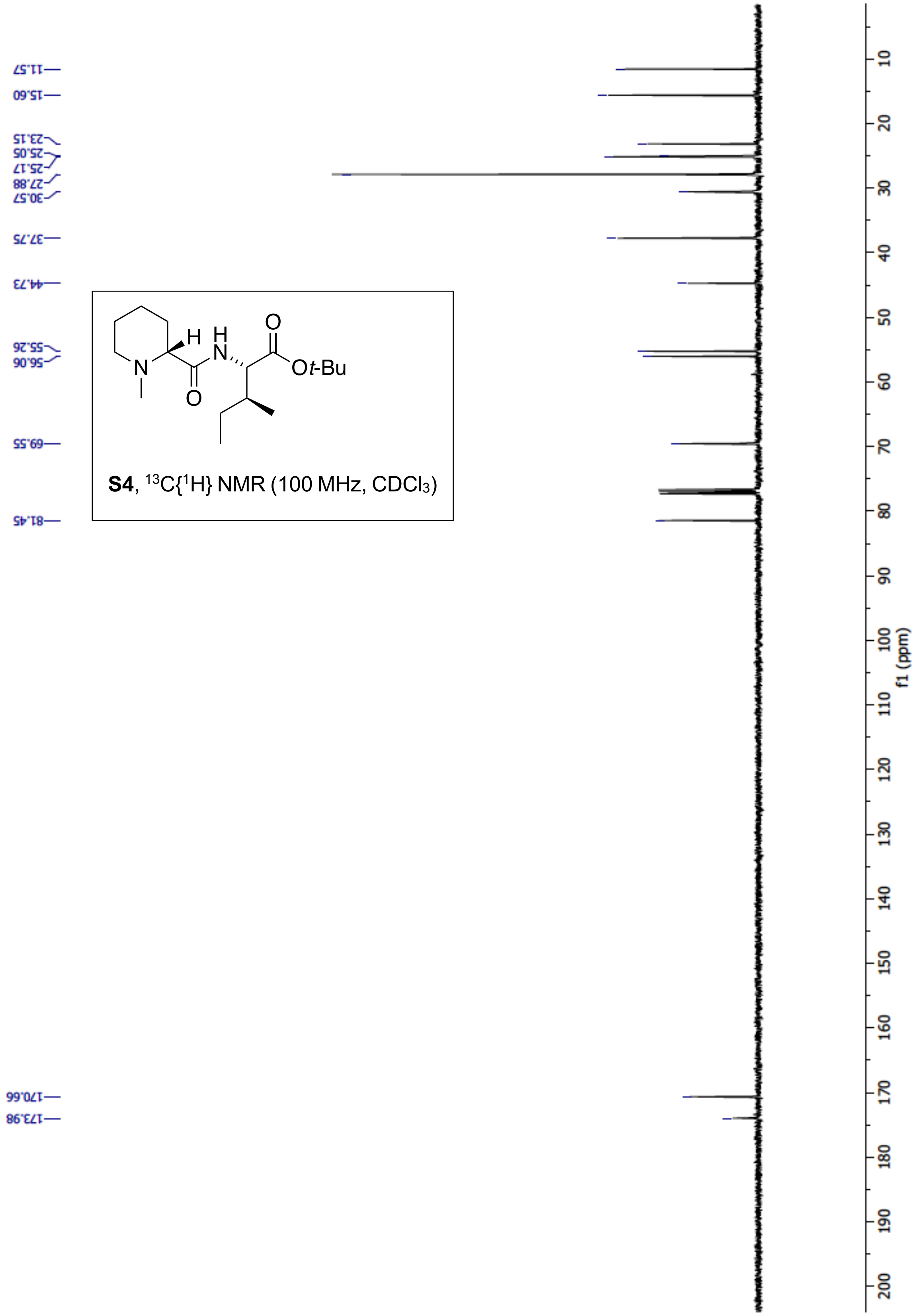


**S4**, $^{13}C\{^{1}H\}$ NMR (100 MHz, $CDCl_3$)

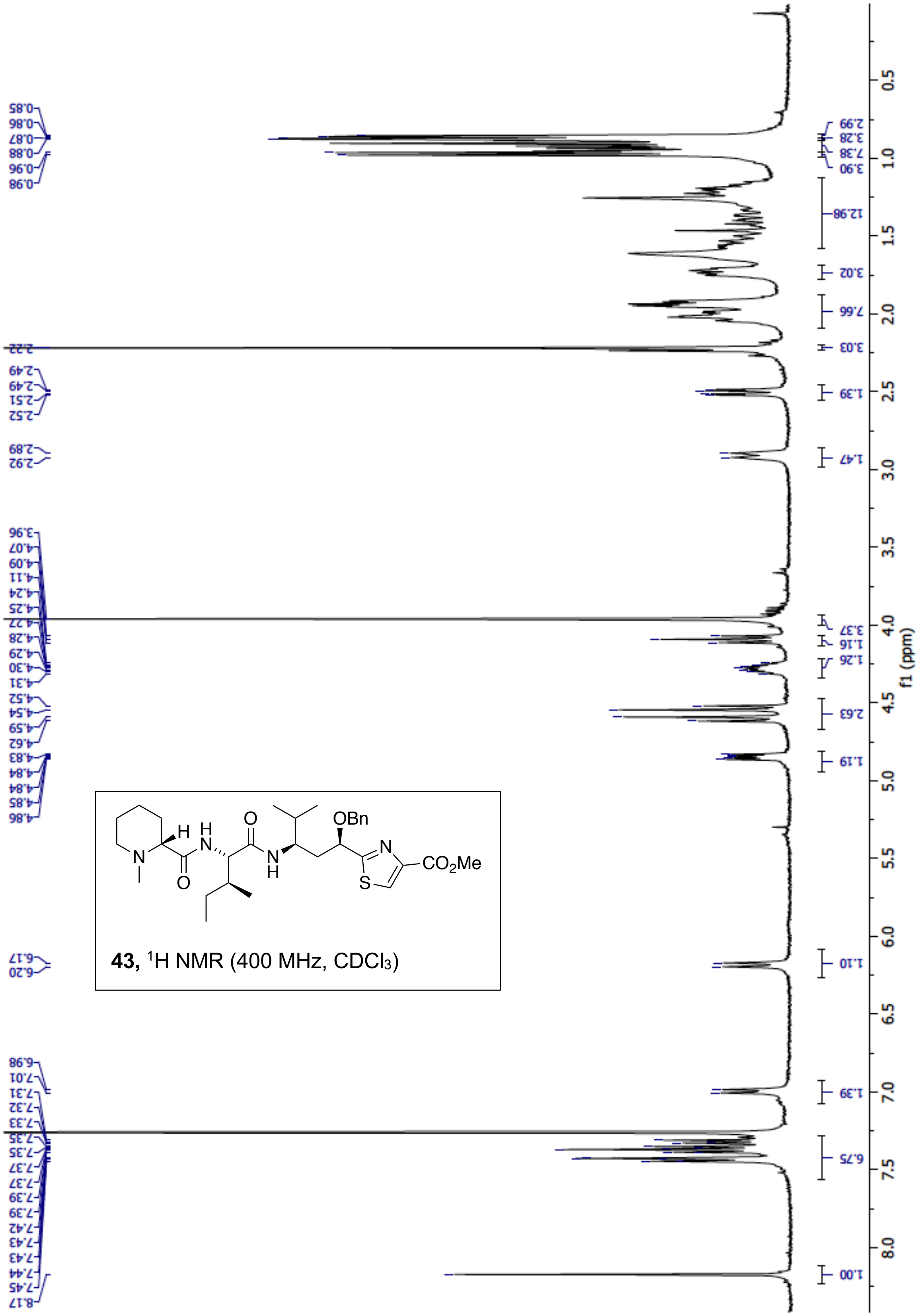


**43,** $^{1}$H NMR (400 MHz, $CDCl_3$)

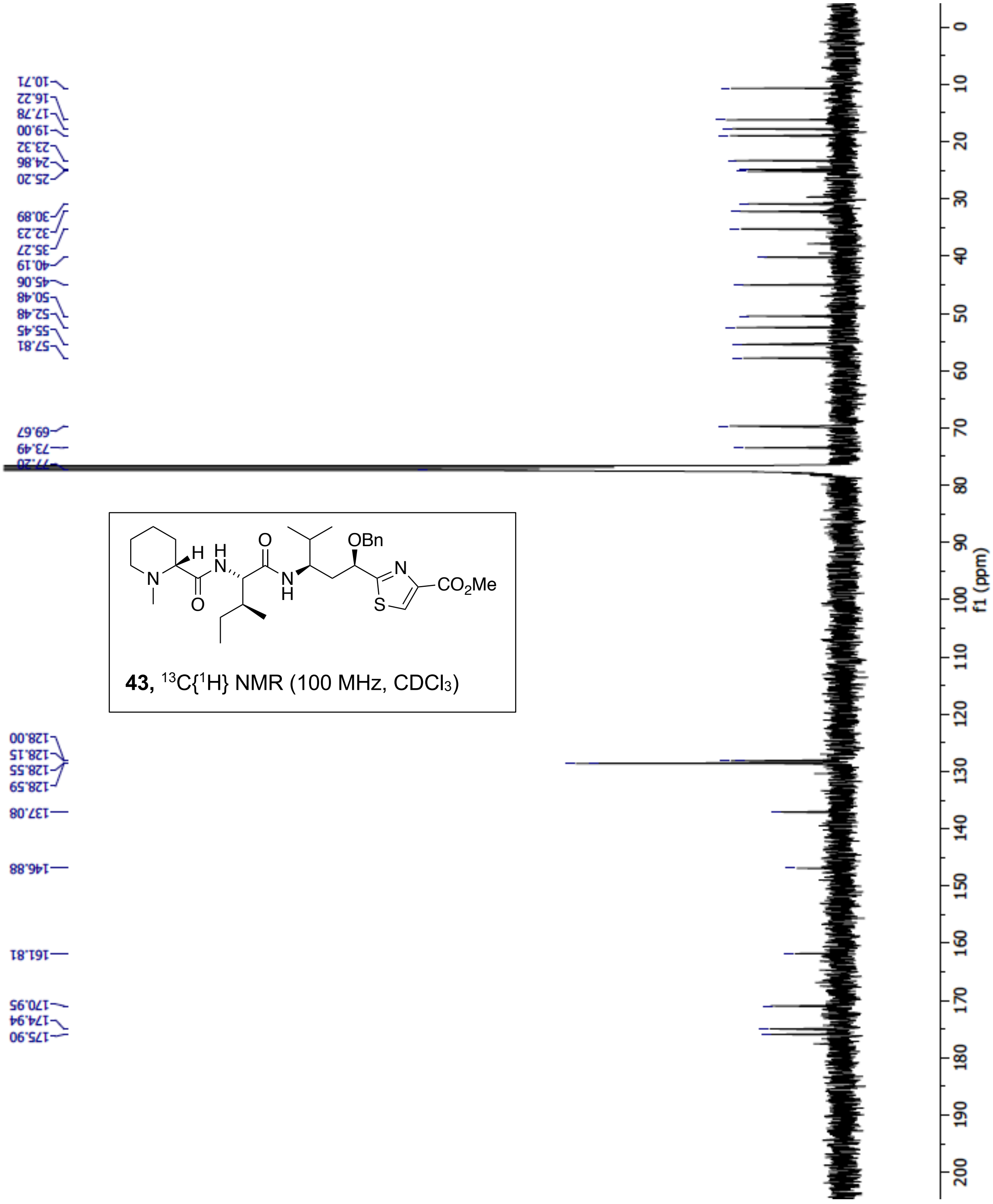


**43,** $^{13}C\{^{1}H\}$ NMR (100 MHz, $CDCl_3$)

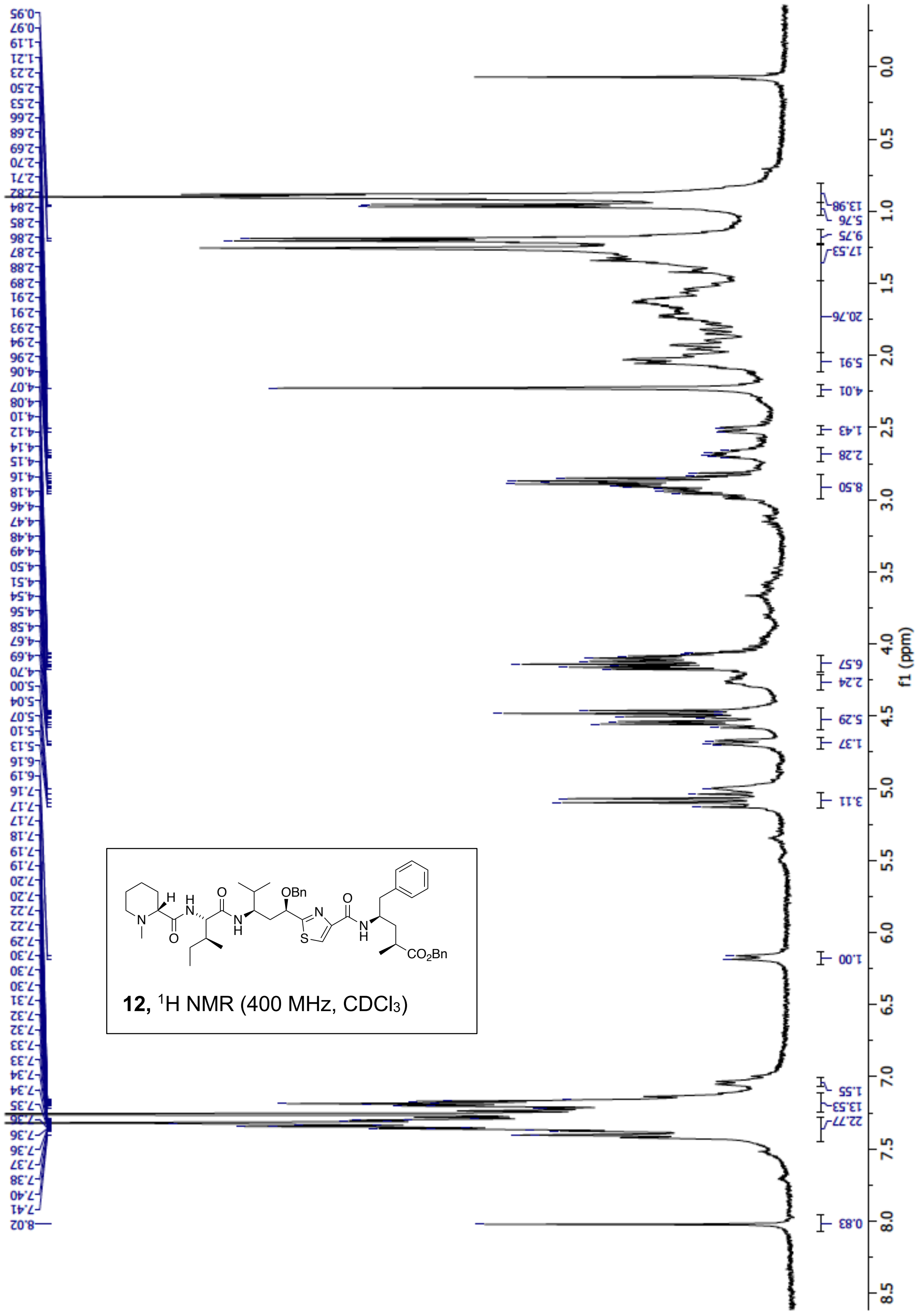


**12,** ¹H NMR (400 MHz, $CDCl_3$)

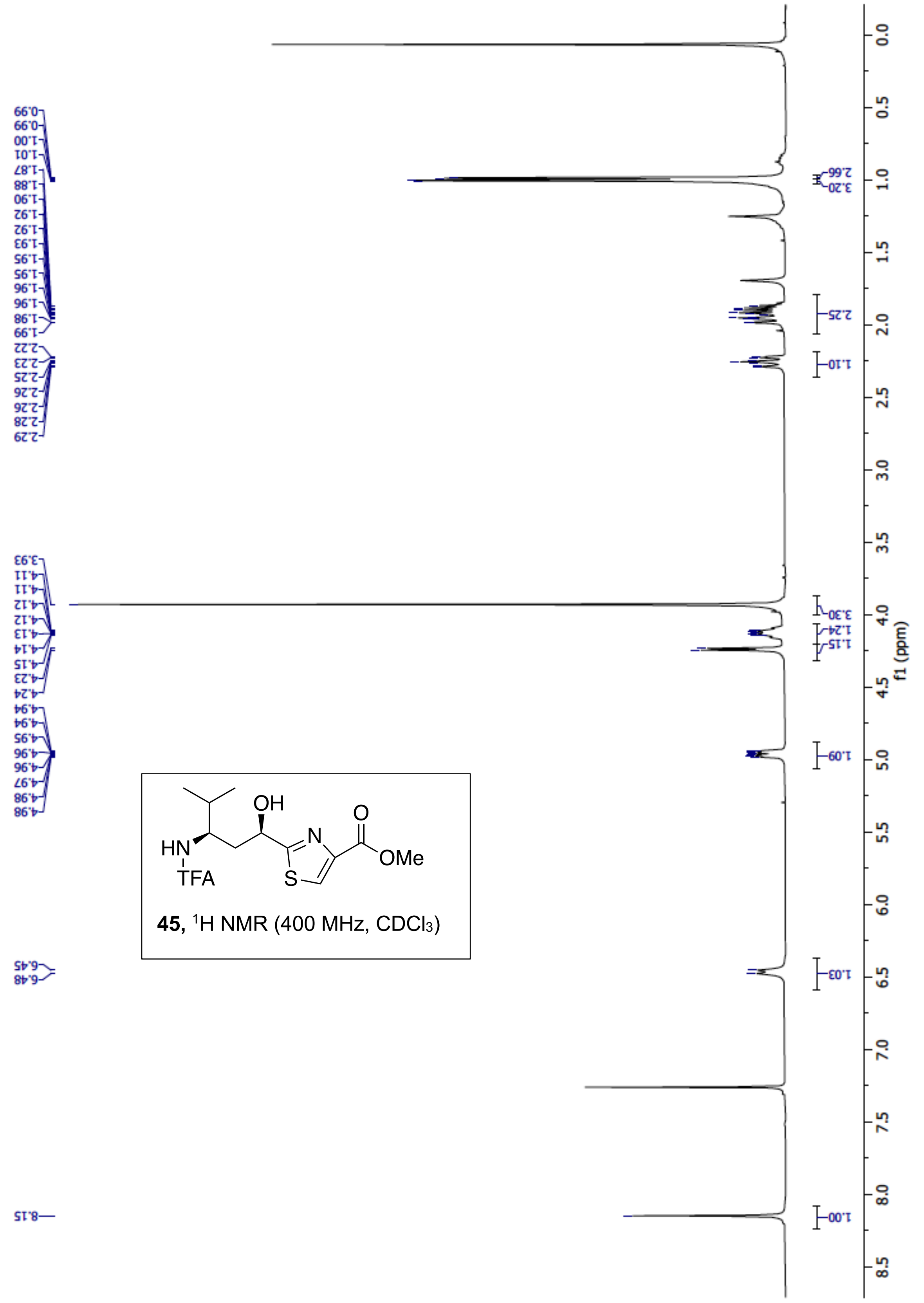


**45,** $^{1}$H NMR (400 MHz, $CDCl_3$)

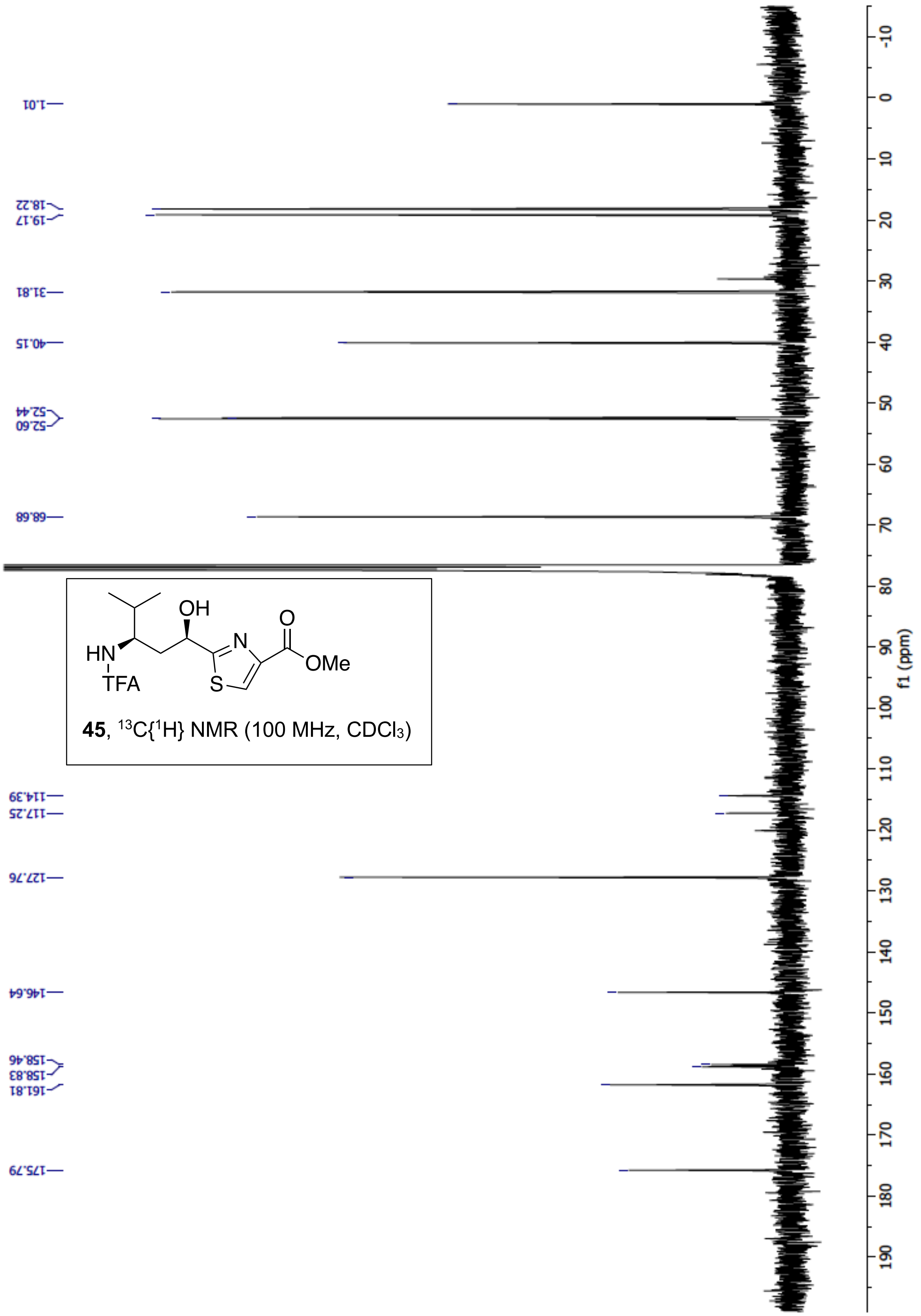


**45**, $^{13}C\{^{1}H\}$ NMR (100 MHz, $CDCl_3$)

## Enantiomer Ratio and Configuration Assignment of (*R*)-19

Compound (*R*)-**19** was obtained via Keck allylation, as shown below. The enantiomer ratio (10:1) and configuration assignment were determined via the Mosher ester prepared from (*S*)-MTPA–Cl. The $^1$H NMR spectrum is provided on the following page.

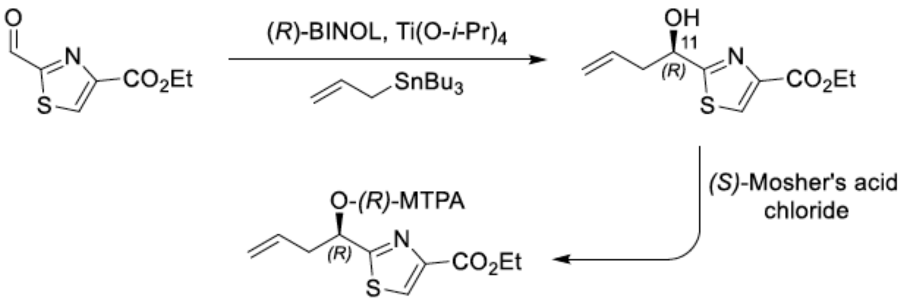


| Major diastereomer | Minor diastereomer | Δδ (=δ$_{major}$–δ$_{minor}$) ppm | Hz (400 MHz) |
|---|---|---|---|
| 8.147 | 8.087 | 0.06 | 24 |
| 4.433 | 4.423 | 0.01 | 4 |
| 1.408 | 1.400 | 0.008 | 3.2 |
| 5.70-5.60 | 5.80-5.70 | -0.10 | -40 |
| 5.08-5.00 | 5.19-5.11 | -0.11 | -44 |
| 3.542 | 3.580 | -0.038 | -15 |

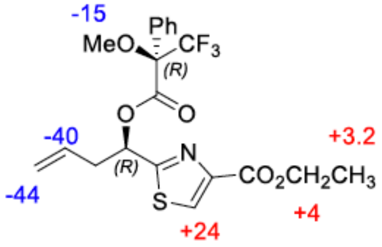

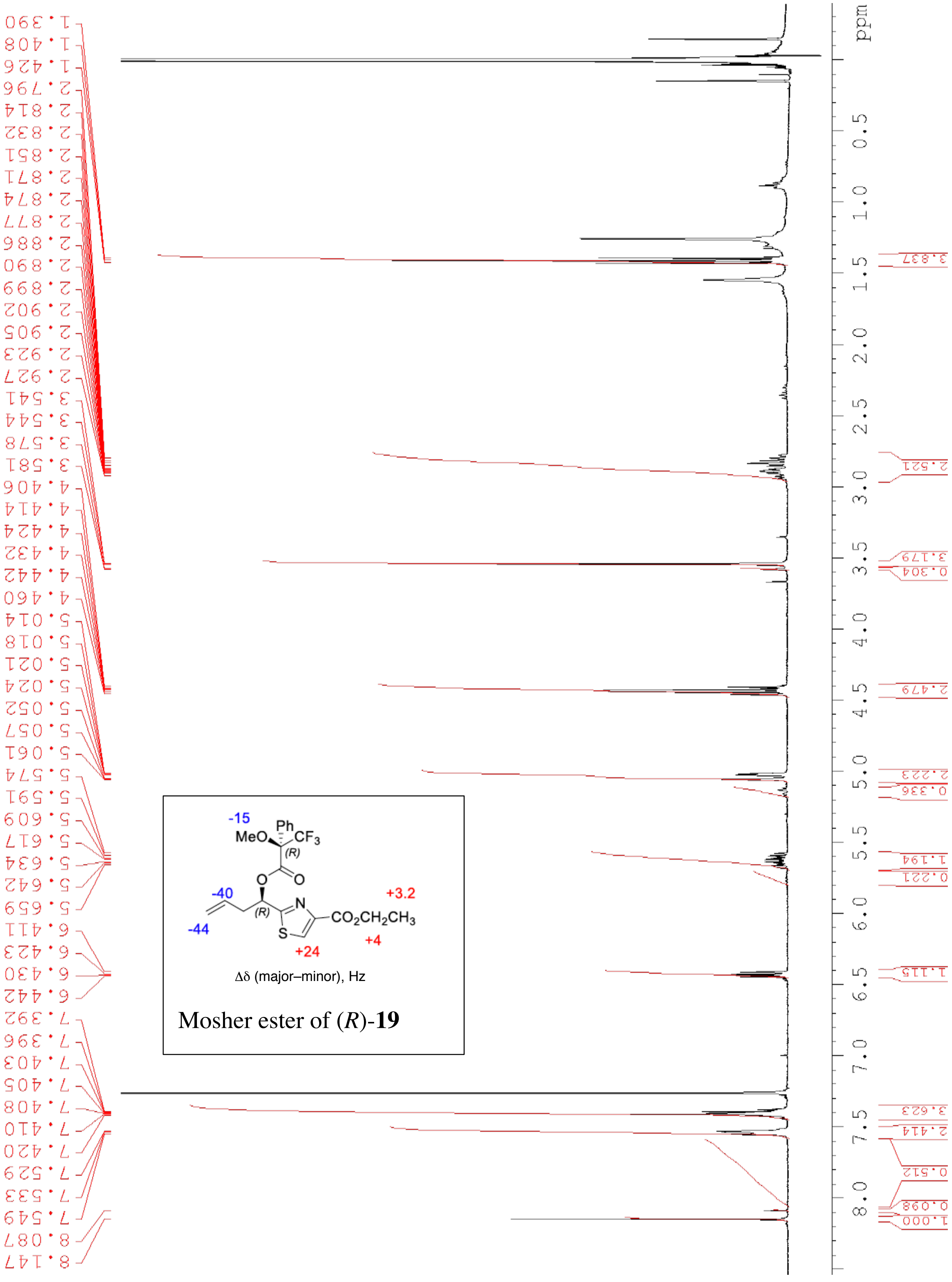

Mosher ester of (*R*)-**19**
